# Induced ectopic expression of HigB toxin in Mycobacterium tuberculosis results in growth inhibition, reduced abundance of a subset of mRNAs and cleavage of tmRNA

**DOI:** 10.1111/mmi.12358

**Published:** 2013-08-23

**Authors:** Dorothée L Schuessler, Teresa Cortes, Amanda S Fivian-Hughes, Kathryn E A Lougheed, Evelyn Harvey, Roger S Buxton, Elaine O Davis, Douglas B Young

**Affiliations:** Division of Mycobacterial Research, MRC National Institute for Medical ResearchThe Ridgeway, Mill Hill, London, NW7 1AA, UK

## Abstract

In *Mycobacterium tuberculosis*, the genes Rv1954A–Rv1957 form an operon that includes Rv1955 and Rv1956 which encode the HigB toxin and the HigA antitoxin respectively. We are interested in the role and regulation of this operon, since toxin–antitoxin systems have been suggested to play a part in the formation of persister cells in mycobacteria. To investigate the function of the *higBA* locus, effects of toxin expression on mycobacterial growth and transcript levels were assessed in *M. tuberculosis* H37Rv wild type and in an operon deletion background. We show that expression of HigB toxin in the absence of HigA antitoxin arrests growth and causes cell death in *M. tuberculosis*. We demonstrate HigB expression to reduce the abundance of IdeR and Zur regulated mRNAs and to cleave tmRNA in *M. tuberculosis*, *Escherichia coli* and *Mycobacterium smegmatis*. This study provides the first identification of possible target transcripts of HigB in *M. tuberculosis*.

## Introduction

Tuberculosis (TB) is a major global health threat. According to the WHO, TB causes 1.4 million deaths each year, and one-third of the world's population is believed to be latently infected (WHO, 2009). Treatment of TB involves a combination of four drugs (isoniazid, rifampicin, ethambutol and pyrazinamide) which are taken for 2 months in an intensive phase, followed by 4 months of isoniazid and rifampicin in a continuation phase (WHO, 2010). However, the drug treatments currently in use mainly attack actively growing bacteria and it is believed that a subpopulation of bacteria is able to evade drug-mediated killing by entering a state of non-replicating persistence (Wayne and Hayes, 1996). This persister population has been suggested to be the cause of relapse following drug treatment or reactivation of disease after years of latency (Mitchison, 2006; Warner and Mizrahi, 2006; Garton *et al*., 2008; Keren *et al*., 2011). Chromosomal toxin–antitoxin systems (TAS) can contribute to persister-mediated drug tolerance in bacteria as shown in a number of studies recently reviewed by Lewis (Lewis, 2010). TAS contain a toxin which causes growth arrest by inhibiting crucial cellular processes; for example RelE toxin inhibits translation in *Escherichia coli* (Pedersen *et al*., 2002). Toxin action is neutralized by a cognate antitoxin (Gerdes *et al*., 2005), and antitoxin generally acts as a transcriptional repressor of TA loci. Stress conditions such as starvation, DNA damage, heat shock or oxidative stress can activate TAS expression (Hazan *et al*., 2004; Christensen-Dalsgaard *et al*., 2010). This occurs through antitoxin degradation by cellular proteases such as Lon or Clp (Christensen *et al*., 2001; Christensen and Gerdes, 2003; 2004[Bibr b13],[Bibr b14]; Maisonneuve *et al*., 2011), releasing the biologically active toxin and allowing transcription of the TAS operon. Subsequent toxin-mediated growth arrest is believed to be beneficial to the bacteria, preserving nutrients and energy in an unfavourable environment and allowing resumption of growth when conditions have become favourable again (Gerdes *et al*., 2005; Hayes and Van Melderen, 2011).

Toxin–antitoxin systems are ubiquitous in mycobacteria, with members of the VapBC, MazEF and RelBE and ParDE as well as novel families of TAS found across the genus. Interestingly, there seems to have been an expansion of VapBC, MazEF and RelE TA families in the *Mycobacterium tuberculosis* complex (MTBC) which includes *M. tuberculosis*, *Mycobacterium bovis, Mycobacterium africanum*, *Mycobacterium canetti*, *Mycobacterium microti* and *M. bovis* Bacille-Calmette-Guerin. This is not the case for HigBA, where only one locus is present in each of the members of the MTBC (Pandey and Gerdes, 2005; Ramage *et al*., 2009). The HigBA locus was first identified on the Rst1 plasmid (Tian *et al*., 1996), but homologues have subsequently been found on chromosomes of a range of bacteria including clinical isolates of *Pseudomonas aeruginosa* and methicillin-resistant *Staphylococcus aureus* (Pandey and Gerdes, 2005; Williams *et al*., 2011). HigB toxins belong to the RelE family and have been characterized as translation-dependent mRNA-cleaving enzymes in *Vibrio cholerae*, *Proteus vulgaris* and *E. coli* (Christensen-Dalsgaard and Gerdes, 2006; Hurley and Woychik, 2009; Christensen-Dalsgaard *et al*., 2010). HigB toxins are able to cleave a wide range of mRNAs (Christensen-Dalsgaard and Gerdes, 2006; Hurley and Woychik, 2009; Christensen-Dalsgaard *et al*., 2010) and associate with the 50S subunit of the ribosome in *P. vulgaris* (Hurley and Woychik, 2009). Both amino acid starvation and chloramphenicol-mediated inhibition of protein synthesis induce the *higBA* loci of *V. cholerae* and *E. coli* (Christensen-Dalsgaard and Gerdes, 2006; Christensen-Dalsgaard *et al*., 2010). The activation of HigB toxin by HigA degradation is Lon protease-dependent in *E. coli* (Christensen-Dalsgaard *et al*., 2010).

**Figure 1:**
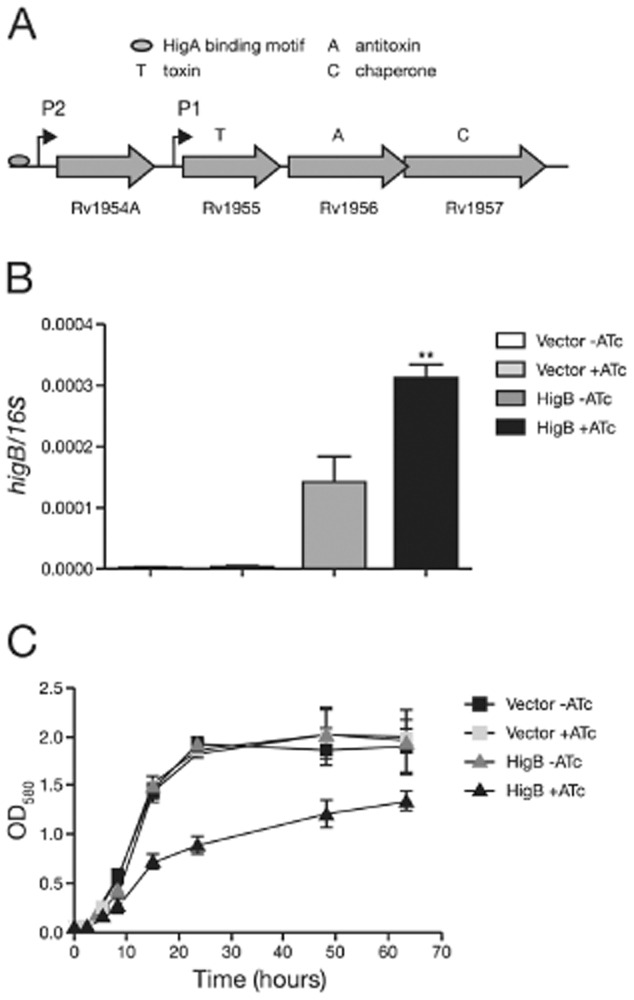
HigB expression in *M. smegmatis*.A. Genomic organization of the *M. tuberculosis* HigBA locus.B and C. *M. smegmatis* strains carrying vector control (‘Vector’) or the HigB expression plasmid (‘HigB’) grown in the absence (−) or presence (+) of anhydrotetracycline (ATc). (B) Quantitative RT-PCR of *higB* expression relative to 16S rRNA. RNA was isolated from exponential-phase cultures. (C) Growth of *M. smegmatis* strains. Liquid cultures of transformants were grown from a starting OD_580_ of 0.05 and monitored over time. No or 300 ng ml^−1^ ATc were added at the start of the growth curve. All results are the mean values and standard deviation of three independent biological replicates. Where indicated, a significant difference (as determined by Student's *t*-test) between uninduced (− ATc) and induced (+ ATc) conditions is marked by an asterisk (*) for *P* < 0.05, ** for *P* < 0.01, *** for *P* < 0.0001.

The *M. tuberculosis* HigBA TAS has previously been shown to be a functional toxin–antitoxin system in *E. coli* and mycobacteria (Gupta, 2009; Fivian-Hughes and Davis, 2010; Bordes *et al*., 2011). The HigBA TAS of *M. tuberculosis* is unusual in terms of its genomic organization. The HigB toxin (Rv1955) and HigA antitoxin (Rv1956) are in an operon that includes Rv1954A and Rv1957 (Smollett *et al*., 2009; Fivian-Hughes and Davis, 2010). Rv1957 has recently been identified as a Sec-B like chaperone required for antitoxin stabilization (Bordes *et al*., 2011). Rv1954A shows homology with a family of YjzC-like proteins that are widely conserved in bacteria but its function remains unknown. The Rv1954A-1957 operon is regulated by two promoters (Fig. 1A). P1, located directly upstream of HigB, is induced by DNA damage and is not regulated by HigA (Fivian-Hughes, 2009), while P2, upstream of Rv1954A, is repressed by binding of HigA to a specific motif (Fivian-Hughes and Davis, 2010).

The *higBA* locus is induced by DNA damage, heat shock, and during hypoxia and growth in activated macrophages, indicating that it might be important for bacterial survival under stress conditions encountered during infection (Stewart *et al*., 2002; Gamulin *et al*., 2004; Ramage *et al*., 2009; Homolka *et al*., 2010). We are interested in the function of this operon in *M. tuberculosis*, and its potential role in mycobacterial persistence (Singh *et al*., 2010). We have analysed the effect of expressing *higB* under the control of an inducible promoter system, characterized the effect of *higB* induction on global translation and identified cleavage of tmRNA in response to HigB expression.

## Results

### Expression of HigB inhibits mycobacterial growth

Toxin activity of *M. tuberculosis* HigB has previously been demonstrated in both *E. coli* and *Mycobacterium marinum* (Gupta, 2009; Bordes *et al*., 2011). In *M. tuberculosis* itself, HigB toxicity has been inferred from an inability to delete HigA antitoxin without simultaneous deletion of HigB toxin (Fivian-Hughes and Davis, 2010). To test directly the effect of HigB on growth of *M. tuberculosis* we constructed an inducible expression plasmid, in which *higB* was placed under the control of a tetracycline-inducible promoter. Functionality of this system was initially tested in *Mycobacterium smegmatis*, which does not contain a HigBA locus (Ramage *et al*., 2009).

*Mycobacterium smegmatis* was electroporated with the HigB-expression plasmid and the vector control. Transformants were grown to mid-exponential phase in liquid media with and without anhydrotetracycline (ATc) inducer. Quantitative RT-PCR was used to measure the level of *higB* transcripts present in the different strains in the absence and presence of inducer (Fig. 1B). As expected, no *higB* was detected in the vector control strain. In the HigB-expression strain, some transcripts were detected in the absence of ATc, indicating a degree of leakage associated with the construct; expression increased twofold in the presence of inducer (Fig. 1B). These results confirm the functionality of the expression system used.

We proceeded to determine the effect of *higB* expression on growth in liquid media. Bacterial growth was measured by the optical density of the cultures in the presence or absence of inducer (Fig. 1C). No difference was observed during the initial 5 h of growth. However, after 7.5 h in the presence of inducer, growth of strains harbouring the HigB-expression plasmid slowed down and was significantly lower than growth of control strains over a 60 h time-course (Fig. 1C). These results show that *higB* expression inhibits growth of *M. smegmatis*. Furthermore our data suggest a sharp threshold for HigB toxicity, given that a twofold induction was sufficient to cause growth inhibition.

**Figure 2:**
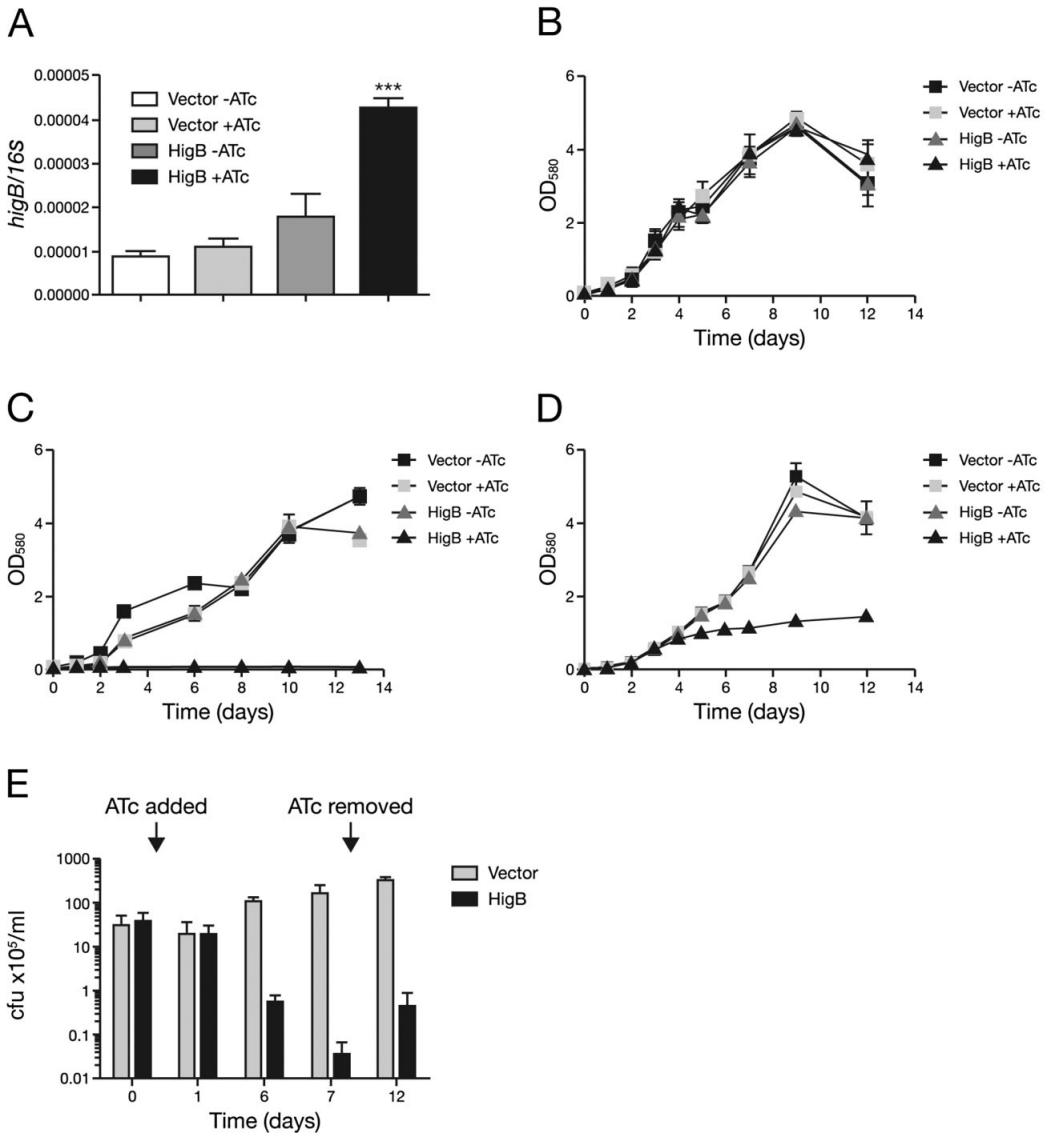
HigB expression in *M. tuberculosis. M. tuberculosis* strains carrying vector control (‘Vector’) or the HigB expression plasmid (‘HigB’) grown in the absence (−) or presence (+) of ATc.A. Quantitative RT-PCR of *higB* expression relative to 16S rRNA. RNA was isolated from *M. tuberculosis* wild-type exponential-phase cultures.B–D. Growth of *M. tuberculosis* wild-type (B) and ΔTAC (C and D) strains. Liquid cultures of transformants were grown from a starting OD_580_ of 0.05 and monitored over time. No or 300 ng ml^−1^ ATc were added at the start of the growth curve at day 0 (B and C) or during mid-exponential phase at day 3 (D).E. Survival of ΔTAC transformants following addition of ATc during mid-exponential phase (time 0) and removal of inducer by washing at day 7.All results are the mean values and standard deviation of three independent biological replicates. A significant difference (as determined by Student's *t*-test) between uninduced (− ATc) and induced (+ ATc) conditions is marked by an asterisk (*) for *P* < 0.05, ** for *P* < 0.01, *** for *P* < 0.0001.

Next, the effects of toxin expression were tested in *M. tuberculosis*. Plasmids were electroporated into *M. tuberculosis* H37Rv and transformants were grown to mid-exponential phase with and without ATc. To confirm that addition of ATc resulted in *higB* induction in *M. tuberculosis* the number of *higB* transcripts was measured by qRT-PCR in vector control and conditional-expression strains with and without inducer (Fig. 2A). The level of expression of *higB* relative to a 16S control was significantly higher in the strains containing the expression plasmid than in the vector control, with a 4.6-fold increase in the presence of ATc, confirming the functionality of this expression system in *M. tuberculosis* (Fig. 2A).

Next, we monitored growth of liquid cultures in the presence or absence of inducer (Fig. 2B). Both the vector control and the HigB expression strains grew at similar rates over a period of 14 days (Fig. 2B). Thus toxin expression in *M. tuberculosis* wild type does not result in a growth defect.

Having validated that ATc addition resulted in increased *higB* expression, we reasoned that the lack of an effect on growth may be due to neutralizing activity of endogenous antitoxin and chaperone present in *M. tuberculosis* wild type. We therefore tested the effect of *higB* expression on growth in a Rv1955–Rv1957 (ΔTAC) deletion mutant (Fivian-Hughes and Davis, 2010). Growth was seen in the vector control strain and in the uninduced expression strain over the course of 14 days (Fig. 2C). In contrast no growth was observed when the expression strain was inoculated into medium containing ATc (Fig. 2C). This shows that, in the absence of neutralizing antitoxin and chaperone, HigB expression inhibits early exponential growth.

We then investigated the effect of addition of inducer during mid-exponential phase (Fig. 2D). As before, no difference in growth was seen between the vector control strain and the uninduced expression strain (Fig. 2D). However, 1 day after ATc induction of the expression strain, optical densities ceased to increase at the same rate as in the control strains, and did not go beyond OD_580_ 1.4 (Fig. 2D). Thus HigB expression during mid-exponential phase resulted in growth inhibition. Associated loss of viability was evident from analysis of colony-forming units (cfu) following ATc addition. Colony-forming units decreased by 3 log_10_ 7 days after ATc addition to the HigB-expression strain (Fig. 2E). In contrast, cfu continued to increase in the ATc-treated vector control strain, confirming that the inducer itself had no bactericidal effect (Fig. 2E). Subsequent removal of inducer resulted in resumption of growth of the remaining viable population, as seen by 1 log_10_ increase in cfu 5 days after ATc was removed from the cells by washing (Fig. 2E). Together these data demonstrate that *higB* expression in the absence of antitoxin and Rv1957c chaperone arrests growth and causes substantial cell death in *M. tuberculosis*.

### Global gene expression profiling identifies putative HigB targets

To investigate the effect of HigB expression on global transcriptional profiles we carried out RNAseq analysis. RNA was extracted from HigB expressing and vector control strains 24 h after ATc addition; libraries were prepared, sequenced and analysed. Validation of RNAseq results by means of qRT-PCR is recorded in Table S1.

The relative abundance of 34 mRNAs changed significantly (Table 1). Two genes, Rv0197 (a non-essential gene of unknown function) and *bfrB* (a non-essential IdeR regulated gene involved in iron storage), were upregulated (Gold *et al*., 2001; Sassetti *et al*., 2003). Of the 32 downregulated genes, a large number are part of operons regulated by IdeR (*mbtK*, *irtAB,hisE, PPE37, mbtHFEDCBAI*, Rv3403c, Rv3839), Zur (PPE3) or both (*eccB3, eccC3, esxG, esxH, espG3, eccD3, mycP3, eccE3* and *mmpL5*) (Gold *et al*., 2001; Rodriguez *et al*., 2002; Maciag *et al*., 2007). qRT-PCR confirmed downregulation of *espG3* and *mbtC* in the HigB expression strain, and also highlighted reduced abundance of *rpmE*, which had not reached statistical significance in the RNAseq analysis (Fig. S1). RpmE has been implicated in zinc homeostasis in *Bacillus subtilis* (Nanamiya *et al*., 2006). In addition to the metal ion regulons, we also observed a significant decrease in abundance of tmRNA transcripts. Parallel microarray analysis generated analogous results with significant fold change observed for a limited subset of genes enriched in metal ion regulons (data not shown).

**Table 1:** RNAseq data comparing fold change between the HigB expressing and the vector control strains

Locus tag	Gene name	Function	Fold change	*P*adj	Essentiality^a^
Rv0106	*Rv0106*	Conserved hypothetical	0.2	3.4E-04	NE
Rv0197	*Rv0197*	Intermediary metabolism and respiration	3.3	2.3E-02	NE
Rv0280	*PPE3*	PE/PPE	0.3	1.2E-04	NE
Rv0283	*eccB3*	Cell wall and cell processes	0.4	4.5E-02	E
Rv0284	*eccC3*	Cell wall and cell processes	0.4	4.4E-02	E
Rv0287	*esxG*	Cell wall and cell processes	0.4	2.5E-02	NE
Rv0288	*esxH*	Cell wall and cell processes	0.3	1.1E-02	NE
Rv0289	*espG3*	Cell wall and cell processes	0.3	1.3E-02	E
Rv0290	*eccD3*	Cell wall and cell processes	0.3	3.9E-03	E
Rv0291	*mycP3*	Intermediary metabolism and respiration	0.3	5.0E-03	E
Rv0292	*eccE3*	Cell wall and cell processes	0.3	1.3E-02	E
Rv0676c	*mmpL5*	Cell wall and cell processes	0.3	6.3E-03	NE
Rv0678	*Rv0678*	Conserved hypothetical	0.3	3.4E-04	NE
Rv0860	*fadB*	Lipid metabolism	0.4	4.0E-02	NE
Rv0973c	*accA2*	Lipid metabolism	0.2	1.4E-03	E
Rv0974c	*accD2*	Lipid metabolism	0.3	3.4E-02	NE
Rv1347c	*mbtK*	Lipid metabolism	0.2	2.2E-04	E
Rv1348	*irtA*	Cell wall and cell processes	0.2	2.6E-06	E
Rv1349	*irtB*	Cell wall and cell processes	0.1	2.4E-08	E
Rv2121c	*hisG*	Intermediary metabolism and respiration	0.2	4.6E-02	E
Rv2122c	*hisE*	Intermediary metabolism and respiration	0.2	2.7E-02	E
Rv2123	*PPE37*	PE/PPE	0.0	2.6E-13	NE
Rv2377c	*mbtH*	Lipid metabolism	0.2	2.7E-04	NE
Rv2378c	*mbtG*	Lipid metabolism	0.1	5.1E-08	E
Rv2379c	*mbtF*	Lipid metabolism	0.2	2.4E-08	NE
Rv2380c	*mbtE*	Lipid metabolism	0.2	2.6E-07	NE
Rv2381c	*mbtD*	Lipid metabolism	0.2	4.4E-08	NE
Rv2382c	*mbtC*	Lipid metabolism	0.2	1.4E-06	E
Rv2383c	*mbtB*	Lipid metabolism	0.2	4.4E-08	NE
Rv2384	*mbtA*	Lipid metabolism	0.3	2.7E-02	NE
Rv2386c	*mbtI*	Lipid metabolism	0.1	3.6E-08	E
Rv2821c	*Rv2821c*	Cell wall and cell processes	0.1	2.4E-08	NE
Rv3403c	*Rv3403c*	Conserved hypothetical	0.2	8.7E-05	NE
Rv3839	*Rv3839*	Conserved hypothetical	0.1	3.6E-08	NE
Rv3841	*bfrB*	Intermediary metabolism and respiration	17.2	1.1E-12	NE
Rv1955	*higB*	Virulence, detoxification, adaptation	1.0	9.2E-01	NE
rRNA-ssrA	*ssrA*		0.5	7.4E-01	E

a E, essential; NE, non-essential. Essentiality is taken from Sassetti *et al*. (2003) and Zhang *et al*. (2012).

### M. tuberculosis  HigB expression causes tmRNA degradation

HigB is related to the RelE family of toxins that are known to target mRNA as well as tmRNA, the stable RNA product of the *ssrA* gene discovered by Ray and Apirion (Ray and Apirion, 1979; Pandey and Gerdes, 2005). tmRNA plays an essential role in *trans*-translation, which is required to rescue ribosomes stalled by RelE-generated non-stop mRNAs (Christensen *et al*., 2003). A role for *trans*-translation in *M. tuberculosis* persistence has been highlighted by the demonstration that this process is inhibited by the antimycobacterial drug pyrazinamide (Shi *et al*., 2011). We were therefore particularly interested to determine whether *M. tuberculosis* HigB also targets tmRNA.

**Figure 3:**
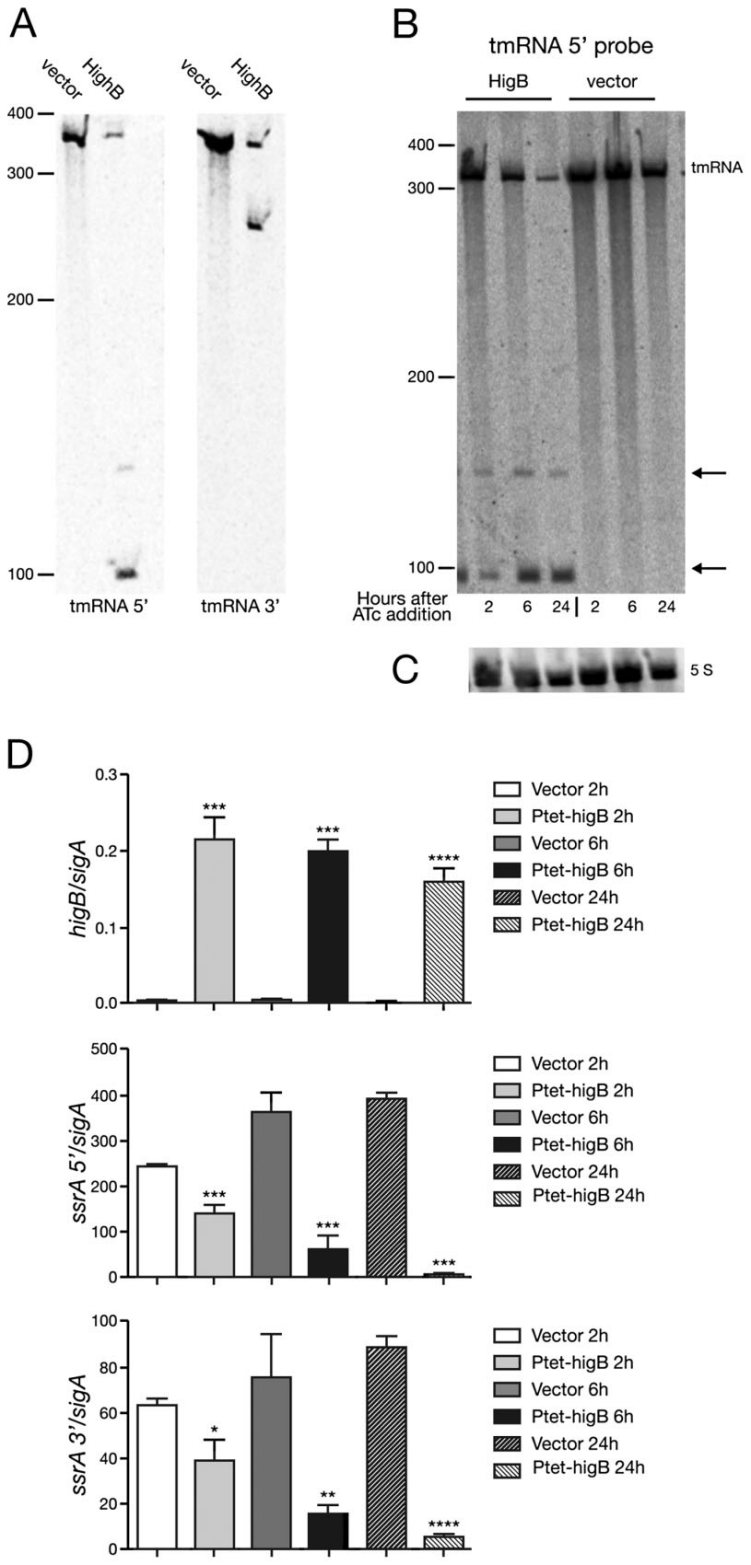
HigB expression affects tmRNA in *M. tuberculosis* ΔTAC. RNA was extracted from mid-exponential cultures treated with ATc for 2 h, 6 h or 24 h and cDNA was prepared for qRT-PCR analysis.A–C. Northern blots probing for tmRNA (A and B) and 5S (C). Transcript sizes are relative to the position of RNA marker and tmRNA cleavage products are indicated with an arrow.D. Quantitative RT-PCR of transcripts of interest. All results are the mean values and standard deviation of three independent biological replicates. A significant difference (as determined by Student's *t*-test) between vector control and HigB overexpression strain is marked by an asterisk (*) for *P* < 0.05, (**) for *P* < 0.01, (***) for *P* < 0.0001.

Total RNA was extracted from *M. tuberculosis* ΔTAC strains in mid-exponential growth phase at 2, 6 and 24 h after toxin induction and probed by Northern blot probing for the 5′ and 3′ ends of tmRNA. Only the expected full-length tmRNA transcript was seen in the vector control (Fig. 3A). In contrast, the amount of the full-length transcript decreased following induction of HigB in the expression strain, along with appearance of two cleaved fragments of ∼ 100 bp and ∼ 150 bp when probing for 5′ tmRNA, and one cleaved fragment of ∼ 250 bp when probing for 3′ tmRNA (Fig. 3A). Furthermore, the amount of cleavage product increased with time after HigB induction (Fig. 3B). Toxin induction had no effect on the integrity of ribosomal 5S RNA (Fig. 3C).

To quantify the effect of HigB expression on tmRNA, qRT-PCR was carried out (Fig. 3D). As expected in the ΔTAC background, significant expression of *higB* was only detected in the conditional-expression strain (Fig. 3D top panel). HigB was fully induced by 2 h after ATc addition. Quantification using primers directed to either the 5′ or the 3′ end of tmRNA revealed a 1.5-fold increase in relative expression after addition of ATc to the vector strain (Fig. 3D middle and bottom), consistent with a previous observation that tetracycline upregulates expression of tmRNA in *M. smegmatis* (Andini and Nash, 2011). In contrast to this, the relative number of transcripts detected using the 5′ and 3′ probes in the expression strain dropped sharply over the 24 h incubation period (Fig. 3D middle and bottom). From these results, we conclude that the initial cleavage fragments detected by Northern blot are progressively degraded over time.

**Figure 4:**
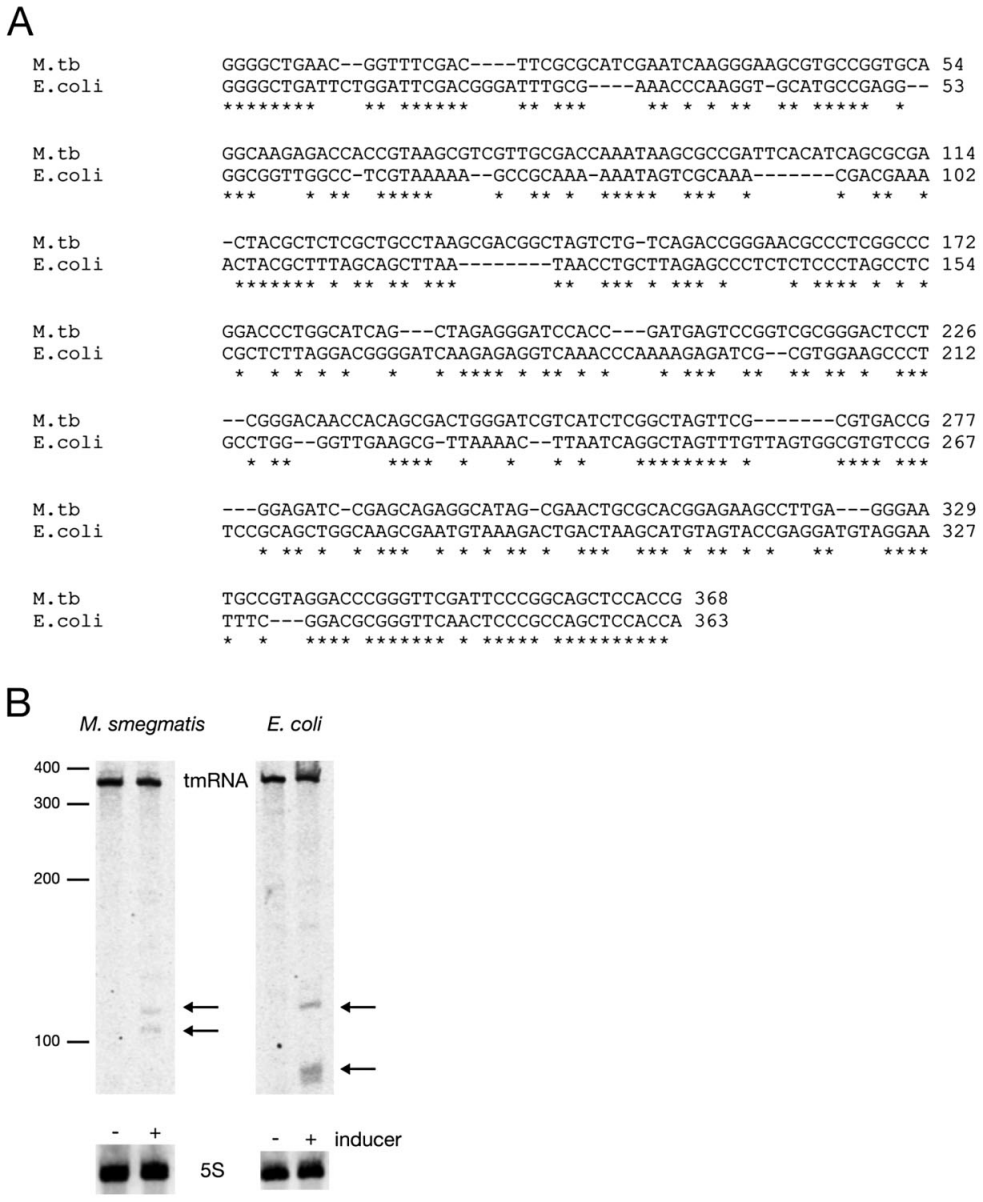
HigB expression affects tmRNA in *M. smegmatis* and *E. coli*.A. DNA alignment of *ssrA* from *M. tuberculosis* and *E. coli*. Conserved residues are highlighted by an asterisk (*).B. Northern blots probing for tmRNA (B) and 5 S (C). RNA was extracted from *M. smegmatis* (left panel) and *E. coli* (right panel) HigB expression strains grown with or without inducer. Strains were grown to mid-exponential phase and inducer was added; ATc for *M. smegmatis* and l-arabinose for *E. coli*, for 7.5 h and 2 h respectively.

tmRNA shares high sequence similarity between *M. tuberculosis* and *E. coli* (Fig. 4A), and could be a conserved target of HigB. We tested if HigB also causes tmRNA cleavage in *M. smegmatis* and *E. coli*. RNA was prepared from mid-log phase bacteria harbouring HigB-expression plasmid grown in the presence or absence of inducer. Northern blots probing for the 5′ end of tmRNA were carried out (Fig. 4B). In *M. smegmatis* (Fig. 4B left), full-length tmRNA transcript was detected in the HigB expression strain under both conditions tested; two distinct cleavage products of smaller size (> 100 bp) were seen when HigB expression is induced. Similarly in *E. coli*, full-length transcript was present along with two cleavage products when HigB was expressed (Fig. 4B right). Expression of *M. tuberculosis* HigB in *M. smegmatis* as well as *E. coli* results in tmRNA cleavage, identifying this as a conserved target of the *M. tuberculosis* toxin.

### tmRNA is cleaved in the mRNA-like region

HigB toxin expression gives rise to distinct tmRNA cleavage products, indicating that the toxin might directly cleave specific sites within the tmRNA molecule. To investigate this further by mapping the 3′ ends resulting from cleavage, we performed 3′ RACE as described previously (Arnvig and Young, 2009).

**Figure 5:**
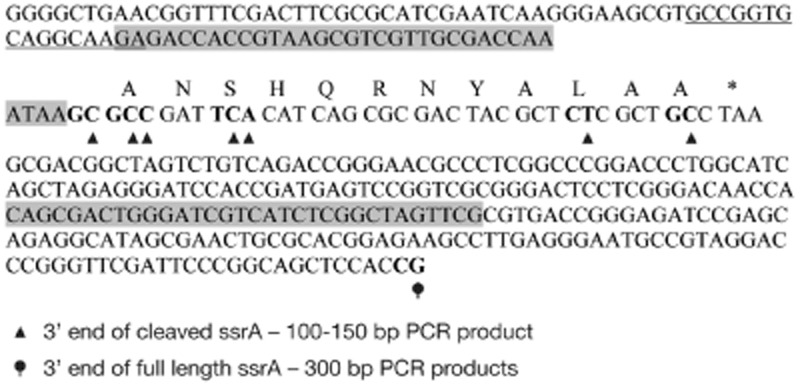
HigB cleavage sites identified by 3′ RACE. A schematic showing the DNA sequence of *ssrA*. Amino acids encoded in the mRNA like region are shown. Putative cleavage sites found in the 300 bp and 100–150 bp PCR products are indicated as highlighted 3′ residues before the A tail. Nucleotides between which the cleavage must have occurred are in bold. The sequence of the gene-specific forward primer used is underlined. The 5′ and 3′ probes used for Northern blots are shaded in grey.

RNA was prepared from *M. tuberculosis* HigB-expressing and vector control strains treated with inducer for 24 h. A poly-A tail was added and cDNA prepared. This was used as a template for PCR using a gene-specific forward primer binding tmRNA just upstream of the 5′ probe used for Northern blots (Fig. 5) and a poly-A linker-specific primer. PCR product corresponding to nearly full-length tmRNA (the gene-specific primer binds 45 bp after the start of *ssrA*) was obtained from the vector control strain. In the HigB-expression strain, an additional PCR product matching the size of the cleavage products detected by Northern blot (∼ 100–150 bp) was also obtained. PCR products were cloned into a plasmid and sequenced. The 3′ ends of transcripts were identified as the junction with the poly-A tail. All of the 3′ ends identified from the large PCR products corresponded to full-length *ssrA* (Fig. 5). Sequencing of 25 clones generated from the short PCR products (i.e. the cleaved tmRNA) identified seven 3′ ends located within the mRNA-like coding region of the tmRNA (Fig. 5). This pattern could be generated by multiple cleavage sites, or by a single cleavage followed by 5′-to-3′ exonuclease digestion.

## Discussion

Since their discovery in the 1980s, TA systems have been found in nearly all prokaryotes and archaea (Pandey and Gerdes, 2005; Georgiades and Raoult, 2011; Yamaguchi *et al*., 2011). Due to their high abundance in *M. tuberculosis*, there has been a series of research efforts investigating mycobacterial TA systems (Arcus *et al*., 2005; 2011[Bibr b5],[Bibr b4]; Zhu *et al*., 2006; 2008; 2010[Bibr b72],[Bibr b70],[Bibr b71]; Carroll *et al*., 2007; Zhao and Zhang, 2008; Gupta, 2009; Korch *et al*., 2009; Ramage *et al*., 2009; Robson *et al*., 2009; Han *et al*., 2010; Huang and He, 2010; Singh *et al*., 2010; Yang *et al*., 2010; Ahidjo *et al*., 2011; Bordes *et al*., 2011; Frampton *et al*., 2012; McKenzie *et al*., 2012; Sharp *et al*., 2012).

Recent studies have elucidated the role of some *M. smegmatis* and *M. tuberculosis* TA families. For example, studies on the *M. smegmatis* VapBC locus have implicated VapC as playing a role in carbon transport and metabolism, since VapC expression affects genes involved in glycerol utilization (McKenzie *et al*., 2012). Furthermore, it has been shown that a *M. smegmatis* strain which had all of its TA systems deleted displayed a growth defect in complex medium (Frampton *et al*., 2012).

Less is known about the function of *M. tuberculosis* TA loci, although several have been shown to be induced during stress conditions such as hypoxia or growth in macrophages (Korch *et al*., 2009; Ramage *et al*., 2009; Zhu *et al*., 2010). Singh and colleagues showed that overexpression of RelE toxins in *M. tuberculosis* leads to increased survival during antibiotic treatment (Singh *et al*., 2010), and multiple TAS were found to be upregulated in *M. tuberculosis* selected as drug-tolerant persisters (Keren *et al*., 2011). Although deletion of single RelE genes did not impair survival of the bacteria in a murine infection model (Singh *et al*., 2010), a significant role for TA systems during *M. tuberculosis* infection cannot be ruled out, given the abundance of TA systems and their potential for redundancy.

We were interested in the function of the *M. tuberculosis* HigBA locus, which is one of the few TAS without additional homologues in *M. tuberculosis* (Ramage *et al*., 2009). To elucidate the role of HigB toxin, we constructed a tetracycline-inducible expression plasmid and showed that expression of HigB inhibited growth of *M. tuberculosis*. This is in accord with previous studies where expression of *M. tuberculosis* HigB inhibited growth in *M. marinum* and *M. bovis* BCG (Bordes *et al*., 2011). We also observed a significant loss of viability following induction of HigB, leaving only a subpopulation with the potential to acquire a persister phenotype. Growth arrest and loss of viability were contingent on deletion of the endogenous copy of the Rv1955–Rv1957 operon, indicating that – as in *E. coli*, *V. cholerae* and *P. vulgaris* (Budde *et al*., 2007; Gupta, 2009; Christensen-Dalsgaard *et al*., 2010) – HigA antitoxin is able to counteract HigB toxicity in *M. tuberculosis*.

HigB toxins from other bacteria are characterized by endonuclease activity against mRNAs (Christensen-Dalsgaard and Gerdes, 2006; Hurley and Woychik, 2009; Christensen-Dalsgaard *et al*., 2010). In spite of the marked growth arrest and loss of viability phenotypes, transcriptional profiling 24 h after induction of HigB identified significant changes in the abundance of only a restricted subset of genes. We observed a clear downregulation of genes regulated by the IdeR and Zur repressors involved in regulation of iron and zinc homeostasis in mycobacteria (Rodriguez *et al*., 2002; Maciag *et al*., 2007). This could reflect degradation of these mRNAs by HigB endonuclease activity, or a downstream regulatory consequence of toxin-induced increase in the intracellular availability of iron and zinc. Induction of HigB expression in media containing varying metal ion concentrations, by supplementation of media with additional iron or zinc (Serafini *et al*., 2009), or growth in the presence of the zinc chelator TPEN (Grover and Sharma, 2006) did not uncover any obvious link between the availability of metal ions and toxin-mediated changes in growth and viability (Fig. S2).

Given recent interest in the role of *trans*-translation in drug sensitivity and persistence of *M. tuberculosis* (Shi *et al*., 2011), we were particularly interested in testing whether *M. tuberculosis* HigB exhibited activity against tmRNA. Our results show that *M. tuberculosis* HigB expression leads to tmRNA cleavage, generating 5′ and 3′ fragments that are subsequently degraded. Similarly, expression of *M. tuberculosis* HigB resulted in cleavage of the closely related tmRNA homologues in *M. smegmatis* and *E. coli*. Detailed characterization of the initial fragmentation products shows that tmRNA is cleaved within the mRNA-like 12-codon coding sequence. This is in accordance with the general view that HigB toxins (and also *E. coli* RelE toxin) are translation-dependent and only cleave RNA transcripts during the process of translation (Christensen *et al*., 2003; Christensen-Dalsgaard and Gerdes, 2006; Hurley and Woychik, 2009; Christensen-Dalsgaard *et al*., 2010).

In summary, inhibition of growth following induction of HigB toxin in the absence of its cognate HigA antitoxin had a significant bactericidal effect on *M. tuberculosis*. This differs from the predominantly bacteriostatic effects observed with HigB toxins of *P. vulgaris* and *V. cholerae* (Christensen-Dalsgaard and Gerdes, 2006; Budde *et al*., 2007). Induction of HigB resulted in decreased abundance of IdeR and Zur regulated mRNAs together with site-specific cleavage and subsequent degradation of tmRNA. tmRNA cleavage provides a clear phenotypic marker that may be useful in screening infected tissues for evidence of HigB activation during *M. tuberculosis* infection.

## Experimental procedures

### Bacterial strains and culture conditions

*Escherichia coli* Dh5α was used for plasmid construction and grown at 37°C with shaking at 225 r.p.m. in Luria–Bertani (LB) broth or on Luria–Bertani agar. The *E. coli* HigB overexpression strain, W3110 transformed with pK6-HigB (Bordes *et al*., 2011), was a kind gift from Pierre Genevaux. Ampicillin was used at 100 mg l^−1^, gentamicin at 20 mg l^−1^and kanamycin at 50 mg l^−1^. *M. smegmatis* mc^2^155, *M. tuberculosis* H37Rv (ATCC 25618) and *M. tuberculosis* ΔRv1955–1957 (Fivian-Hughes and Davis, 2010) were grown at 37°C in modified Dubos medium (Difco) or on Difco Middlebrook 7H11 agar (Becton Dickinson) both supplemented with 4% Dubos medium albumin (Difco) and 0.5% or 0.2% w/v glycerol respectively. *M. smegmatis* liquid cultures were grown shaking at 100 r.p.m. and *M. tuberculosis* liquid cultures were grown in a roller incubator at 2 r.p.m. Gentamicin was used at 10 mg l^−1^ and kanamycin at 20 mg l^−1^, when required. All procedures with *M. tuberculosis* were carried out under Containment level 3 conditions.

### Plasmid construction and oligonucleotides

For tetracycline-inducible gene expression, we constructed a novel vector, pTETR3 (available from Addgene, http://www.addgene.org), which combines the tetracycline-inducible promoter P_myc1tetO_ from pSE100 (Ehrt *et al*., 2005), and a codon optimized TetR repressor linked to the P_imyc_ promoter from pTE-10 M-OX (Klotzsche *et al*., 2009). The vector was based on pKP186, a kanamycin-resistant pMV306 derivative that does not contain integrase (Rickman *et al*., 2005). For electroporations, integrase was supplied by pBS-Int, an ampicillin-resistant mycobacterial suicide vector containing L5 integrase (Springer *et al*., 2001). To amplify HigB (Rv1955) from *M. tuberculosis* genomic DNA, primers Rv1955T F and Rv1955T R were used. The PCR product was cloned into pTetR3 as a PacI/EcoRI fragment. The resulting plasmid was named pDS227 and verified by DNA sequencing. Primers and oligonucleotides used in this study are listed in Table S2. Plasmids were transformed into mycobacteria by electroporation as described previously (Goude and Parish, 2009).

### Assaying the effect of HigB overexpression on growth

To assess the effect of toxin overexpression during aerobic growth, liquid cultures were grown to an OD_580_ of 0.3–0.6 (unless otherwise stated) before addition of inducer. Growth was monitored by measuring optical densities. For cfu determination, 10-fold serial dilution series were prepared and 30 μl of each dilution were spread on three-sector plates (BD-Falcon). Experiments were performed on three independent biological replicates. To induce *higB* expression in mycobacteria, 300 ng ml^−1^ anhydrotetracycline (ATc) was added.

*Escherichia coli* transformed with pPK6-HigB was grown with 0.4% glucose. Glucose was removed by washing before *higB* expression was induced by the addition of 0.5% l-arabinose.

### RNA preparation and qRT-PCR analysis

*Mycobacterium tuberculosis* cultures were grown to mid-exponential phase (unless otherwise stated), and RNA was prepared using a FastRNA pro blue kit (Qbiogene). Contaminating DNA was removed using a TURBO DNA-free kit (Ambion), and 1 μg of RNA was converted to cDNA using SuperScript III reverse transcriptase (RT) (Invitrogen) with 250 ng of random primers (Invitrogen). Quantitative RT-PCR (qRT-PCR) was carried out on a 7500 fast real-time PCR system (Applied Biosystems) using fast SYBR green master mix (Applied Biosystems). RNA without RT (RT−) was analysed alongside cDNA (RT+). Standard curves were performed for each gene analysed, and the quantities of cDNA within the samples were calculated from cycle threshold values. Data were averaged, adjusted for chromosomal DNA contamination (RT+ minus RT−), and normalized to corresponding *sigA* or 16S values.

### Northern blots

Northern blots were carried out as described previously (Arnvig and Young, 2009). Unless otherwise stated 5 μg of total RNA were loaded on a 6% denaturing acrylamide gel. Gels were electrophoresed at 12 W for 2 h and electroblotted onto BrightStar-Plus membrane (Ambion). After air-drying, RNA was cross-linked to the membrane by UV irradiation. Membranes were stained in 0.3 M sodium acetate containing 0.03% methylene blue and incubated overnight with labelled probes in ULTRAhyb (Ambion). After washing, membranes were exposed to phosphorimaging and changes in RNA expression were determined by densitometer-scanning of Northern blots. Transcript sizes were compared with RNA marker low from Abnova (20–500 nucleotides).

### RNAseq

RNA was isolated as previously described (Arnvig *et al*., 2011) and treated with Turbo DNase (Ambion) until DNA free. The quality of RNA was assessed using a Nanodrop (ND-1000, Labtech) and Agilent RNA chip (2100 Bioanalyser). Total RNA (2–3 μg) was fragmented (Ambion Cat # AM8740) and strand-specific cDNA libraries were constructed using the Illumina directional mRNA-Seq protocol (Part # 15018460 Rev. A) but with exclusion of poly-A tail and size selection to capture all RNA species. Briefly, this protocol ligates the Illumina v1.5 small RNA 3′ adapter followed by a 5′ adapter to preserve strand specificity. Single-end read sequencing was performed on HiSeq 2000 sequencer. Quality of the Illumina produced fastq files was assessed and good quality reads were mapped to the reference sequence of *M. tuberculosis* H37Rv (EMBL accession code AL123456) as single end data using BWA (Li and Durbin, 2009). Genome coverage, defined as number of reads mapped per base of H37Rv genome, was calculated using BEDTools (Quinlan and Hall, 2010). RPKM values (reads per kilobase per million reads) were calculated using only sequence reads that mapped to annotated features unambiguously and on the correct strand. For whole transcriptome differential expression calling, genome coverage of reads mapping to genes, antisense and ncRNAs were used for statistical testing using DESeq (Anders and Huber, 2010). Differentially expressed genes were considered when fold changes were greater or equal than twofold and the corresponding adjusted *P*-value (*P*adj) was less than 0.05. RNA-seq data have been deposited in ArrayExpress under Accession No. E-MTAB-1667.

*3′ RACE*. To map the 3′ sites of *ssrA* transcripts, 3′ RACE (rapid amplification of cDNA ends) was performed using a Gene Racer kit (Invitrogen) according to the manufacturer's instruction. Five micrograms of DNA-free RNA was poly-A tailed using *E. coli* poly-A Polymerase (Ambion). cDNA was synthesized using SuperScript III and oligo d(T) primer. cDNA was used as a template for PCR with Phusion HF MasterMix (Finnzymes) using GR3′ (linker specific) and a gene-specific forward primer (TaqSsrAb F). PCR products were separated on a 2% agarose gel and bands of interest cut out, cloned into pCR-II TOPO (Invitrogen) and sequenced. 3′ ends of transcripts were identified as the junction with the poly-A tail.
