# Supplementary material for: Induced ectopic expression of HigB toxin in Mycobacterium tuberculosis results in growth inhibition, reduced abundance of a subset of mRNAs and cleavage of tmRNA
Source: Mol Microbiol. 2013 Aug 23;90(1):195–207. doi: 10.1111/mmi.12358 (PMC3912914; doi:10.1111/mmi.12358)
Supplement: Supplementary file 1 [file mmi0090-0195-sd1.pdf]

Supplementary Material to:

Induced ectopic expression of HigB toxin in *Mycobacterium tuberculosis* results in growth inhibition, reduced abundance of a subset of mRNAs and cleavage of tmRNA

Dorothée L. Schuessler, Teresa Cortes, Amanda S. Fivian-Hughes, Kathryn E. A. Lougheed, Evelyn Harvey, Roger S. Buxton*, Elaine O. Davis and Douglas B. Young

Division of Mycobacterial Research, MRC National Institute for Medical Research, The Ridgeway, Mill Hill, London, NW7 1AA, United Kingdom

*Corresponding author

For correspondence

R.S. Buxton

Email: rbuxton@nimr.mrc.ac.uk

Tel: +44 (0)20 8816 2225

D.L. Schuessler

Email: d.l.schuessler.03@cantab.net

D.B. Young

Email: dyoung@nimr.mrc.ac.uk

Tel: +44 (0)20 8816 2657

Table S1. RNA sequencing analysis.

Table S2. Oligonucleotides used in this study.

Fig. S1. Quantitative RT-PCR analysis of *higB* expression in ΔTAC strains.

Fig. S2. HigB expression with varying metal ion concentrations.

Table S1. RNA sequencing analysis of M. tuberculosis H37Rv overexpressing HigB and the empty vector control strain. Number of reads mapped and RPKM (reads per kilobase per million) values are indicated for each of the annotated H37Rv genes.
(Essentiality is taken from Sassett et al., 2003).

	Vector Control Strain		HigB expressing strain		Essentiality
Rv Number	Sense Reads	RPKM	Sense Reads	RPKM	
Rv0001	209	2.95	187	2.92	Essential
Rv0002	431	7.66	500	9.86	Non Essential
Rv0003	432	8.02	395	8.13	Non Essential
Rv0004	105	4.01	86	3.64	
Rv0005	3915	41.49	2738	32.17	Essential
Rv0006	4203	35.88	3023	28.61	Non Essential
Rv0007	931	21.88	744	19.39	Non Essential
Rv0008c	58	2.85	67	3.65	Non Essential
Rv0009	529	20.74	1136	49.37	Non Essential
Rv0010c	32	1.62	34	1.91	Non Essential
Rv0011c	56	4.28	52	4.41	Non Essential
Rv0012	170	4.63	140	4.23	Non Essential
Rv0013	43	1.32	39	1.33	Non Essential
Rv0014c	233	2.66	222	2.81	Essential
Rv0015c	317	5.26	305	5.61	Essential
Rv0016c	606	8.83	545	8.80	Non Essential
Rv0017c	402	6.13	335	5.66	Non Essential
Rv0018c	363	5.05	324	5.00	Non Essential
Rv0019c	432	19.87	363	18.51	Non Essential
Rv0020c	1894	25.70	2000	30.09	Non Essential
Rv0021c	42	0.93	54	1.33	Non Essential
Rv0022c	6	0.31	3	0.17	Non Essential
Rv0023	72	2.01	71	2.20	Non Essential
Rv0024	93	2.36	82	2.31	Non Essential
Rv0025	98	5.82	125	8.22	Non Essential
Rv0026	158	2.52	129	2.28	Non Essential
Rv0027	5	0.34	8	0.60	Non Essential
Rv0028	20	1.41	11	0.86	Non Essential
Rv0029	449	8.79	411	8.92	Non Essential
Rv0030	69	4.51	74	5.36	Non Essential
Rv0031	0	0.00	0	0.00	Non Essential
Rv0032	155	1.44	143	1.47	Non Essential
Rv0033	35	2.86	37	3.35	Non Essential
Rv0034	27	1.47	20	1.21	Non Essential
Rv0035	60	0.76	74	1.04	Non Essential
Rv0036c	225	6.25	220	6.78	Non Essential
Rv0037c	162	2.63	151	2.71	Non Essential
Rv0038	114	4.03	108	4.23	Non Essential
Rv0039c	83	5.14	70	4.80	Non Essential
Rv0040c	468	10.79	563	14.39	Non Essential
Rv0041	767	5.66	827	6.77	Essential
Rv0042c	912	31.29	1026	39.03	Non Essential

Rv0043c	148	4.33	154	5.00	Non Essential
Rv0044c	187	5.06	213	6.39	Non Essential
Rv0045c	217	5.20	246	6.54	Non Essential
Rv0046c	162	3.15	167	3.61	Non Essential
Rv0047c	146	5.79	119	5.23	Non Essential
Rv0048c	45	1.11	44	1.21	Non Essential
Rv0049	186	9.67	221	12.74	Non Essential
Rv0050	1355	14.30	1346	15.74	Non Essential
Rv0051	182	2.32	197	2.79	Non Essential
Rv0052	84	3.20	57	2.41	Non Essential
Rv0053	1981	146.73	3583	294.25	Non Essential
Rv0054	456	19.83	720	34.71	Non Essential
Rv0055	939	79.41	1611	151.05	Non Essential
Rv0056	795	37.29	1361	70.77	Non Essential
Rv0057	628	25.89	679	31.04	Non Essential
Rv0058	2204	18.04	2496	22.65	Essential
Rv0059	201	6.24	181	6.23	Non Essential
Rv0060	369	7.49	388	8.73	Essential
Rv0061c	84	5.34	81	5.71	Non Essential
Rv0062	50	0.94	127	2.65	Non Essential
Rv0063	169	2.52	172	2.85	Non Essential
Rv0064	543	3.97	512	4.15	Non Essential
Rv0064A	38	3.42	22	2.19	
Rv0065	19	1.02	10	0.59	Non Essential
Rv0066c	1015	9.75	1043	11.10	Non Essential
Rv0067c	18	0.68	13	0.54	Non Essential
Rv0068	43	1.01	47	1.23	Non Essential
Rv0069c	125	1.94	168	2.89	Non Essential
Rv0070c	107	1.80	132	2.46	Non Essential
Rv0071	23	0.70	35	1.18	Non Essential
Rv0072	670	13.72	617	14.01	Non Essential
Rv0073	697	15.09	608	14.60	Non Essential
Rv0074	571	9.93	464	8.95	Non Essential
Rv0075	191	3.50	137	2.78	Non Essential
Rv0076c	8	0.44	14	0.86	Non Essential
Rv0077c	10	0.26	8	0.23	Non Essential
Rv0078	39	1.38	57	2.24	Non Essential
Rv0078A	124	4.49	130	5.22	Non Essential
Rv0079	230	6.02	394	11.43	Non Essential
Rv0080	21	0.98	44	2.29	Non Essential
Rv0081	104	6.49	73	5.05	Non Essential
Rv0082	121	5.43	87	4.33	Non Essential
Rv0083	217	2.43	159	1.97	Non Essential
Rv0084	225	5.09	162	4.06	Non Essential
Rv0085	135	4.38	66	2.37	Essential
Rv0086	311	4.56	143	2.32	Essential
Rv0087	462	6.71	236	3.80	Non Essential
Rv0088	429	13.67	335	11.84	Non Essential
Rv0089	49	1.77	25	1.00	Non Essential
Rv0090	23	0.64	19	0.59	Non Essential

Rv0091	42	1.18	50	1.55	Non Essential
Rv0092	250	2.35	258	2.69	Non Essential
Rv0093c	89	2.25	87	2.44	Non Essential
Rv0094c	14	0.32	13	0.32	Non Essential
Rv0095c	11	0.58	21	1.22	Non Essential
Rv0096	2	0.03	9	0.15	Non Essential
Rv0097	43	1.06	43	1.18	Non Essential
Rv0098	13	0.51	14	0.61	Essential
Rv0099	130	1.72	206	3.02	Essential
Rv0100	30	2.73	47	4.74	Essential
Rv0101	319	0.91	430	1.36	Essential
Rv0102	445	4.82	466	5.59	Essential
Rv0103c	490	4.66	525	5.54	Non Essential
Rv0104	136	1.93	94	1.48	Non Essential
Rv0105c	10	0.76	0	0.00	Non Essential
Rv0106	412	7.40	85	1.69	Non Essential
Rv0107c	1040	4.56	764	3.71	Non Essential
Rv0108c	291	29.91	220	25.07	Non Essential
Rv0109	184	2.65	161	2.57	Non Essential
Rv0110	51	1.46	68	2.16	Non Essential
Rv0111	490	5.12	509	5.89	Non Essential
Rv0112	124	2.79	126	3.14	Essential
Rv0113	52	1.89	30	1.21	Non Essential
Rv0114	141	5.30	132	5.50	Non Essential
Rv0115	51	0.94	55	1.13	Non Essential
Rv0116c	63	1.79	53	1.67	Non Essential
Rv0117	16	0.36	25	0.63	Non Essential
Rv0118c	356	4.37	198	2.70	Essential
Rv0119	84	1.14	70	1.06	Non Essential
Rv0120c	328	3.29	231	2.57	Non Essential
Rv0121c	18	0.89	15	0.82	Non Essential
Rv0122	22	1.28	34	2.20	Non Essential
Rv0123	23	1.34	19	1.23	Non Essential
Rv0124	50	0.73	31	0.50	Non Essential
Rv0125	389	7.83	374	8.35	Non Essential
Rv0126	707	8.41	656	8.66	Non Essential
Rv0127	565	8.88	500	8.71	Essential
Rv0128	78	2.15	96	2.93	Non Essential
Rv0129c	1249	26.25	1386	32.30	Non Essential
Rv0130	18	0.85	22	1.15	Non Essential
Rv0131c	108	1.73	89	1.58	Non Essential
Rv0132c	12	0.24	16	0.35	Non Essential
Rv0133	75	2.66	68	2.68	Non Essential
Rv0134	309	7.36	334	8.82	Non Essential
Rv0135c	214	7.60	167	6.57	Non Essential
Rv0136	82	1.33	80	1.44	Non Essential
Rv0137c	61	2.39	70	3.04	Non Essential
Rv0138	69	2.95	52	2.46	Non Essential
Rv0139	133	2.80	133	3.10	Non Essential
Rv0140	44	2.49	77	4.83	Non Essential

Rv0141c	245	12.84	225	13.07	Non Essential
Rv0142	53	1.23	46	1.18	Non Essential
Rv0143c	530	7.70	515	8.30	Non Essential
Rv0144	999	25.49	666	18.84	Non Essential
Rv0145	1785	40.23	1351	33.76	Non Essential
Rv0146	341	7.86	217	5.55	Non Essential
Rv0147	675	9.54	551	8.63	Non Essential
Rv0148	563	14.06	434	12.02	Non Essential
Rv0149	366	8.12	289	7.11	Non Essential
Rv0150c	5	0.37	2	0.17	Non Essential
Rv0151c	23	0.28	13	0.18	Non Essential
Rv0152c	34	0.46	28	0.42	Non Essential
Rv0153c	72	1.86	76	2.18	Non Essential
Rv0154c	163	2.89	165	3.24	Non Essential
Rv0155	352	6.87	425	9.20	Non Essential
Rv0156	25	1.62	25	1.79	Non Essential
Rv0157	1985	29.88	2517	42.01	Non Essential
Rv0157A	33	5.54	29	5.40	
Rv0158	104	3.47	74	2.74	Non Essential
Rv0159c	55	0.84	48	0.81	Non Essential
Rv0160c	3	0.04	4	0.06	Non Essential
Rv0161	39	0.62	48	0.85	Non Essential
Rv0162c	185	3.45	193	3.99	Non Essential
Rv0163	37	1.75	52	2.72	Non Essential
Rv0164	190	8.42	167	8.20	Non Essential
Rv0165c	54	1.73	77	2.73	Non Essential
Rv0166	999	12.90	810	11.59	Non Essential
Rv0167	397	10.70	345	10.31	Non Essential
Rv0168	585	14.46	608	16.66	Non Essential
Rv0169	1231	19.39	1282	22.38	Essential
Rv0170	1207	24.93	1080	24.73	Essential
Rv0171	926	12.86	858	13.21	Essential
Rv0172	588	7.93	554	8.29	Non Essential
Rv0173	672	12.32	645	13.11	Essential
Rv0174	5206	72.29	4936	75.99	Non Essential
Rv0175	3103	103.98	2990	111.09	Essential
Rv0176	453	10.05	424	10.43	Essential
Rv0177	2742	106.32	2550	109.62	Non Essential
Rv0178	413	12.09	398	12.91	Non Essential
Rv0179c	700	13.56	770	16.54	Non Essential
Rv0180c	176	2.78	195	3.42	Non Essential
Rv0181c	199	5.82	168	5.45	Non Essential
Rv0182c	208	4.02	237	5.08	Non Essential
Rv0183	156	3.99	171	4.85	Non Essential
Rv0184	214	6.14	228	7.25	Non Essential
Rv0185	222	9.37	224	10.48	Non Essential
Rv0186	618	6.40	539	6.19	Non Essential
Rv0186A	851	113.54	1453	214.93	
Rv0187	117	3.80	97	3.49	Non Essential
Rv0188	289	14.40	253	13.98	Non Essential

Rv0189c	388	4.83	366	5.05	Essential
Rv0190	398	29.48	492	40.40	Non Essential
Rv0191	211	3.65	175	3.36	Non Essential
Rv0192	440	8.59	391	8.47	Non Essential
Rv0192A	169	12.02	150	11.83	Non Essential
Rv0193c	28	0.33	25	0.32	Non Essential
Rv0194	20	0.12	22	0.15	Non Essential
Rv0195	2	0.07	1	0.04	Non Essential
Rv0196	41	1.51	121	4.93	Non Essential
Rv0197	110	1.03	355	3.70	Non Essential
Rv0198c	2060	22.22	1953	23.36	Non Essential
Rv0199	160	5.22	196	7.08	Essential
Rv0200	59	1.84	64	2.21	Non Essential
Rv0201c	283	12.09	215	10.18	Non Essential
Rv0202c	928	6.87	694	5.70	Non Essential
Rv0203	58	3.04	122	7.09	Non Essential
Rv0204c	213	3.70	237	4.56	Essential
Rv0205	73	1.42	78	1.68	Essential
Rv0206c	2534	19.21	2087	17.54	Non Essential
Rv0207c	759	22.40	727	23.78	Non Essential
Rv0208c	191	5.19	148	4.46	Essential
Rv0209	141	2.79	170	3.73	Non Essential
Rv0210	271	3.94	272	4.38	Non Essential
Rv0211	1366	16.12	1151	15.06	Non Essential
Rv0212c	56	1.24	56	1.37	Non Essential
Rv0213c	33	0.54	60	1.09	Non Essential
Rv0214	43	0.57	46	0.68	Non Essential
Rv0215c	155	3.10	186	4.13	Non Essential
Rv0216	47	1.00	57	1.34	Essential
Rv0217c	61	1.44	67	1.76	Non Essential
Rv0218	43	0.70	32	0.57	Essential
Rv0219	22	0.86	14	0.61	Non Essential
Rv0220	473	8.39	442	8.69	Non Essential
Rv0221	272	4.15	203	3.43	Non Essential
Rv0222	143	3.90	127	3.84	Non Essential
Rv0223c	96	1.41	126	2.05	Non Essential
Rv0224c	23	0.65	23	0.72	Essential
Rv0225	373	6.94	386	7.97	Non Essential
Rv0226c	101	1.25	124	1.71	Essential
Rv0227c	567	9.63	675	12.71	Essential
Rv0228	52	0.91	64	1.25	Essential
Rv0229c	60	1.90	70	2.45	Non Essential
Rv0230c	82	1.80	113	2.75	Non Essential
Rv0231	94	1.18	97	1.35	Non Essential
Rv0232	49	1.53	39	1.35	Non Essential
Rv0233	582	13.24	523	13.19	Non Essential
Rv0234c	234	3.27	281	4.36	Non Essential
Rv0235c	57	0.85	54	0.89	Non Essential
Rv0236c	465	2.38	438	2.48	Essential
Rv0236A	132	16.39	185	25.47	Non Essential

Rv0237	218	4.02	301	6.15	Non Essential
Rv0238	295	10.32	256	9.93	Non Essential
Rv0239	1014	93.48	865	88.41	Non Essential
Rv0240	75	3.69	69	3.76	Non Essential
Rv0241c	336	8.57	322	9.11	Non Essential
Rv0242c	1132	17.83	1015	17.72	Non Essential
Rv0243	3548	57.65	3301	59.47	Non Essential
Rv0244c	108	1.26	109	1.41	Non Essential
Rv0245	81	3.57	80	3.90	Non Essential
Rv0246	234	3.84	281	5.11	Non Essential
Rv0247c	366	10.54	458	14.62	Non Essential
Rv0248c	1797	19.90	1990	24.43	Non Essential
Rv0249c	279	7.30	350	10.15	Essential
Rv0250c	314	23.02	397	32.27	Non Essential
Rv0251c	207	9.28	138	6.86	Non Essential
Rv0252	152	1.27	137	1.27	Non Essential
Rv0253	28	1.69	34	2.27	Non Essential
Rv0254c	45	1.84	58	2.64	Non Essential
Rv0255c	205	2.97	198	3.18	Non Essential
Rv0256c	220	2.83	241	3.44	Non Essential
Rv0257	58	3.33	44	2.80	Non Essential
Rv0258c	9	0.42	9	0.47	Non Essential
Rv0259c	3	0.09	9	0.29	Non Essential
Rv0260c	1	0.02	1	0.02	Non Essential
Rv0261c	6	0.09	9	0.15	Non Essential
Rv0262c	51	2.01	55	2.40	Non Essential
Rv0263c	71	1.69	73	1.93	Non Essential
Rv0264c	40	1.36	36	1.36	Non Essential
Rv0265c	180	3.90	167	4.01	Non Essential
Rv0266c	233	1.38	231	1.52	Non Essential
Rv0267	7	0.11	10	0.17	Non Essential
Rv0268c	743	31.36	760	35.56	Non Essential
Rv0269c	101	1.82	101	2.02	Non Essential
Rv0270	1660	21.20	1365	19.33	Non Essential
Rv0271c	265	2.59	191	2.07	Non Essential
Rv0272c	132	2.50	113	2.38	Non Essential
Rv0273c	47	1.63	50	1.92	Non Essential
Rv0274	207	7.65	169	6.93	Non Essential
Rv0275c	160	4.74	128	4.20	Non Essential
Rv0276	879	20.52	733	18.97	Non Essential
Rv0277c	81	4.07	49	2.73	Non Essential
Rv0277A	1487	124.29	1186	109.90	
Rv0278c	138	1.03	123	1.02	Non Essential
Rv0279c	334	2.85	317	3.00	Non Essential
Rv0280	4532	60.47	1108	16.39	Non Essential
Rv0281	852	20.16	310	8.13	Non Essential
Rv0282	16723	189.56	6654	83.63	Essential
Rv0283	9971	132.54	3769	55.55	Essential
Rv0284	10731	57.74	4044	24.13	Essential
Rv0285	1269	88.50	484	37.42	Essential

Rv0286	9845	137.23	3910	60.43	Essential
Rv0287	4067	298.16	1437	116.80	Non Essential
Rv0288	7219	534.72	2401	197.18	Non Essential
Rv0289	3986	96.53	1338	35.93	Essential
Rv0290	5091	77.12	1566	26.30	Essential
Rv0291	4031	62.52	1257	21.61	Essential
Rv0292	2406	51.94	800	19.15	Essential
Rv0293c	373	6.67	411	8.14	Non Essential
Rv0294	14	0.38	16	0.49	Non Essential
Rv0295c	72	1.93	76	2.25	Non Essential
Rv0296c	53	0.81	74	1.26	Non Essential
Rv0297	662	8.01	522	7.00	Non Essential
Rv0298	2079	196.73	1613	169.23	Non Essential
Rv0299	200	14.23	171	13.49	Non Essential
Rv0300	199	19.34	168	18.10	Non Essential
Rv0301	524	26.48	365	20.45	Non Essential
Rv0302	81	2.75	54	2.03	Non Essential
Rv0303	60	1.42	28	0.73	Non Essential
Rv0304c	204	0.66	173	0.62	Non Essential
Rv0305c	385	2.86	299	2.46	Non Essential
Rv0306	5	0.16	8	0.28	Non Essential
Rv0307c	34	1.52	22	1.09	Non Essential
Rv0308	267	8.01	430	14.30	Non Essential
Rv0309	696	22.79	824	29.91	Non Essential
Rv0310c	11	0.48	18	0.87	Non Essential
Rv0311	52	0.91	50	0.97	Essential
Rv0312	244	2.81	281	3.59	Essential
Rv0313	587	32.67	580	35.79	Non Essential
Rv0314c	195	6.33	208	7.48	Non Essential
Rv0315	456	11.08	584	15.73	Non Essential
Rv0316	32	1.12	29	1.12	Non Essential
Rv0317c	348	9.71	310	9.59	Non Essential
Rv0318c	19	0.51	30	0.90	Non Essential
Rv0319	46	1.48	47	1.68	Non Essential
Rv0320	39	1.27	30	1.08	Non Essential
Rv0321	29	1.09	17	0.71	Non Essential
Rv0322	23	0.37	29	0.52	Non Essential
Rv0323c	18	0.58	19	0.67	Non Essential
Rv0324	265	8.37	231	8.09	Non Essential
Rv0325	23	2.21	12	1.28	Non Essential
Rv0326	9	0.42	7	0.37	Essential
Rv0327c	6	0.10	3	0.05	Non Essential
Rv0328	157	5.60	116	4.59	Non Essential
Rv0329c	2	0.07	8	0.30	Non Essential
Rv0330c	16	0.46	13	0.42	Non Essential
Rv0331	126	2.32	106	2.17	Non Essential
Rv0332	158	4.32	133	4.04	Non Essential
Rv0333	63	3.62	73	4.65	Non Essential
Rv0334	99	2.46	80	2.20	Essential
Rv0335c	3	0.13	3	0.14	Essential

Rv0336	319	4.53	245	3.86	Non Essential
Rv0337c	95	1.58	183	3.38	Essential
Rv0338c	469	3.80	1129	10.15	Essential
Rv0339c	213	1.83	177	1.69	Non Essential
Rv0340	67	2.67	58	2.56	Non Essential
Rv0341	1950	29.11	1236	20.46	Non Essential
Rv0342	1169	13.06	527	6.53	Non Essential
Rv0343	165	2.39	108	1.74	Non Essential
Rv0344c	5	0.19	12	0.51	Non Essential
Rv0345	85	4.45	142	8.25	Non Essential
Rv0346c	638	9.37	648	10.55	Non Essential
Rv0347	196	4.27	278	6.71	Essential
Rv0348	201	6.61	217	7.91	Non Essential
Rv0349	29	0.95	22	0.80	Non Essential
Rv0350	4992	57.13	5417	68.73	Essential
Rv0351	4930	149.79	4639	156.27	Essential
Rv0352	2518	45.57	2328	46.71	Essential
Rv0353	581	32.84	556	34.85	Non Essential
Rv0354c	7	0.35	4	0.22	Non Essential
Rv0355c	425	0.92	391	0.94	Non Essential
Rv0356c	59	1.97	66	2.44	Non Essential
Rv0357c	116	1.92	127	2.33	Essential
Rv0358	30	1.00	21	0.77	Non Essential
Rv0359	41	1.13	38	1.16	Non Essential
Rv0360c	79	3.88	127	6.92	Non Essential
Rv0361	68	1.77	55	1.58	Non Essential
Rv0362	45	0.70	32	0.55	Non Essential
Rv0363c	880	18.28	972	22.39	Non Essential
Rv0364	147	4.62	169	5.89	Non Essential
Rv0365c	167	3.17	160	3.37	Non Essential
Rv0366c	61	2.21	49	1.97	Non Essential
Rv0367c	72	3.98	51	3.12	Non Essential
Rv0368c	8	0.14	4	0.08	Non Essential
Rv0369c	22	0.92	16	0.74	Non Essential
Rv0370c	64	1.53	40	1.06	Non Essential
Rv0371c	4	0.14	1	0.04	Non Essential
Rv0372c	10	0.28	9	0.28	Essential
Rv0373c	78	0.70	70	0.69	Non Essential
Rv0374c	5	0.22	7	0.35	Non Essential
Rv0375c	11	0.27	13	0.36	Non Essential
Rv0376c	38	0.71	57	1.19	Non Essential
Rv0377	33	0.73	25	0.62	Non Essential
Rv0378	1	0.10	5	0.54	Non Essential
Rv0379	191	19.08	231	25.59	Non Essential
Rv0380c	39	1.52	29	1.25	Non Essential
Rv0381c	69	1.63	59	1.55	Non Essential
Rv0382c	648	25.82	518	22.89	Non Essential
Rv0383c	1392	35.01	1404	39.15	Essential
Rv0384c	823	6.94	647	6.05	Essential
Rv0385	20	0.37	19	0.39	Non Essential

Rv0386	195	1.29	190	1.39	Non Essential
Rv0387c	14	0.41	26	0.84	Non Essential
Rv0388c	20	0.79	15	0.66	Non Essential
Rv0389	318	5.43	297	5.62	Non Essential
Rv0390	160	8.14	194	10.95	Non Essential
Rv0391	141	2.48	154	3.01	Non Essential
Rv0392c	140	2.13	124	2.09	Non Essential
Rv0393	0	0.00	5	0.09	Non Essential
Rv0394c	385	11.50	623	20.64	Non Essential
Rv0395	3	0.16	0	0.00	Non Essential
Rv0396	4	0.22	7	0.43	Non Essential
Rv0397	3	0.18	0	0.00	Non Essential
Rv0398c	34	1.14	40	1.49	Non Essential
Rv0399c	416	7.27	382	7.40	Essential
Rv0400c	257	4.65	276	5.54	Essential
Rv0401	42	2.43	47	3.02	Non Essential
Rv0402c	170	1.27	181	1.50	Non Essential
Rv0403c	4	0.20	6	0.33	Non Essential
Rv0404	184	2.25	185	2.51	Essential
Rv0405	887	4.53	796	4.51	Non Essential
Rv0406c	28	0.74	33	0.96	Non Essential
Rv0407	172	3.66	207	4.88	Non Essential
Rv0408	310	3.21	252	2.90	Non Essential
Rv0409	337	6.26	263	5.41	Non Essential
Rv0410c	910	8.68	612	6.47	Essential
Rv0411c	425	9.26	303	7.32	Essential
Rv0412c	338	5.50	282	5.09	Essential
Rv0413	106	3.49	110	4.01	Non Essential
Rv0414c	5	0.16	13	0.46	Essential
Rv0415	62	1.30	42	0.98	Essential
Rv0416	16	1.67	11	1.27	Essential
Rv0417	32	0.91	29	0.91	Essential
Rv0418	469	6.71	585	9.28	Non Essential
Rv0419	576	8.27	658	10.48	Non Essential
Rv0420c	22	1.15	23	1.34	Non Essential
Rv0421c	68	2.32	72	2.73	Non Essential
Rv0422c	233	6.28	177	5.29	Essential
Rv0423c	1062	13.88	871	12.63	Essential
Rv0424c	794	62.02	688	59.58	Non Essential
Rv0425c	2484	11.55	1856	9.57	Essential
Rv0426c	739	35.83	657	35.32	Non Essential
Rv0427c	138	3.39	162	4.41	Essential
Rv0428c	59	1.40	65	1.70	Non Essential
Rv0429c	42	1.52	49	1.97	Essential
Rv0430	162	11.30	206	15.93	Essential
Rv0431	167	7.26	182	8.77	Non Essential
Rv0432	189	5.62	225	7.42	Non Essential
Rv0433	356	6.77	327	6.89	Non Essential
Rv0434	147	4.84	116	4.23	Non Essential
Rv0435c	274	2.69	209	2.28	Non Essential

Rv0436c	186	4.65	175	4.85	Non Essential
Rv0437c	189	5.84	194	6.65	Non Essential
Rv0438c	72	1.27	104	2.04	Non Essential
Rv0439c	84	1.93	100	2.55	Non Essential
Rv0440	22730	301.02	21309	312.88	Essential
Rv0441c	22	1.10	24	1.34	Non Essential
Rv0442c	212	3.11	173	2.82	Non Essential
Rv0443	164	6.84	207	9.57	Non Essential
Rv0444c	250	7.69	236	8.05	Non Essential
Rv0445c	159	6.07	136	5.75	Non Essential
Rv0446c	173	4.83	233	7.21	Non Essential
Rv0447c	256	4.29	262	4.86	Non Essential
Rv0448c	7	0.23	3	0.11	Non Essential
Rv0449c	227	3.70	110	1.99	Non Essential
Rv0450c	1419	10.50	563	4.62	Essential
Rv0451c	199	10.13	66	3.72	Non Essential
Rv0452	75	2.27	65	2.18	Non Essential
Rv0453	47	0.65	41	0.63	Non Essential
Rv0454	11	0.68	7	0.48	Non Essential
Rv0455c	92	4.43	70	3.74	Non Essential
Rv0456c	14	0.33	8	0.21	Non Essential
Rv0456A	8	0.61	16	1.36	Non Essential
Rv0456B	233	28.93	238	32.76	
Rv0457c	70	0.74	74	0.87	Non Essential
Rv0458	302	4.26	177	2.77	Non Essential
Rv0459	241	10.54	177	8.59	Non Essential
Rv0460	150	13.48	187	18.63	Non Essential
Rv0461	122	5.00	128	5.82	Non Essential
Rv0462	1214	18.71	1465	25.03	Essential
Rv0463	58	4.25	96	7.80	Non Essential
Rv0464c	110	4.13	81	3.37	Non Essential
Rv0465c	196	2.96	161	2.69	Non Essential
Rv0466	220	5.95	290	8.70	Non Essential
Rv0467	134	2.24	77	1.43	Non Essential
Rv0468	625	15.61	625	17.31	Non Essential
Rv0469	260	6.49	214	5.93	Non Essential
Rv0470c	271	6.75	303	8.36	Essential
Rv0470A	39	1.90	21	1.14	Non Essential
Rv0471c	59	2.60	46	2.24	Non Essential
Rv0472c	1507	45.98	1440	48.71	Non Essential
Rv0473	273	4.28	244	4.24	Non Essential
Rv0474	86	4.38	65	3.67	Non Essential
Rv0475	954	34.21	821	32.64	Non Essential
Rv0476	68	5.55	56	5.07	Non Essential
Rv0477	53	2.55	42	2.24	Non Essential
Rv0478	150	4.78	104	3.67	Non Essential
Rv0479c	244	5.01	244	5.56	Essential
Rv0480c	60	1.53	65	1.84	Non Essential
Rv0481c	136	5.58	84	3.82	Non Essential
Rv0482	54	1.05	48	1.03	Non Essential

Rv0483	251	3.98	287	5.04	Non Essential
Rv0484c	52	1.48	68	2.14	Non Essential
Rv0485	471	7.69	418	7.56	Non Essential
Rv0486	964	14.36	808	13.34	Non Essential
Rv0487	630	24.56	493	21.31	Non Essential
Rv0488	27	0.96	19	0.75	Non Essential
Rv0489	670	19.21	802	25.50	Non Essential
Rv0490	1137	19.82	1011	19.54	Essential
Rv0491	1045	32.87	963	33.58	Non Essential
Rv0492c	43	0.49	38	0.48	Non Essential
Rv0492A	14	0.91	8	0.58	Non Essential
Rv0493c	64	1.39	77	1.85	Non Essential
Rv0494	2	0.06	6	0.20	Non Essential
Rv0495c	304	7.34	393	10.52	Non Essential
Rv0496	932	20.30	1117	26.98	Non Essential
Rv0497	283	6.52	361	9.22	Non Essential
Rv0498	194	4.95	187	5.29	Non Essential
Rv0499	207	5.08	225	6.12	Non Essential
Rv0500	147	3.56	124	3.33	Essential
Rv0500A	154	14.02	132	13.32	Non Essential
Rv0500B	189	40.20	239	56.36	Non Essential
Rv0501	760	14.45	785	16.54	Non Essential
Rv0502	563	11.24	379	8.39	Non Essential
Rv0503c	184	4.35	190	4.98	Non Essential
Rv0504c	241	10.35	249	11.86	Non Essential
Rv0505c	202	3.87	210	4.46	Non Essential
Rv0506	83	4.02	98	5.27	Non Essential
Rv0507	342	2.53	309	2.53	Non Essential
Rv0508	70	5.13	46	3.74	Non Essential
Rv0509	1622	24.78	1276	21.61	Essential
Rv0510	1153	26.66	1256	32.20	Essential
Rv0511	1744	22.08	1607	22.55	Essential
Rv0512	1403	30.47	1204	28.99	Non Essential
Rv0513	1001	39.24	784	34.07	Non Essential
Rv0514	108	7.76	97	7.73	Non Essential
Rv0515	306	4.35	259	4.08	Non Essential
Rv0516c	50	2.26	84	4.20	Non Essential
Rv0517	220	3.61	238	4.33	Non Essential
Rv0518	84	2.60	101	3.46	Non Essential
Rv0519c	209	4.98	236	6.23	Non Essential
Rv0520	0	0.00	3	0.20	Non Essential
Rv0521	2	0.14	4	0.31	Non Essential
Rv0522	366	6.03	221	4.04	Non Essential
Rv0523c	15	0.82	18	1.09	Non Essential
Rv0524	135	2.09	260	4.46	Essential
Rv0525	127	4.49	216	8.46	Essential
Rv0526	81	2.68	132	4.84	Essential
Rv0527	270	7.45	345	10.55	Essential
Rv0528	204	2.76	278	4.17	Essential
Rv0529	283	6.24	348	8.51	Essential

Rv0530	253	4.47	259	5.07	Non Essential
Rv0531	116	7.86	104	7.81	Non Essential
Rv0532	230	2.77	208	2.78	Non Essential
Rv0533c	225	4.80	214	5.06	Non Essential
Rv0534c	88	2.15	85	2.31	Non Essential
Rv0535	47	1.27	77	2.31	Non Essential
Rv0536	35	0.72	42	0.96	Non Essential
Rv0537c	240	3.60	219	3.64	Non Essential
Rv0538	332	4.33	313	4.53	Non Essential
Rv0539	77	2.62	65	2.45	Non Essential
Rv0540	15	0.49	9	0.32	Essential
Rv0541c	446	7.10	347	6.13	Essential
Rv0542c	530	10.46	428	9.37	Essential
Rv0543c	87	6.19	68	5.36	Non Essential
Rv0544c	87	6.72	89	7.62	Non Essential
Rv0545c	1451	24.87	1334	25.36	Non Essential
Rv0546c	60	3.34	78	4.81	Non Essential
Rv0547c	81	1.97	119	3.21	Non Essential
Rv0548c	499	11.35	615	15.52	Non Essential
Rv0549c	32	1.66	17	0.98	Non Essential
Rv0550c	33	2.66	51	4.57	Non Essential
Rv0551c	101	1.27	110	1.53	Non Essential
Rv0552	162	2.17	145	2.15	Non Essential
Rv0553	29	0.64	36	0.87	Essential
Rv0554	60	1.64	79	2.39	Non Essential
Rv0555	332	4.29	305	4.37	Essential
Rv0556	356	14.85	333	15.40	Essential
Rv0557	337	6.37	265	5.56	Essential
Rv0558	186	5.68	253	8.56	Essential
Rv0559c	891	56.62	950	66.94	Non Essential
Rv0560c	154	4.56	85	2.79	Non Essential
Rv0561c	112	1.96	114	2.21	Non Essential
Rv0562	798	17.02	818	19.35	Essential
Rv0563	673	16.81	612	16.95	Non Essential
Rv0564c	36	0.75	42	0.98	Non Essential
Rv0565c	124	1.82	145	2.37	Non Essential
Rv0566c	35	1.53	61	2.96	Essential
Rv0567	175	3.69	212	4.95	Non Essential
Rv0568	462	7.00	438	7.36	Non Essential
Rv0569	275	22.21	207	18.53	Non Essential
Rv0570	169	1.75	175	2.01	Non Essential
Rv0571c	149	2.40	181	3.24	Non Essential
Rv0572c	3	0.19	6	0.42	Non Essential
Rv0573c	37	0.57	46	0.79	Non Essential
Rv0574c	35	0.66	29	0.60	Non Essential
Rv0575c	16	0.29	41	0.84	Non Essential
Rv0576	170	2.80	144	2.63	Non Essential
Rv0577	202	5.53	174	5.28	Non Essential
Rv0578c	1872	10.26	1406	8.54	Non Essential
Rv0579	103	2.92	95	2.98	Non Essential

Rv0580c	299	13.08	242	11.74	Non Essential
Rv0581	527	52.65	669	74.11	Non Essential
Rv0582	34	1.79	47	2.75	Non Essential
Rv0583c	57	1.78	89	3.09	Non Essential
Rv0584	76	0.62	62	0.56	Non Essential
Rv0585c	77	0.69	63	0.63	Non Essential
Rv0586	144	4.28	82	2.70	Non Essential
Rv0587	147	3.96	116	3.47	Non Essential
Rv0588	525	12.71	326	8.75	Essential
Rv0589	253	4.48	172	3.37	Non Essential
Rv0590	253	6.57	177	5.10	Non Essential
Rv0590A	34	2.88	14	1.31	Non Essential
Rv0591	25	0.37	34	0.56	Non Essential
Rv0592	10	0.14	20	0.31	Non Essential
Rv0593	21	0.37	11	0.22	Non Essential
Rv0594	72	1.00	44	0.68	Non Essential
Rv0595c	28	1.53	22	1.34	Non Essential
Rv0596c	29	2.42	28	2.59	Non Essential
Rv0597c	296	5.15	260	5.01	Non Essential
Rv0598c	118	6.14	97	5.59	Non Essential
Rv0599c	40	3.64	38	3.83	Non Essential
Rv0600c	2	0.08	1	0.05	Non Essential
Rv0601c	0	0.00	2	0.10	Non Essential
Rv0602c	13	0.37	20	0.63	Non Essential
Rv0603	4	0.28	7	0.54	Non Essential
Rv0604	96	2.17	92	2.31	Non Essential
Rv0605	228	8.06	248	9.71	Non Essential
Rv0606	432	12.49	393	12.60	Non Essential
Rv0607	9	0.50	7	0.43	Essential
Rv0608	473	41.47	510	49.58	Non Essential
Rv0609	354	18.96	377	22.39	Non Essential
Rv0609A	133	12.59	151	15.84	Non Essential
Rv0610c	239	4.44	201	4.14	Non Essential
Rv0611c	151	8.47	152	9.45	Non Essential
Rv0612	26	0.92	27	1.06	Non Essential
Rv0613c	1462	12.23	1823	16.91	Non Essential
Rv0614	14	0.30	24	0.58	Non Essential
Rv0615	297	26.36	300	29.52	Non Essential
Rv0616c	2	0.16	2	0.18	Non Essential
Rv0616A	143	13.53	138	14.48	
Rv0617	103	5.52	108	6.41	Non Essential
Rv0618	43	1.33	40	1.37	Non Essential
Rv0619	0	0.00	5	0.22	Non Essential
Rv0620	2	0.04	0	0.00	Non Essential
Rv0621	33	0.67	49	1.10	Non Essential
Rv0622	59	1.34	52	1.31	Non Essential
Rv0623	38	3.21	34	3.19	Non Essential
Rv0624	41	2.23	28	1.69	Non Essential
Rv0625c	55	1.60	58	1.87	Non Essential
Rv0626	83	6.86	115	10.53	Non Essential

Rv0627	48	2.53	40	2.34	Essential
Rv0628c	221	4.12	240	4.97	Essential
Rv0629c	283	3.52	263	3.63	Non Essential
Rv0630c	214	1.40	212	1.54	Non Essential
Rv0631c	110	0.72	134	0.97	Non Essential
Rv0632c	408	12.61	540	18.50	Non Essential
Rv0633c	383	9.81	373	10.59	Non Essential
Rv0634c	169	5.09	198	6.61	Non Essential
Rv0634A	129	11.04	159	15.09	Non Essential
Rv0634B	50	6.43	85	12.12	Non Essential
Rv0635	1290	58.21	1580	79.05	Essential
Rv0636	902	45.27	1131	62.93	Essential
Rv0637	296	12.72	415	19.77	Non Essential
Rv0638	1981	87.74	1934	94.97	Essential
Rv0639	1571	47.13	1538	51.16	Non Essential
Rv0640	1356	68.05	1367	76.07	Essential
Rv0641	3983	121.01	3980	134.07	Non Essential
Rv0642c	533	12.65	640	16.84	Non Essential
Rv0643c	166	4.05	195	5.27	Essential
Rv0644c	179	4.46	208	5.74	Non Essential
Rv0645c	359	8.97	386	10.69	Non Essential
Rv0646c	122	2.90	147	3.87	Non Essential
Rv0647c	161	2.36	231	3.75	Non Essential
Rv0648	12	0.07	17	0.11	Non Essential
Rv0649	5	0.16	5	0.18	Non Essential
Rv0650	2	0.05	4	0.10	Non Essential
Rv0651	119	4.77	169	7.51	Essential
Rv0652	199	10.90	330	20.05	Essential
Rv0653c	26	0.80	20	0.69	Non Essential
Rv0654	222	3.17	202	3.20	Non Essential
Rv0655	1788	35.60	1576	34.79	Essential
Rv0656c	19	1.07	18	1.12	Non Essential
Rv0657c	31	4.30	30	4.61	Non Essential
Rv0658c	121	3.63	72	2.39	Non Essential
Rv0659c	227	15.83	234	18.09	Non Essential
Rv0660c	1387	121.61	1288	125.20	Non Essential
Rv0661c	5	0.25	5	0.27	Non Essential
Rv0662c	8	0.68	11	1.03	Non Essential
Rv0663	513	4.66	665	6.70	Non Essential
Rv0664	16	1.26	14	1.23	Non Essential
Rv0665	28	1.78	25	1.76	Non Essential
Rv0666	6	0.74	2	0.28	Essential
Rv0667	6084	37.15	6561	44.42	Essential
Rv0668	10927	59.42	9525	57.43	Essential
Rv0669c	96	1.08	200	2.49	Non Essential
Rv0670	160	4.53	196	6.16	Essential
Rv0671	78	1.99	58	1.64	Non Essential
Rv0672	438	5.78	391	5.72	Non Essential
Rv0673	183	4.19	119	3.02	Non Essential
Rv0674	89	2.65	61	2.01	Essential

Rv0675	70	1.90	76	2.29	Essential
Rv0676c	2152	15.97	671	5.52	Non Essential
Rv0677c	755	37.89	279	15.52	Non Essential
Rv0678	6257	270.43	1653	79.21	Non Essential
Rv0679c	61	2.64	48	2.30	Non Essential
Rv0680c	104	5.97	107	6.81	Non Essential
Rv0681	59	2.15	59	2.38	Non Essential
Rv0682	427	24.52	386	24.58	Essential
Rv0683	360	16.45	326	16.52	Essential
Rv0684	7552	77.06	7185	81.29	Essential
Rv0685	3537	63.85	3643	72.91	Essential
Rv0686	182	4.91	221	6.60	Non Essential
Rv0687	572	14.86	412	11.86	Essential
Rv0688	571	10.05	436	8.51	Non Essential
Rv0689c	0	0.00	0	0.00	Non Essential
Rv0690c	411	8.42	303	6.88	Non Essential
Rv0691c	237	8.54	181	7.23	Non Essential
Rv0692	259	16.91	248	17.95	Non Essential
Rv0693	1454	26.58	1188	24.08	Non Essential
Rv0694	1230	22.20	1048	20.97	Non Essential
Rv0695	561	15.96	388	12.24	Non Essential
Rv0696	479	7.29	424	7.15	Non Essential
Rv0697	157	2.34	130	2.15	Essential
Rv0698	55	1.93	39	1.52	Non Essential
Rv0699	7	0.68	10	1.08	Non Essential
Rv0700	2156	151.84	2606	203.49	Essential
Rv0701	2805	92.27	3579	130.53	Essential
Rv0702	1509	48.31	1899	67.40	Essential
Rv0703	1471	104.63	1888	148.89	Essential
Rv0704	880	22.45	1089	30.80	Essential
Rv0705	569	43.50	740	62.72	Essential
Rv0706	579	20.97	679	27.27	Essential
Rv0707	2552	66.53	3033	87.66	Essential
Rv0708	578	29.85	643	36.81	Essential
Rv0709	2139	197.20	2210	225.89	Essential
Rv0710	5213	273.12	5334	309.84	Essential
Rv0711	280	2.55	174	1.75	Non Essential
Rv0712	91	2.17	66	1.75	Non Essential
Rv0713	144	3.29	151	3.82	Non Essential
Rv0714	276	16.11	373	24.14	Essential
Rv0715	319	21.62	425	31.93	Essential
Rv0716	4910	187.33	6475	273.90	Essential
Rv0717	4309	500.32	5852	753.35	Non Essential
Rv0718	800	43.18	1090	65.22	Essential
Rv0719	513	20.44	709	31.33	Essential
Rv0720	1485	86.68	1793	116.04	Essential
Rv0721	4180	135.63	4583	164.88	Essential
Rv0722	860	93.77	952	115.09	Essential
Rv0723	1255	61.27	1259	68.15	Non Essential
Rv0724	1069	12.27	937	11.93	Non Essential

Rv0724A	27	1.73	31	2.20	Non Essential
Rv0725c	61	1.45	57	1.50	Non Essential
Rv0726c	35	0.68	35	0.76	Non Essential
Rv0727c	1	0.03	2	0.07	Non Essential
Rv0728c	4	0.09	6	0.15	Non Essential
Rv0729	74	1.18	96	1.70	Non Essential
Rv0730	658	19.42	681	22.28	Non Essential
Rv0731c	58	1.30	79	1.97	Non Essential
Rv0732	390	6.32	528	9.49	Essential
Rv0733	704	27.75	886	38.72	Non Essential
Rv0734	657	17.64	696	20.72	Non Essential
Rv0735	71	2.86	82	3.66	Non Essential
Rv0736	16	0.46	9	0.29	Essential
Rv0737	10	0.43	8	0.38	Non Essential
Rv0738	200	7.84	194	8.43	Non Essential
Rv0739	67	1.79	52	1.54	Non Essential
Rv0740	227	9.25	196	8.86	Non Essential
Rv0741	272	18.61	301	22.83	Non Essential
Rv0742	84	3.42	75	3.39	Non Essential
Rv0743c	39	1.50	33	1.41	Non Essential
Rv0744c	377	16.00	321	15.11	Essential
Rv0745	6	0.24	11	0.50	Non Essential
Rv0746	108	0.99	161	1.63	Non Essential
Rv0747	230	2.05	253	2.51	Non Essential
Rv0748	72	6.02	65	6.02	Non Essential
Rv0749	35	1.76	43	2.39	Non Essential
Rv0749A	33	5.17	23	4.00	Non Essential
Rv0750	81	7.10	77	7.48	Non Essential
Rv0751c	136	3.30	112	3.02	Non Essential
Rv0752c	252	4.62	173	3.52	Non Essential
Rv0753c	317	4.44	219	3.40	Non Essential
Rv0754	80	0.98	43	0.58	Non Essential
Rv0755c	846	9.38	720	8.85	Essential
Rv0755A	390	45.28	286	36.82	Non Essential
Rv0756c	116	3.44	115	3.78	Non Essential
Rv0757	368	10.64	306	9.81	Non Essential
Rv0758	721	10.63	579	9.46	Non Essential
Rv0759c	74	4.79	74	5.31	Non Essential
Rv0760c	129	6.61	147	8.36	Non Essential
Rv0761c	964	18.37	1463	30.92	Non Essential
Rv0762c	100	3.94	108	4.72	Non Essential
Rv0763c	14	1.46	7	0.81	Non Essential
Rv0764c	86	1.36	68	1.20	Non Essential
Rv0765c	31	0.81	20	0.58	Non Essential
Rv0766c	37	0.66	48	0.95	Non Essential
Rv0767c	14	0.47	12	0.45	Non Essential
Rv0768	94	1.37	65	1.05	Non Essential
Rv0769	51	1.47	51	1.63	Non Essential
Rv0770	12	0.29	20	0.54	Non Essential
Rv0771	4	0.20	21	1.15	Non Essential

Rv0772	39	0.66	66	1.24	Essential
Rv0773c	189	2.64	201	3.11	Non Essential
Rv0774c	261	6.15	252	6.59	Non Essential
Rv0775	167	5.76	146	5.58	Non Essential
Rv0776c	36	0.99	26	0.79	Non Essential
Rv0777	234	3.54	233	3.91	Non Essential
Rv0778	538	9.29	493	9.44	Non Essential
Rv0779c	3	0.10	4	0.15	Non Essential
Rv0780	43	1.03	65	1.73	Essential
Rv0781	94	2.84	91	3.05	Essential
Rv0782	123	1.59	142	2.04	Non Essential
Rv0783c	143	1.89	158	2.32	Non Essential
Rv0784	68	2.13	82	2.85	Non Essential
Rv0785	139	1.76	141	1.98	Non Essential
Rv0786c	31	1.71	24	1.47	Non Essential
Rv0787	13	0.29	18	0.45	Non Essential
Rv0787A	547	49.16	538	53.61	Non Essential
Rv0788	373	11.89	386	13.64	Essential
Rv0789c	19	0.68	13	0.52	Non Essential
Rv0790c	13	0.38	20	0.65	Non Essential
Rv0791c	54	1.11	46	1.05	Non Essential
Rv0792c	45	1.19	42	1.24	Non Essential
Rv0793	3	0.21	1	0.08	Non Essential
Rv0794c	95	1.36	140	2.22	Non Essential
Rv0795	9	0.59	10	0.73	Non Essential
Rv0796	9	0.20	10	0.24	Non Essential
Rv0797	7	0.14	7	0.15	Non Essential
Rv0798c	431	11.62	491	14.67	Essential
Rv0799c	124	2.65	138	3.26	Non Essential
Rv0800	135	2.23	140	2.56	Non Essential
Rv0801	49	3.03	47	3.23	Non Essential
Rv0802c	38	1.24	63	2.29	Non Essential
Rv0803	518	4.91	549	5.78	Essential
Rv0804	125	4.27	138	5.23	Non Essential
Rv0805	808	18.16	682	16.99	Non Essential
Rv0806c	49	0.66	39	0.58	Non Essential
Rv0807	50	2.76	48	2.94	Non Essential
Rv0808	789	10.71	699	10.52	Essential
Rv0809	272	5.34	237	5.16	Non Essential
Rv0810c	1069	126.17	1146	149.96	Non Essential
Rv0811c	475	9.23	338	7.28	Essential
Rv0812	65	1.61	65	1.78	Non Essential
Rv0813c	505	15.95	456	15.97	Non Essential
Rv0814c	790	56.19	975	76.89	Non Essential
Rv0815c	1600	41.26	2073	59.27	Non Essential
Rv0816c	13	0.66	9	0.51	Non Essential
Rv0817c	25	0.66	19	0.56	Essential
Rv0818	548	15.35	459	14.25	Non Essential
Rv0819	144	3.27	141	3.55	Non Essential
Rv0820	342	9.47	380	11.66	Non Essential

Rv0821c	291	9.75	225	8.36	Non Essential
Rv0822c	7214	75.44	6001	69.58	Non Essential
Rv0823c	4033	74.11	2917	59.43	Non Essential
Rv0824c	9994	211.29	8936	209.47	Essential
Rv0825c	29	0.97	25	0.93	Non Essential
Rv0826	5	0.10	9	0.20	Non Essential
Rv0827c	52	2.85	77	4.68	Non Essential
Rv0828c	8	0.41	4	0.23	Non Essential
Rv0829	59	4.37	64	5.26	Non Essential
Rv0830	37	0.88	30	0.79	Non Essential
Rv0831c	422	11.12	453	13.24	Non Essential
Rv0832	74	3.85	99	5.71	Non Essential
Rv0833	149	1.42	144	1.52	Non Essential
Rv0834c	572	4.64	465	4.18	Non Essential
Rv0835	194	6.47	108	3.99	Non Essential
Rv0836c	3	0.10	5	0.18	Non Essential
Rv0837c	13	0.27	8	0.19	Non Essential
Rv0838	15	0.42	34	1.05	Non Essential
Rv0839	375	9.92	189	5.54	Non Essential
Rv0840c	21	0.52	18	0.50	Non Essential
Rv0841	0	0.00	0	0.00	Non Essential
Rv0842	31	0.52	43	0.79	Non Essential
Rv0843	40	0.86	40	0.95	Non Essential
Rv0844c	233	7.70	235	8.61	Non Essential
Rv0845	117	1.97	86	1.60	Non Essential
Rv0846c	34	0.48	47	0.74	Non Essential
Rv0847	47	2.58	55	3.34	Non Essential
Rv0848	33	0.63	74	1.58	Non Essential
Rv0849	6	0.10	14	0.26	Non Essential
Rv0850	4	0.26	6	0.43	Non Essential
Rv0851c	73	1.90	67	1.93	Non Essential
Rv0852	34	0.87	53	1.51	Non Essential
Rv0853c	135	1.72	138	1.95	Non Essential
Rv0854	69	3.35	40	2.15	Non Essential
Rv0855	83	1.65	52	1.15	Non Essential
Rv0856	165	8.77	153	9.02	Non Essential
Rv0857	78	3.54	64	3.22	Non Essential
Rv0858c	39	0.70	40	0.80	Non Essential
Rv0859	1950	34.59	935	18.39	Non Essential
Rv0860	6750	67.06	2509	27.64	Non Essential
Rv0861c	281	3.71	228	3.34	Non Essential
Rv0862c	211	2.00	205	2.15	Non Essential
Rv0863	90	6.88	125	10.59	Non Essential
Rv0864	908	38.78	809	38.30	Non Essential
Rv0865	369	16.44	306	15.12	Non Essential
Rv0866	186	9.40	172	9.64	Non Essential
Rv0867c	70	1.23	96	1.87	Non Essential
Rv0868c	67	5.18	50	4.28	Non Essential
Rv0869c	152	3.02	135	2.97	Non Essential
Rv0870c	169	9.33	101	6.18	Non Essential

Rv0871	140	7.39	173	10.12	Non Essential
Rv0872c	1591	18.78	1277	16.71	Non Essential
Rv0873	1022	11.25	963	11.75	Non Essential
Rv0874c	76	1.41	71	1.46	Non Essential
Rv0875c	38	1.67	37	1.81	Essential
Rv0876c	715	9.33	708	10.24	Non Essential
Rv0877	297	8.10	231	6.98	Non Essential
Rv0878c	84	1.36	91	1.63	Non Essential
Rv0879c	120	9.37	167	14.46	Non Essential
Rv0880	44	2.19	57	3.15	Non Essential
Rv0881	50	1.24	36	0.99	Non Essential
Rv0882	10	0.76	7	0.59	Non Essential
Rv0883c	368	10.39	339	10.61	Essential
Rv0884c	258	4.90	237	4.99	Essential
Rv0885	519	10.91	301	7.01	Non Essential
Rv0886	218	2.71	145	2.00	Non Essential
Rv0887c	8	0.38	4	0.21	Non Essential
Rv0888	153	2.23	123	1.99	Non Essential
Rv0889c	420	8.05	385	8.18	Non Essential
Rv0890c	372	3.02	393	3.53	Non Essential
Rv0891c	61	1.53	64	1.78	Non Essential
Rv0892	205	2.96	257	4.12	Non Essential
Rv0893c	41	0.90	61	1.49	Non Essential
Rv0894	9	0.16	14	0.28	Essential
Rv0895	21	0.30	19	0.30	Non Essential
Rv0896	2582	42.83	3819	70.23	Non Essential
Rv0897c	216	2.89	156	2.31	Non Essential
Rv0898c	202	16.50	181	16.39	Non Essential
Rv0899	49	1.07	65	1.58	Non Essential
Rv0900	27	3.82	27	4.23	Essential
Rv0901	160	6.52	126	5.69	Non Essential
Rv0902c	440	7.05	342	6.08	Non Essential
Rv0903c	532	16.10	508	17.04	Non Essential
Rv0904c	83	1.20	113	1.81	Non Essential
Rv0905	172	5.05	211	6.87	Non Essential
Rv0906	154	2.96	171	3.64	Non Essential
Rv0907	257	3.45	202	3.01	Non Essential
Rv0908	228	2.05	175	1.74	Non Essential
Rv0909	484	58.08	458	60.94	Non Essential
Rv0910	438	21.68	432	23.71	Non Essential
Rv0911	79	2.20	89	2.74	Non Essential
Rv0912	57	2.73	64	3.39	Non Essential
Rv0913c	327	4.66	246	3.89	Non Essential
Rv0914c	437	7.58	394	7.58	Non Essential
Rv0915c	24	0.41	18	0.34	Non Essential
Rv0916c	2	0.14	3	0.24	Non Essential
Rv0917	98	1.18	91	1.22	Non Essential
Rv0918	13	0.59	8	0.40	Non Essential
Rv0919	18	0.77	15	0.71	Non Essential
Rv0920c	218	3.55	241	4.35	Non Essential

Rv0921	291	10.76	221	9.06	Non Essential
Rv0922	2221	28.88	1755	25.30	Non Essential
Rv0923c	33	0.67	35	0.78	Non Essential
Rv0924c	62	1.04	60	1.11	Non Essential
Rv0925c	113	3.29	143	4.62	Non Essential
Rv0926c	186	3.71	235	5.20	Non Essential
Rv0927c	32	0.87	32	0.96	Non Essential
Rv0928	117	2.26	146	3.13	Non Essential
Rv0929	78	1.72	72	1.76	Non Essential
Rv0930	58	1.35	76	1.95	Non Essential
Rv0931c	1528	16.46	1284	15.34	Non Essential
Rv0932c	1231	23.78	1106	23.69	Non Essential
Rv0933	216	5.59	261	7.49	Non Essential
Rv0934	781	14.93	866	18.35	Non Essential
Rv0935	818	17.29	665	15.59	Non Essential
Rv0936	580	13.77	512	13.47	Non Essential
Rv0937c	158	4.13	165	4.79	Non Essential
Rv0938	786	7.41	595	6.22	Non Essential
Rv0939	1289	14.32	1066	13.13	Non Essential
Rv0940c	286	7.09	195	5.36	Non Essential
Rv0941c	478	13.28	292	9.00	Non Essential
Rv0942	0	0.00	0	0.00	Non Essential
Rv0943c	2	0.04	2	0.05	Non Essential
Rv0944	18	0.81	16	0.80	Non Essential
Rv0945	209	5.90	285	8.92	Non Essential
Rv0946c	134	1.73	162	2.32	Essential
Rv0947c	44	4.11	39	4.04	
Rv0948c	69	4.68	64	4.81	Non Essential
Rv0949	405	3.76	370	3.81	Essential
Rv0950c	188	4.05	295	7.04	Essential
Rv0951	579	10.69	687	14.07	Essential
Rv0952	441	10.40	518	13.54	Essential
Rv0953c	64	1.62	42	1.18	Non Essential
Rv0954	617	14.55	751	19.63	Non Essential
Rv0955	340	5.34	369	6.43	Essential
Rv0956	543	18.03	682	25.10	Non Essential
Rv0957	590	8.07	795	12.05	Essential
Rv0958	256	3.99	231	3.99	Non Essential
Rv0959	336	3.58	306	3.61	Non Essential
Rv0959A	173	16.82	103	11.10	
Rv0960	152	8.52	106	6.59	Non Essential
Rv0961	80	4.95	81	5.56	Non Essential
Rv0962c	0	0.00	2	0.07	Non Essential
Rv0963c	13	0.35	9	0.27	Non Essential
Rv0964c	7	0.31	3	0.15	Non Essential
Rv0965c	108	5.54	118	6.71	Non Essential
Rv0966c	397	14.17	458	18.12	Non Essential
Rv0967	629	37.64	397	26.34	Non Essential
Rv0968	403	29.25	232	18.67	Non Essential
Rv0969	583	5.42	344	3.54	Non Essential

Rv0970	448	15.23	408	15.37	Non Essential
Rv0971c	109	2.89	44	1.30	Non Essential
Rv0972c	239	4.40	71	1.45	Non Essential
Rv0973c	468	5.02	111	1.32	Essential
Rv0974c	281	3.80	81	1.21	Non Essential
Rv0975c	199	3.72	63	1.31	Non Essential
Rv0976c	162	2.07	91	1.29	Non Essential
Rv0977	203	1.57	184	1.58	Non Essential
Rv0978c	8	0.17	5	0.12	Non Essential
Rv0979c	14	1.55	24	2.95	Non Essential
Rv0979A	40	4.97	49	6.75	Non Essential
Rv0980c	20	0.31	27	0.47	Non Essential
Rv0981	219	6.86	200	6.94	Non Essential
Rv0982	777	11.02	601	9.45	Essential
Rv0983	854	13.16	689	11.77	Non Essential
Rv0984	1218	48.01	1040	45.45	Non Essential
Rv0985c	6	0.28	10	0.52	Non Essential
Rv0986	3	0.09	8	0.26	Non Essential
Rv0987	276	2.31	252	2.34	Non Essential
Rv0988	172	3.19	134	2.75	Non Essential
Rv0989c	14	0.31	24	0.59	Non Essential
Rv0990c	11	0.36	14	0.51	Non Essential
Rv0991c	35	2.26	28	2.01	Non Essential
Rv0992c	90	3.26	132	5.30	Non Essential
Rv0993	883	20.62	942	24.39	Essential
Rv0994	850	14.26	814	15.15	Non Essential
Rv0995	123	4.32	102	3.98	Non Essential
Rv0996	927	18.51	879	19.46	Non Essential
Rv0997	135	6.73	131	7.24	Non Essential
Rv0998	238	5.11	222	5.28	Non Essential
Rv0999	143	4.05	147	4.62	Non Essential
Rv1000c	100	3.48	106	4.09	Non Essential
Rv1001	205	3.65	211	4.16	Non Essential
Rv1002c	141	2.00	185	2.92	Essential
Rv1003	43	1.08	41	1.14	Non Essential
Rv1004c	69	1.18	51	0.96	Non Essential
Rv1005c	89	1.39	54	0.93	Essential
Rv1006	850	10.72	675	9.44	Non Essential
Rv1007c	101	1.39	127	1.94	Essential
Rv1008	11	0.30	20	0.60	Non Essential
Rv1009	598	11.81	596	13.05	Non Essential
Rv1010	252	5.68	247	6.17	Non Essential
Rv1011	89	2.08	78	2.02	Essential
Rv1012	17	1.25	20	1.63	Non Essential
Rv1013	500	6.57	461	6.72	Essential
Rv1014c	33	1.23	30	1.24	Essential
Rv1015c	94	3.12	159	5.85	Non Essential
Rv1016c	70	2.21	81	2.84	Essential
Rv1017c	408	8.94	489	11.88	Essential
Rv1018c	291	4.20	327	5.24	Essential

Rv1019	104	3.77	100	4.02	Non Essential
Rv1020	698	4.05	671	4.31	Non Essential
Rv1021	244	5.36	198	4.83	Essential
Rv1022	86	2.53	74	2.41	Non Essential
Rv1023	394	6.57	364	6.73	Essential
Rv1024	96	3.01	90	3.12	Essential
Rv1025	198	9.11	209	10.66	Essential
Rv1026	117	2.62	113	2.81	Essential
Rv1027c	18	0.57	19	0.67	Essential
Rv1028c	114	0.95	127	1.17	Essential
Rv1028A	7	1.63	7	1.81	Non Essential
Rv1029	54	0.68	67	0.93	Non Essential
Rv1030	230	2.32	283	3.17	Non Essential
Rv1031	31	1.17	33	1.38	Non Essential
Rv1032c	93	1.31	88	1.37	Non Essential
Rv1033c	146	4.06	101	3.11	Non Essential
Rv1034c	0	0.00	3	0.18	Non Essential
Rv1035c	35	1.10	33	1.15	Non Essential
Rv1036c	206	13.09	268	18.88	Non Essential
Rv1037c	124	9.38	128	10.73	Non Essential
Rv1038c	335	24.31	317	25.51	Non Essential
Rv1039c	4	0.07	5	0.10	Non Essential
Rv1040c	3	0.08	1	0.03	Non Essential
Rv1041c	212	5.28	299	8.25	Non Essential
Rv1042c	292	15.41	393	23.00	Non Essential
Rv1043c	322	6.75	282	6.55	Non Essential
Rv1044	9	0.31	8	0.31	Non Essential
Rv1045	48	1.17	43	1.16	Non Essential
Rv1046c	86	3.53	68	3.09	Non Essential
Rv1047	1	0.02	4	0.08	Non Essential
Rv1048c	49	0.94	33	0.70	Non Essential
Rv1049	19	0.92	22	1.17	Non Essential
Rv1050	39	0.93	44	1.16	Non Essential
Rv1051c	7	0.20	3	0.09	Non Essential
Rv1052	116	6.41	151	9.24	Non Essential
Rv1053c	0	0.00	0	0.00	Non Essential
Rv1054	128	8.76	146	11.07	Non Essential
Rv1055	266	42.64	382	67.89	Non Essential
Rv1056	39	1.10	35	1.09	Non Essential
Rv1057	31	0.56	48	0.97	Non Essential
Rv1058	88	1.16	106	1.55	Non Essential
Rv1059	343	6.92	366	8.19	Non Essential
Rv1060	71	3.22	127	6.39	Non Essential
Rv1061	180	4.48	143	3.95	Non Essential
Rv1062	102	2.56	111	3.08	Non Essential
Rv1063c	68	1.35	92	2.03	Non Essential
Rv1064c	13	0.67	15	0.85	Non Essential
Rv1065	94	3.57	79	3.32	Non Essential
Rv1066	55	2.99	45	2.71	Non Essential
Rv1067c	32	0.34	36	0.43	Non Essential

Rv1068c	112	1.73	100	1.71	Non Essential
Rv1069c	402	4.90	360	4.86	Non Essential
Rv1070c	176	4.89	210	6.47	Non Essential
Rv1071c	182	3.77	233	5.35	Non Essential
Rv1072	3998	102.73	3950	112.53	Non Essential
Rv1073	1477	37.28	1008	28.21	Non Essential
Rv1074c	583	10.29	538	10.53	Non Essential
Rv1075c	367	8.35	362	9.13	Non Essential
Rv1076	252	6.06	228	6.08	Non Essential
Rv1077	941	14.50	958	16.37	Non Essential
Rv1078	1606	47.78	2204	72.70	Non Essential
Rv1079	691	12.73	857	17.50	Non Essential
Rv1080c	796	34.61	618	29.79	Non Essential
Rv1081c	107	5.30	80	4.39	Non Essential
Rv1082	367	9.10	349	9.60	Non Essential
Rv1083	40	3.23	47	4.21	Non Essential
Rv1084	204	2.17	167	1.97	Non Essential
Rv1085c	131	3.87	111	3.63	Non Essential
Rv1086	122	3.33	104	3.14	Non Essential
Rv1087	307	2.86	278	2.87	Non Essential
Rv1087A	1	0.07	0	0.00	Non Essential
Rv1088	13	0.64	7	0.38	Non Essential
Rv1089	0	0.00	3	0.20	Non Essential
Rv1089A	0	0.00	0	0.00	Non Essential
Rv1090	39	1.84	54	2.83	Non Essential
Rv1091	344	2.89	341	3.17	Non Essential
Rv1092c	166	3.80	185	4.70	Essential
Rv1093	632	10.61	601	11.18	Non Essential
Rv1094	6396	166.13	5947	171.26	Essential
Rv1095	1213	20.03	1000	18.31	Non Essential
Rv1096	168	4.12	159	4.33	Non Essential
Rv1097c	106	2.58	120	3.24	Non Essential
Rv1098c	757	11.42	852	14.25	Essential
Rv1099c	186	3.67	183	4.01	Essential
Rv1100	161	4.93	139	4.72	Non Essential
Rv1101c	1265	23.49	976	20.09	Non Essential
Rv1102c	40	2.76	23	1.76	Non Essential
Rv1103c	45	3.02	49	3.65	Non Essential
Rv1104	73	2.28	95	3.28	Non Essential
Rv1105	1	0.04	10	0.46	Non Essential
Rv1106c	255	4.93	156	3.34	Non Essential
Rv1107c	764	63.86	649	60.14	Non Essential
Rv1108c	460	7.92	408	7.79	Non Essential
Rv1109c	242	8.15	260	9.71	Essential
Rv1110	298	6.36	233	5.51	Non Essential
Rv1111c	308	6.73	384	9.30	Essential
Rv1112	48	0.96	52	1.15	Non Essential
Rv1113	43	4.69	68	8.22	Non Essential
Rv1114	137	7.87	156	9.93	Non Essential
Rv1115	23	0.71	22	0.75	Non Essential

Rv1116	3	0.35	7	0.90	Non Essential
Rv1116A	74	5.78	83	7.19	Non Essential
Rv1117	122	8.11	88	6.49	Non Essential
Rv1118c	51	1.27	63	1.74	Non Essential
Rv1119c	1	0.14	2	0.32	Non Essential
Rv1120c	1	0.04	3	0.14	Non Essential
Rv1121	554	8.50	492	8.37	Non Essential
Rv1122	358	7.52	344	8.02	Essential
Rv1123c	97	2.29	126	3.30	Non Essential
Rv1124	143	3.23	175	4.39	Non Essential
Rv1125	37	0.64	43	0.82	Non Essential
Rv1126c	254	9.02	217	8.54	Essential
Rv1127c	307	4.48	357	5.78	Non Essential
Rv1128c	35	0.55	49	0.86	Essential
Rv1129c	33	0.49	30	0.49	Non Essential
Rv1130	19	0.26	31	0.47	Non Essential
Rv1131	13	0.24	16	0.32	Non Essential
Rv1132	130	1.61	142	1.95	Non Essential
Rv1133c	2387	22.50	2667	27.87	Essential
Rv1134	27	2.46	19	1.92	Non Essential
Rv1135c	65	0.75	55	0.71	Non Essential
Rv1135A	4	0.36	7	0.69	Non Essential
Rv1136	5	0.31	17	1.19	Non Essential
Rv1137c	3	0.18	1	0.06	Non Essential
Rv1138c	45	0.95	42	0.98	Non Essential
Rv1139c	22	0.95	19	0.91	Non Essential
Rv1140	126	3.19	170	4.77	Non Essential
Rv1141c	25	0.67	30	0.89	Non Essential
Rv1142c	631	16.82	701	20.71	Non Essential
Rv1143	136	2.70	169	3.72	Non Essential
Rv1144	69	1.97	100	3.17	Essential
Rv1145	20	0.47	17	0.44	Non Essential
Rv1146	54	0.82	41	0.69	Non Essential
Rv1147	36	1.19	28	1.03	Non Essential
Rv1148c	105	1.56	110	1.81	Non Essential
Rv1149	309	16.31	358	20.95	Non Essential
Rv1150	14	0.55	16	0.69	
Rv1151c	76	2.29	78	2.61	Non Essential
Rv1152	274	16.13	243	15.86	Non Essential
Rv1153c	13	0.33	21	0.59	Non Essential
Rv1154c	78	2.61	47	1.75	Non Essential
Rv1155	736	35.69	673	36.18	Non Essential
Rv1156	837	30.63	934	37.89	Non Essential
Rv1157c	256	4.93	280	5.98	Non Essential
Rv1158c	97	3.05	127	4.43	Non Essential
Rv1159	62	1.03	49	0.90	Non Essential
Rv1159A	107	8.09	130	10.90	Non Essential
Rv1160	40	2.02	39	2.19	Non Essential
Rv1161	2268	13.17	1749	11.26	Non Essential
Rv1162	740	9.48	474	6.74	Non Essential

Rv1163	96	3.41	65	2.56	Non Essential
Rv1164	159	4.62	141	4.54	Non Essential
Rv1165	395	4.50	281	3.55	Non Essential
Rv1166	269	3.03	227	2.83	Essential
Rv1167c	119	4.23	111	4.37	Non Essential
Rv1168c	1076	22.22	1264	28.95	Non Essential
Rv1169c	4	0.28	3	0.24	Essential
Rv1170	51	1.20	49	1.28	Non Essential
Rv1171	99	4.83	46	2.49	Non Essential
Rv1172c	1213	28.14	1361	35.00	Non Essential
Rv1173	1449	12.11	1463	13.56	Non Essential
Rv1174c	467	30.21	380	27.26	Non Essential
Rv1175c	338	3.59	334	3.93	Non Essential
Rv1176c	54	2.04	37	1.55	Non Essential
Rv1177	1273	83.88	1442	105.34	Essential
Rv1178	2556	50.46	2380	52.10	Non Essential
Rv1179c	489	3.73	376	3.18	Non Essential
Rv1180	1756	25.73	1259	20.45	Non Essential
Rv1181	276	1.25	250	1.25	Non Essential
Rv1182	86	1.30	100	1.68	Essential
Rv1183	145	1.04	95	0.75	Essential
Rv1184c	301	5.99	152	3.35	Essential
Rv1185c	859	10.63	727	9.97	Essential
Rv1186c	50	0.66	23	0.34	Essential
Rv1187	93	1.22	66	0.96	Essential
Rv1188	26	0.56	11	0.26	Essential
Rv1189	18	0.44	20	0.55	Non Essential
Rv1190	7	0.17	11	0.30	Non Essential
Rv1191	36	0.85	39	1.02	Non Essential
Rv1192	147	3.82	164	4.72	Essential
Rv1193	404	6.11	361	6.05	Essential
Rv1194c	125	2.12	156	2.94	Essential
Rv1195	531	38.15	884	70.41	Non Essential
Rv1196	1692	30.93	2009	40.72	Non Essential
Rv1197	693	50.29	756	60.83	Non Essential
Rv1198	375	28.36	464	38.91	Non Essential
Rv1199c	4	0.07	2	0.04	Non Essential
Rv1200	216	3.63	184	3.43	Non Essential
Rv1201c	312	7.03	451	11.27	Essential
Rv1202	379	7.65	268	6.00	Essential
Rv1203c	18	0.66	19	0.77	Non Essential
Rv1204c	171	2.18	185	2.61	Essential
Rv1205	305	11.64	298	12.61	Non Essential
Rv1206	231	2.77	210	2.79	Non Essential
Rv1207	400	8.99	436	10.86	Non Essential
Rv1208	356	7.85	312	7.63	Essential
Rv1209	270	15.76	249	16.11	Non Essential
Rv1210	67	2.34	55	2.13	Non Essential
Rv1211	771	72.96	822	86.24	Essential
Rv1212c	78	1.44	138	2.83	Non Essential

Rv1213	217	3.84	197	3.86	Non Essential
Rv1214c	88	5.69	111	7.96	Non Essential
Rv1215c	184	2.35	195	2.76	Essential
Rv1216c	64	2.04	49	1.73	Non Essential
Rv1217c	174	2.27	215	3.11	Non Essential
Rv1218c	96	2.21	78	1.99	Non Essential
Rv1219c	93	3.13	98	3.66	Non Essential
Rv1220c	752	24.97	744	27.39	Non Essential
Rv1221	317	8.81	271	8.35	Non Essential
Rv1222	104	4.81	86	4.41	Non Essential
Rv1223	2467	33.41	2017	30.29	Essential
Rv1224	298	16.21	205	12.36	Essential
Rv1225c	27	0.70	30	0.86	Non Essential
Rv1226c	82	1.20	79	1.29	Non Essential
Rv1227c	18	0.73	23	1.03	Non Essential
Rv1228	23	0.89	22	0.94	Non Essential
Rv1229c	734	13.45	700	14.22	Essential
Rv1230c	564	9.81	496	9.56	Non Essential
Rv1231c	118	4.68	119	5.23	Non Essential
Rv1232c	154	2.53	167	3.04	Essential
Rv1233c	1737	62.60	1952	78.00	Non Essential
Rv1234	126	5.14	131	5.92	Non Essential
Rv1235	69	1.05	81	1.37	Essential
Rv1236	103	2.40	65	1.68	Essential
Rv1237	147	3.83	135	3.90	Essential
Rv1238	413	7.51	373	7.52	Essential
Rv1239c	64	1.25	77	1.67	Non Essential
Rv1240	286	6.21	489	11.78	Non Essential
Rv1241	89	7.35	131	12.00	Non Essential
Rv1242	20	1.00	27	1.49	Non Essential
Rv1243c	78	0.99	71	1.00	Non Essential
Rv1244	99	2.47	161	4.46	Essential
Rv1245c	99	2.56	80	2.30	Non Essential
Rv1246c	10	0.73	12	0.98	Non Essential
Rv1247c	26	2.08	21	1.86	Non Essential
Rv1248c	2367	13.76	2413	15.55	Essential
Rv1249c	189	5.15	191	5.77	Non Essential
Rv1250	110	1.36	117	1.60	Non Essential
Rv1251c	582	3.66	717	4.99	Non Essential
Rv1252c	77	2.72	114	4.47	Non Essential
Rv1253	411	5.22	411	5.79	Non Essential
Rv1254	93	1.74	93	1.92	Essential
Rv1255c	36	1.27	21	0.82	Non Essential
Rv1256c	48	0.85	36	0.70	Non Essential
Rv1257c	337	5.30	368	6.41	Non Essential
Rv1258c	79	1.35	121	2.29	Non Essential
Rv1259	159	3.80	132	3.50	Non Essential
Rv1260	512	9.84	552	11.76	Non Essential
Rv1261c	117	5.60	98	5.20	Non Essential
Rv1262c	26	1.29	36	1.98	Non Essential

Rv1263	23	0.36	59	1.01	Non Essential
Rv1264	792	14.26	754	15.05	Non Essential
Rv1265	215	6.79	201	7.04	Non Essential
Rv1266c	238	2.72	253	3.20	Non Essential
Rv1267c	46	0.85	47	0.96	Non Essential
Rv1268c	2	0.06	1	0.03	Non Essential
Rv1269c	26	1.49	35	2.23	Non Essential
Rv1270c	50	1.46	47	1.52	Non Essential
Rv1271c	10	0.63	8	0.56	Non Essential
Rv1272c	169	1.92	175	2.20	Essential
Rv1273c	98	1.20	103	1.40	Non Essential
Rv1274	491	18.94	606	25.91	Essential
Rv1275	354	14.03	415	18.24	Non Essential
Rv1276c	8	0.36	9	0.45	Non Essential
Rv1277	156	2.67	147	2.79	Non Essential
Rv1278	252	2.06	263	2.38	Essential
Rv1279	159	2.15	165	2.48	Non Essential
Rv1280c	416	5.03	397	5.33	Non Essential
Rv1281c	149	1.74	130	1.68	Non Essential
Rv1282c	74	1.82	73	1.99	Non Essential
Rv1283c	231	5.08	207	5.05	Non Essential
Rv1284	53	2.32	54	2.62	Essential
Rv1285	262	5.64	217	5.18	Essential
Rv1286	205	2.39	300	3.87	Essential
Rv1287	58	2.57	86	4.22	Non Essential
Rv1288	55	0.86	47	0.82	Non Essential
Rv1289	219	7.44	283	10.66	Non Essential
Rv1290c	29	0.40	35	0.53	Non Essential
Rv1290A	7	0.48	0	0.00	Non Essential
Rv1291c	1	0.06	2	0.14	Non Essential
Rv1292	97	1.26	117	1.69	Essential
Rv1293	647	10.35	742	13.16	Essential
Rv1294	312	5.06	366	6.58	Essential
Rv1295	736	14.61	677	14.90	Essential
Rv1296	2128	48.12	2162	54.20	Essential
Rv1297	14532	172.65	11380	149.90	Essential
Rv1298	13043	1157.73	8972	882.96	Essential
Rv1299	1393	27.89	1001	22.22	Essential
Rv1300	1048	23.04	864	21.06	Essential
Rv1301	617	20.30	494	18.02	Essential
Rv1302	251	4.44	205	4.02	Non Essential
Rv1303	159	7.04	220	10.80	Essential
Rv1304	220	6.28	361	11.43	Essential
Rv1305	912	79.96	1354	131.62	Essential
Rv1306	1003	41.83	1223	56.56	Essential
Rv1307	2285	36.63	2466	43.83	Essential
Rv1308	1041	13.56	1161	16.77	Essential
Rv1309	727	17.03	680	17.66	Essential
Rv1310	2018	29.69	2091	34.11	Essential
Rv1311	1589	93.51	1497	97.68	Essential

Rv1312	2265	109.83	1973	106.07	Non Essential
Rv1313c	203	3.27	166	2.96	Non Essential
Rv1314c	98	3.62	97	3.98	Non Essential
Rv1315	223	3.81	223	4.23	Essential
MTB000019	15788348	220794.81	14631628	226864.56	
MTB000020	27866701	190815.84	24781216	188136.61	
MTB000021	36319	6843.41	37488	7831.64	
Rv1316c	15	0.65	7	0.34	Non Essential
Rv1317c	54	0.78	43	0.69	Non Essential
Rv1318c	170	2.25	151	2.21	Non Essential
Rv1319c	168	2.25	149	2.21	Non Essential
Rv1320c	207	2.61	165	2.31	Non Essential
Rv1321	286	9.03	257	9.00	Non Essential
Rv1322	20	1.45	15	1.21	Non Essential
Rv1322A	826	38.74	786	40.87	Non Essential
Rv1323	1143	21.00	935	19.05	Essential
Rv1324	645	15.16	660	17.20	Non Essential
Rv1325c	314	3.72	340	4.47	Non Essential
Rv1326c	401	3.92	344	3.73	Essential
Rv1327c	1469	14.99	1661	18.79	Essential
Rv1328	992	8.22	1061	9.75	Non Essential
Rv1329c	119	1.28	95	1.13	Non Essential
Rv1330c	31	0.49	41	0.73	Non Essential
Rv1331	739	52.05	825	64.42	Non Essential
Rv1332	1458	47.74	1405	51.01	Essential
Rv1333	288	5.98	212	4.88	Essential
Rv1334	162	7.91	113	6.12	Non Essential
Rv1335	48	3.67	46	3.90	Non Essential
Rv1336	737	16.30	612	15.01	Non Essential
Rv1337	694	20.65	554	18.27	Non Essential
Rv1338	168	4.43	123	3.59	Essential
Rv1339	246	6.44	217	6.29	Essential
Rv1340	337	9.29	302	9.23	Non Essential
Rv1341	82	2.87	74	2.87	Non Essential
Rv1342c	73	4.33	70	4.61	Essential
Rv1343c	21	1.19	30	1.88	Non Essential
Rv1344	738	49.54	432	32.15	Non Essential
Rv1345	352	4.83	215	3.27	Non Essential
Rv1346	431	7.98	268	5.50	Non Essential
Rv1347c	275	9.35	47	1.77	Essential
Rv1348	1641	13.67	305	2.82	Essential
Rv1349	1261	15.58	168	2.30	Essential
Rv1350	132	3.82	68	2.18	Essential
Rv1351	56	3.66	36	2.61	Non Essential
Rv1352	222	12.85	178	11.43	Non Essential
Rv1353c	11	0.30	8	0.24	Non Essential
Rv1354c	23	0.26	40	0.51	Non Essential
Rv1355c	124	1.24	175	1.94	Non Essential
Rv1356c	7	0.19	9	0.27	Non Essential
Rv1357c	4	0.09	9	0.23	Non Essential

Rv1358	69	0.43	65	0.44	Non Essential
Rv1359	21	0.60	19	0.60	Non Essential
Rv1360	319	6.70	305	7.11	Non Essential
Rv1361c	1346	24.30	1326	26.54	Non Essential
Rv1362c	42	1.36	51	1.83	Non Essential
Rv1363c	540	14.78	599	18.17	Non Essential
Rv1364c	155	1.70	134	1.63	Non Essential
Rv1365c	12	0.67	7	0.43	Non Essential
Rv1366	100	2.62	159	4.61	Non Essential
Rv1367c	128	2.43	121	2.54	Non Essential
Rv1368	186	5.09	228	6.92	Non Essential
Rv1369c	17	0.37	12	0.29	Non Essential
Rv1370c	12	0.79	13	0.95	Non Essential
Rv1371	2	0.03	2	0.03	Essential
Rv1372	69	1.25	65	1.31	Non Essential
Rv1373	69	1.51	51	1.24	Non Essential
Rv1374c	5	0.23	11	0.57	Non Essential
Rv1375	165	2.69	152	2.74	Non Essential
Rv1376	115	1.65	143	2.28	Non Essential
Rv1377c	134	4.51	118	4.40	Non Essential
Rv1378c	179	2.69	178	2.97	Non Essential
Rv1379	62	2.29	94	3.85	Non Essential
Rv1380	137	3.07	175	4.35	Essential
Rv1381	129	2.14	174	3.21	Essential
Rv1382	49	2.12	78	3.74	Essential
Rv1383	114	2.17	142	2.99	Essential
Rv1384	775	4.97	864	6.15	Non Essential
Rv1385	54	1.41	57	1.65	Non Essential
Rv1386	80	5.58	84	6.50	Non Essential
Rv1387	2286	30.33	2754	40.51	Non Essential
Rv1388	890	33.42	1283	53.42	Essential
Rv1389	224	7.69	345	13.13	Essential
Rv1390	297	19.22	302	21.66	Essential
Rv1391	649	11.10	606	11.49	Essential
Rv1392	755	13.39	668	13.14	Essential
Rv1393c	100	1.45	85	1.37	Non Essential
Rv1394c	266	4.13	355	6.10	Non Essential
Rv1395	18	0.37	21	0.48	Non Essential
Rv1396c	2514	31.21	2154	29.65	Non Essential
Rv1397c	212	11.36	163	9.68	Non Essential
Rv1398c	4471	373.69	3557	329.62	Non Essential
Rv1399c	113	2.53	147	3.65	Non Essential
Rv1400c	108	2.41	152	3.76	Non Essential
Rv1401	127	4.53	131	5.18	Non Essential
Rv1402	193	2.11	178	2.16	Non Essential
Rv1403c	6	0.16	8	0.23	Non Essential
Rv1404	493	21.97	537	26.53	Non Essential
Rv1405c	17	0.44	25	0.72	Essential
Rv1406	69	1.58	43	1.09	Essential
Rv1407	104	1.63	83	1.44	Non Essential

Rv1408	486	14.96	423	14.43	Non Essential
Rv1409	270	5.69	268	6.26	Essential
Rv1410c	2343	32.34	1880	28.77	Essential
Rv1411c	1797	54.37	1605	53.84	Essential
Rv1412	236	8.38	233	9.17	Non Essential
Rv1413	24	1.00	22	1.02	Non Essential
Rv1414	15	0.80	19	1.13	Non Essential
Rv1415	1775	29.86	1657	30.90	Essential
Rv1416	172	7.67	183	9.04	Essential
Rv1417	158	7.31	130	6.67	Non Essential
Rv1418	187	5.86	198	6.87	Non Essential
Rv1419	47	2.13	37	1.86	Non Essential
Rv1420	554	6.13	536	6.58	Essential
Rv1421	303	7.19	315	8.29	Non Essential
Rv1422	391	8.17	360	8.34	Essential
Rv1423	763	16.78	777	18.94	Non Essential
Rv1424c	15	0.42	36	1.13	Non Essential
Rv1425	336	5.23	241	4.16	Non Essential
Rv1426c	259	4.41	287	5.42	Non Essential
Rv1427c	401	5.36	405	6.00	Non Essential
Rv1428c	80	2.08	85	2.45	Non Essential
Rv1429	153	2.59	149	2.80	Non Essential
Rv1430	52	0.70	50	0.75	Non Essential
Rv1431	74	0.90	74	1.00	Non Essential
Rv1432	24	0.36	18	0.30	Non Essential
Rv1433	137	3.61	189	5.52	Non Essential
Rv1434	10	1.57	11	1.91	Non Essential
Rv1435c	418	14.77	332	13.00	Non Essential
Rv1436	605	12.75	801	18.72	Essential
Rv1437	1072	18.60	1010	19.43	Essential
Rv1438	505	13.82	456	13.83	Essential
Rv1439c	44	2.22	26	1.46	Non Essential
Rv1440	92	8.48	106	10.83	Non Essential
Rv1441c	305	4.44	317	5.12	Non Essential
Rv1442	232	2.17	151	1.56	Non Essential
Rv1443c	171	7.57	163	8.00	Non Essential
Rv1444c	302	15.82	283	16.44	Non Essential
Rv1445c	230	6.65	204	6.54	Non Essential
Rv1446c	247	5.82	183	4.78	Essential
Rv1447c	572	7.96	439	6.77	Non Essential
Rv1448c	315	6.04	350	7.44	Essential
Rv1449c	1496	15.29	1489	16.87	Essential
Rv1450c	59	0.32	97	0.58	Non Essential
Rv1451	152	3.53	220	5.66	Non Essential
Rv1452c	106	1.02	82	0.88	Non Essential
Rv1453	8	0.14	10	0.19	Non Essential
Rv1454c	67	1.46	69	1.67	Non Essential
Rv1455	8	0.20	7	0.19	Non Essential
Rv1456c	14	0.32	24	0.61	Essential
Rv1457c	82	2.24	84	2.55	Essential

Rv1458c	125	2.85	132	3.34	Non Essential
Rv1459c	271	3.28	263	3.53	Essential
Rv1460	234	6.24	110	3.25	Essential
Rv1461	3882	32.83	1647	15.44	Essential
Rv1462	1210	21.79	545	10.88	Essential
Rv1463	536	14.39	281	8.37	Essential
Rv1464	833	14.28	382	7.26	Essential
Rv1465	420	18.49	170	8.30	Essential
Rv1466	2279	141.08	1002	68.77	Essential
Rv1467c	113	1.33	125	1.63	Non Essential
Rv1468c	268	5.18	226	4.84	Non Essential
Rv1469	274	2.98	191	2.31	Essential
Rv1470	16	0.92	12	0.76	Non Essential
Rv1471	15	0.87	20	1.28	Non Essential
Rv1472	206	5.16	264	7.34	Non Essential
Rv1473	232	3.06	336	4.92	Non Essential
Rv1473A	122	13.72	151	18.83	Non Essential
Rv1474c	129	4.92	91	3.85	Non Essential
Rv1475c	661	5.02	706	5.94	Essential
Rv1476	155	5.95	160	6.80	Essential
Rv1477	1215	18.41	1372	23.04	Essential
Rv1478	215	6.37	260	8.54	Non Essential
Rv1479	1143	21.67	995	20.92	Essential
Rv1480	395	8.90	287	7.17	Essential
Rv1481	858	18.30	708	16.74	Non Essential
Rv1482c	6	0.15	6	0.17	Non Essential
Rv1483	676	19.54	776	24.87	Non Essential
Rv1484	3097	82.23	4345	127.91	Non Essential
Rv1485	188	3.91	192	4.42	Essential
Rv1486c	217	5.38	167	4.59	Non Essential
Rv1487	646	31.97	508	27.88	Non Essential
Rv1488	730	13.69	571	11.88	Non Essential
Rv1489	265	15.99	163	10.90	Non Essential
Rv1489A	45	4.20	46	4.76	Non Essential
Rv1490	18	0.30	16	0.29	Essential
Rv1491c	150	4.25	173	5.44	Non Essential
Rv1492	145	1.69	131	1.69	Non Essential
Rv1493	307	2.93	310	3.28	Non Essential
Rv1494	59	4.20	78	6.15	Non Essential
Rv1495	24	1.63	22	1.65	Non Essential
Rv1496	113	2.42	108	2.56	Non Essential
Rv1497	1758	29.30	1585	29.28	Non Essential
Rv1498c	111	3.86	137	5.29	Non Essential
Rv1498A	34	3.44	39	4.38	Non Essential
Rv1499	3	0.16	6	0.36	Non Essential
Rv1500	157	3.28	200	4.63	Non Essential
Rv1501	331	8.66	274	7.95	Non Essential
Rv1502	1176	28.10	1016	26.92	Non Essential
Rv1503c	63	2.47	50	2.17	Non Essential
Rv1504c	47	1.69	44	1.75	Non Essential

Rv1505c	199	6.43	144	5.16	Non Essential
Rv1506c	142	6.10	109	5.19	Non Essential
Rv1507c	372	11.50	393	13.47	Non Essential
Rv1507A	1	0.04	1	0.05	Non Essential
Rv1508c	196	2.34	212	2.81	Non Essential
Rv1508A	24	1.42	29	1.91	Non Essential
Rv1509	73	1.78	74	2.00	Non Essential
Rv1510	144	2.38	183	3.36	Non Essential
Rv1511	283	5.95	319	7.43	Non Essential
Rv1512	288	6.39	285	7.01	Non Essential
Rv1513	190	5.58	226	7.36	Non Essential
Rv1514c	41	1.12	52	1.57	Essential
Rv1515c	38	0.91	52	1.38	Non Essential
Rv1516c	21	0.45	20	0.47	Non Essential
Rv1517	43	1.21	60	1.87	Non Essential
Rv1518	18	0.40	39	0.97	Non Essential
Rv1519	41	3.27	20	1.77	Non Essential
Rv1520	234	4.83	170	3.89	Non Essential
Rv1521	1219	14.95	1046	14.23	Non Essential
Rv1522c	305	1.90	298	2.06	Non Essential
Rv1523	56	1.15	61	1.39	Non Essential
Rv1524	141	2.43	184	3.52	Essential
Rv1525	53	1.45	33	1.00	Non Essential
Rv1526c	29	0.49	21	0.39	Non Essential
Rv1527c	459	1.56	511	1.92	Non Essential
Rv1528c	1	0.04	3	0.14	Non Essential
Rv1529	124	1.52	148	2.01	Non Essential
Rv1530	49	0.95	36	0.78	Non Essential
Rv1531	51	1.94	42	1.77	Non Essential
Rv1532c	13	0.64	11	0.60	Non Essential
Rv1533	446	8.50	423	8.94	Non Essential
Rv1534	211	6.69	176	6.19	Non Essential
Rv1535	90	8.19	86	8.68	Non Essential
Rv1536	577	3.97	497	3.79	Non Essential
Rv1537	53	0.82	49	0.84	Non Essential
Rv1538c	84	1.84	93	2.26	Non Essential
Rv1539	546	19.29	570	22.33	Essential
Rv1540	243	5.64	210	5.40	Non Essential
Rv1541c	25	0.91	52	2.09	Non Essential
Rv1542c	1	0.05	4	0.23	Non Essential
Rv1543	350	7.33	296	6.88	Non Essential
Rv1544	286	7.65	220	6.52	Non Essential
Rv1545	103	9.75	68	7.13	Non Essential
Rv1546	88	4.39	96	5.30	Non Essential
Rv1547	1038	6.27	901	6.04	Essential
Rv1548c	64	0.68	74	0.87	Non Essential
Rv1549	21	0.86	30	1.36	Non Essential
Rv1550	203	2.54	191	2.65	Non Essential
Rv1551	80	0.92	63	0.80	Non Essential
Rv1552	62	0.76	35	0.48	Non Essential

Rv1553	11	0.32	10	0.32	Non Essential
Rv1554	13	0.73	6	0.38	Non Essential
Rv1555	17	0.97	11	0.69	Non Essential
Rv1556	19	0.67	24	0.94	Non Essential
Rv1557	50	0.90	57	1.14	Non Essential
Rv1558	217	10.45	179	9.56	Non Essential
Rv1559	500	8.33	553	10.22	Non Essential
Rv1560	91	8.97	129	14.09	Essential
Rv1561	24	1.28	36	2.12	Non Essential
Rv1562c	56	0.69	36	0.49	Non Essential
Rv1563c	231	2.16	220	2.28	Non Essential
Rv1564c	618	6.13	529	5.82	Non Essential
Rv1565c	235	2.31	214	2.33	Non Essential
Rv1566c	94	2.92	105	3.61	Non Essential
Rv1567c	20	1.51	11	0.92	Non Essential
Rv1568	64	1.05	83	1.51	Essential
Rv1569	246	4.56	251	5.15	Essential
Rv1570	102	3.22	97	3.40	Non Essential
Rv1571	64	2.70	62	2.90	Non Essential
Rv1572c	0	0.00	0	0.00	Non Essential
Rv1573	23	1.21	15	0.87	Non Essential
Rv1574	6	0.41	11	0.84	Non Essential
Rv1575	15	0.64	11	0.52	Non Essential
Rv1576c	213	3.22	146	2.45	Non Essential
Rv1577c	38	1.59	33	1.54	Non Essential
Rv1578c	9	0.41	14	0.71	Non Essential
Rv1579c	30	2.05	23	1.74	Non Essential
Rv1580c	10	0.79	7	0.61	Non Essential
Rv1581c	3	0.16	2	0.12	Non Essential
Rv1582c	27	0.41	12	0.20	Non Essential
Rv1583c	24	1.30	21	1.26	Non Essential
Rv1584c	13	1.26	16	1.72	Non Essential
Rv1585c	24	1.00	20	0.92	Non Essential
Rv1586c	93	1.42	75	1.27	Non Essential
Rv1587c	42	0.90	40	0.95	Non Essential
Rv1588c	33	1.06	41	1.46	Non Essential
Rv1589	509	10.42	523	11.87	Essential
Rv1590	124	11.14	141	14.05	Essential
Rv1591	107	3.46	101	3.62	Non Essential
Rv1592c	234	3.75	225	4.00	Non Essential
Rv1593c	127	3.84	107	3.59	Non Essential
Rv1594	1339	27.42	1639	37.21	Essential
Rv1595	1890	25.65	1775	26.70	Essential
Rv1596	2427	60.83	2294	63.75	Non Essential
Rv1597	365	10.34	420	13.20	Non Essential
Rv1598c	271	14.20	271	15.74	Non Essential
Rv1599	245	4.00	229	4.14	Essential
Rv1600	224	4.21	185	3.86	Essential
Rv1601	63	2.14	48	1.81	Essential
Rv1602	158	5.47	121	4.65	Essential

Rv1603	475	13.84	374	12.09	Essential
Rv1604	198	5.24	150	4.40	Non Essential
Rv1605	134	3.58	110	3.26	Essential
Rv1606	214	13.25	139	9.54	Essential
Rv1607	253	5.02	357	7.86	Non Essential
Rv1608c	89	4.12	73	3.75	Non Essential
Rv1609	627	8.69	681	10.46	Essential
Rv1610	332	10.09	323	10.88	Essential
Rv1611	2312	60.71	2085	60.70	Essential
Rv1612	2618	45.65	2248	43.46	Essential
Rv1613	1997	52.83	1870	54.85	Essential
Rv1614	1988	30.37	2082	35.27	Essential
Rv1615	265	12.94	272	14.72	Non Essential
Rv1616	93	5.02	79	4.73	Non Essential
Rv1617	494	7.48	556	9.34	Essential
Rv1618	487	11.60	548	14.47	Non Essential
Rv1619	226	3.34	185	3.03	Non Essential
Rv1620c	20	0.25	28	0.39	Non Essential
Rv1621c	89	1.21	81	1.22	Non Essential
Rv1622c	136	2.81	182	4.17	Essential
Rv1623c	53	0.78	78	1.27	Non Essential
Rv1624c	69	2.52	64	2.60	Non Essential
Rv1625c	251	4.05	203	3.63	Non Essential
Rv1626	2089	72.73	2169	83.72	Essential
Rv1627c	671	11.93	569	11.22	Non Essential
Rv1628c	153	6.69	125	6.06	Non Essential
Rv1629	230	1.82	278	2.44	Essential
Rv1630	1819	27.04	1722	28.38	Essential
Rv1631	1558	27.36	1457	28.37	Essential
Rv1632c	102	4.95	83	4.46	Non Essential
Rv1633	711	7.29	594	6.75	Non Essential
Rv1634	726	11.02	596	10.03	Non Essential
Rv1635c	85	1.09	78	1.11	Non Essential
Rv1636	555	27.09	536	29.01	Non Essential
Rv1637c	106	2.87	97	2.91	Non Essential
Rv1638	878	6.46	895	7.30	Non Essential
Rv1638A	91	7.61	81	7.51	Non Essential
Rv1639c	1677	24.52	1104	17.90	Non Essential
Rv1640c	234	1.43	217	1.47	Essential
Rv1641	2662	94.51	3150	124.00	Essential
Rv1642	1159	128.33	1326	162.78	Non Essential
Rv1643	740	40.86	714	43.71	Non Essential
Rv1644	920	25.27	858	26.13	Non Essential
Rv1645c	120	2.44	104	2.35	Non Essential
Rv1646	294	6.78	284	7.26	Non Essential
Rv1647	79	1.79	91	2.28	Non Essential
Rv1648	64	1.71	70	2.07	Non Essential
Rv1649	85	1.78	119	2.76	Essential
Rv1650	293	2.52	389	3.71	Essential
Rv1651c	508	3.60	442	3.47	Non Essential

Rv1652	32	0.65	17	0.38	Essential
Rv1653	97	1.72	83	1.63	Essential
Rv1654	88	2.14	90	2.42	Essential
Rv1655	96	1.72	88	1.74	Essential
Rv1656	115	2.68	74	1.91	Essential
Rv1657	410	17.20	333	15.49	Non Essential
Rv1658	567	10.18	530	10.55	Essential
Rv1659	227	3.45	222	3.74	Essential
Rv1660	128	2.59	95	2.13	Non Essential
Rv1661	1023	3.44	717	2.68	Essential
Rv1662	836	3.73	562	2.78	Essential
Rv1663	221	3.15	167	2.64	Essential
Rv1664	288	2.03	178	1.39	Non Essential
Rv1665	74	1.50	73	1.64	Non Essential
Rv1666c	72	1.20	69	1.27	Non Essential
Rv1667c	88	2.89	104	3.79	Non Essential
Rv1668c	137	2.63	148	3.15	Non Essential
Rv1669	6	0.36	12	0.79	Non Essential
Rv1670	24	1.49	25	1.72	Non Essential
Rv1671	30	1.64	28	1.70	Non Essential
Rv1672c	27	0.44	52	0.93	Non Essential
Rv1673c	13	0.30	10	0.26	Non Essential
Rv1674c	7	0.23	13	0.47	Non Essential
Rv1675c	6	0.18	7	0.23	Non Essential
Rv1676	287	8.76	314	10.62	Non Essential
Rv1677	186	7.29	221	9.60	Non Essential
Rv1678	738	17.57	681	17.98	Non Essential
Rv1679	437	8.37	427	9.07	Non Essential
Rv1680	78	2.03	65	1.88	Non Essential
Rv1681	86	1.86	75	1.80	Non Essential
Rv1682	41	0.96	28	0.73	Non Essential
Rv1683	1157	8.29	1385	11.00	Essential
Rv1684	37	3.55	41	4.36	Non Essential
Rv1685c	21	0.72	33	1.26	Non Essential
Rv1686c	21	0.66	14	0.49	Non Essential
Rv1687c	13	0.36	11	0.34	Non Essential
Rv1688	5	0.18	17	0.66	Non Essential
Rv1689	166	2.80	211	3.94	Essential
Rv1690	194	10.88	275	17.10	Non Essential
Rv1691	409	11.68	498	15.77	Non Essential
Rv1692	298	6.03	388	8.71	Non Essential
Rv1693	113	13.79	118	15.97	Non Essential
Rv1694	173	4.61	191	5.64	Non Essential
Rv1695	400	9.31	380	9.80	Essential
Rv1696	564	6.87	452	6.11	Essential
Rv1697	1267	23.04	1115	22.48	Essential
Rv1698	531	12.08	479	12.08	Non Essential
Rv1699	409	4.99	394	5.33	Essential
Rv1700	895	30.86	795	30.39	Non Essential
Rv1701	629	14.45	450	11.46	Essential

Rv1702c	53	0.83	36	0.63	Non Essential
Rv1703c	273	9.94	361	14.57	Non Essential
Rv1704c	452	5.81	630	8.98	Non Essential
Rv1705c	15	0.28	11	0.23	Non Essential
Rv1706c	69	1.25	55	1.11	Non Essential
Rv1706A	0	0.00	0	0.00	Non Essential
Rv1707	219	3.22	181	2.95	Non Essential
Rv1708	921	20.69	1045	26.03	Essential
Rv1709	1201	30.86	1369	39.00	Non Essential
Rv1710	212	6.55	191	6.55	Essential
Rv1711	932	26.20	776	24.19	Essential
Rv1712	637	19.77	549	18.89	Essential
Rv1713	972	15.01	872	14.93	Essential
Rv1714	57	1.51	62	1.82	Essential
Rv1715	64	1.50	54	1.41	Essential
Rv1716	118	3.05	106	3.04	Non Essential
Rv1717	42	2.58	39	2.65	Non Essential
Rv1718	195	5.12	155	4.51	Non Essential
Rv1719	207	5.71	191	5.84	Non Essential
Rv1720c	7	0.39	5	0.31	Non Essential
Rv1721c	20	1.89	16	1.68	Non Essential
Rv1722	130	1.88	135	2.17	Non Essential
Rv1723	43	0.74	33	0.63	Non Essential
Rv1724c	90	4.61	88	5.00	Non Essential
Rv1725c	28	0.85	15	0.50	Non Essential
Rv1726	4	0.06	2	0.03	Non Essential
Rv1727	0	0.00	3	0.13	Non Essential
Rv1728c	106	2.96	104	3.22	Non Essential
Rv1729c	44	1.01	55	1.40	Non Essential
Rv1730c	75	1.04	107	1.64	Essential
Rv1731	128	1.77	153	2.34	Non Essential
Rv1732c	53	2.08	43	1.87	Non Essential
Rv1733c	19	0.65	51	1.92	Non Essential
Rv1734c	1	0.09	1	0.10	Non Essential
Rv1735c	2	0.09	4	0.19	Non Essential
Rv1736c	32	0.35	58	0.71	Non Essential
Rv1737c	21	0.38	67	1.34	Non Essential
Rv1738	469	35.47	888	74.47	Essential
Rv1739c	17	0.22	12	0.17	Non Essential
Rv1740	206	20.87	179	20.11	Non Essential
Rv1741	66	5.72	62	5.95	Non Essential
Rv1742	92	2.68	68	2.20	Non Essential
Rv1743	395	4.99	422	5.91	Non Essential
Rv1744c	2	0.11	7	0.42	Non Essential
Rv1745c	0	0.00	2	0.08	Non Essential
Rv1746	197	2.96	155	2.58	Non Essential
Rv1747	941	7.78	927	8.50	Non Essential
Rv1748	37	1.09	39	1.27	Non Essential
Rv1749c	109	4.20	151	6.46	Non Essential
Rv1750c	134	1.80	115	1.71	Non Essential

Rv1751	933	14.50	1013	17.46	Non Essential
Rv1752	213	10.19	230	12.20	Non Essential
Rv1753c	186	1.26	164	1.24	Essential
Rv1754c	135	1.71	152	2.14	Non Essential
Rv1755c	1	0.03	0	0.00	Non Essential
Rv1756c	11	0.24	11	0.27	Non Essential
Rv1757c	19	1.25	14	1.02	Non Essential
Rv1758	4	0.16	0	0.00	Non Essential
Rv1759c	82	0.64	90	0.78	Non Essential
Rv1760	428	6.10	338	5.34	Non Essential
Rv1761c	14	0.79	12	0.75	Non Essential
Rv1762c	54	1.47	77	2.33	Non Essential
Rv1763	9	0.59	15	1.10	Non Essential
Rv1764	13	0.28	11	0.27	Non Essential
Rv1765c	169	3.31	201	4.36	Non Essential
Rv1765A	3	0.30	0	0.00	Non Essential
Rv1766	37	2.95	40	3.54	Non Essential
Rv1767	16	0.96	17	1.13	Non Essential
Rv1768	1643	19.02	1523	19.54	Non Essential
Rv1769	646	11.15	550	10.53	Non Essential
Rv1770	576	9.62	624	11.56	Non Essential
Rv1771	265	4.43	249	4.61	Non Essential
Rv1772	340	23.48	283	21.67	Non Essential
Rv1773c	15	0.43	19	0.61	Non Essential
Rv1774	168	2.69	172	3.06	Non Essential
Rv1775	547	14.36	490	14.27	Non Essential
Rv1776c	15	0.58	17	0.72	Non Essential
Rv1777	10	0.16	20	0.37	Non Essential
Rv1778c	64	3.06	91	4.83	Non Essential
Rv1779c	171	2.05	205	2.72	Non Essential
Rv1780	79	3.01	58	2.45	Non Essential
Rv1781c	32	0.32	26	0.28	Non Essential
Rv1782	1189	16.80	1054	16.51	Non Essential
Rv1783	819	13.46	763	13.90	Non Essential
Rv1784	3890	29.86	3530	30.05	Non Essential
Rv1785c	66	1.20	86	1.73	Non Essential
Rv1786	64	6.77	65	7.63	Non Essential
Rv1787	47	0.92	32	0.69	Non Essential
Rv1788	78	5.60	77	6.13	Non Essential
Rv1789	389	7.08	358	7.22	Non Essential
Rv1790	19	0.39	7	0.16	Non Essential
Rv1791	366	26.29	435	34.65	Non Essential
Rv1792	1112	80.70	1042	83.84	
Rv1793	441	33.36	428	35.89	Non Essential
Rv1794	807	19.22	539	14.23	Non Essential
Rv1795	782	11.12	687	10.83	Non Essential
Rv1796	3090	37.78	2347	31.81	Non Essential
Rv1797	1114	19.61	783	15.29	Non Essential
Rv1798	2582	30.27	1687	21.93	Non Essential
Rv1799	54	6.07	39	4.86	Non Essential

Rv1800	92	1.00	90	1.09	Non Essential
Rv1801	18	0.30	23	0.43	Non Essential
Rv1802	23	0.36	27	0.46	Non Essential
Rv1803c	328	3.67	266	3.30	Non Essential
Rv1804c	5	0.33	4	0.29	Non Essential
Rv1805c	0	0.00	0	0.00	Non Essential
Rv1806	4	0.29	5	0.40	Non Essential
Rv1807	21	0.38	24	0.48	Essential
Rv1808	146	2.55	124	2.40	Non Essential
Rv1809	152	2.32	113	1.91	Non Essential
Rv1810	117	7.06	83	5.55	Non Essential
Rv1811	153	4.67	123	4.16	Non Essential
Rv1812c	207	3.70	366	7.25	Non Essential
Rv1813c	35	1.74	82	4.53	Non Essential
Rv1814	123	2.93	161	4.25	Non Essential
Rv1815	285	9.21	344	12.32	Non Essential
Rv1816	296	9.03	281	9.51	Non Essential
Rv1817	83	1.22	75	1.22	Non Essential
Rv1818c	388	5.57	345	5.49	Non Essential
Rv1819c	107	1.20	129	1.60	Non Essential
Rv1820	309	4.04	284	4.12	Non Essential
Rv1821	1742	15.42	1701	16.70	Essential
Rv1822	459	15.67	420	15.90	Non Essential
Rv1823	193	4.49	196	5.06	Non Essential
Rv1824	112	6.59	127	8.29	Non Essential
Rv1825	296	7.24	245	6.65	Non Essential
Rv1826	197	10.47	212	12.50	Non Essential
Rv1827	2071	91.16	1856	90.58	Non Essential
Rv1828	388	11.22	327	10.48	Essential
Rv1829	278	12.09	219	10.56	Non Essential
Rv1830	363	11.52	314	11.05	Non Essential
Rv1831	211	17.64	172	15.94	Non Essential
Rv1832	638	4.85	511	4.31	Essential
Rv1833c	18	0.45	20	0.55	Non Essential
Rv1834	20	0.50	15	0.41	Non Essential
Rv1835c	187	2.13	146	1.84	Non Essential
Rv1836c	1246	13.17	1260	14.76	Non Essential
Rv1837c	3402	32.84	3842	41.12	Non Essential
Rv1838c	55	2.99	35	2.11	Non Essential
Rv1839c	56	4.57	48	4.35	Non Essential
Rv1840c	315	4.37	271	4.17	Non Essential
Rv1841c	618	12.80	406	9.32	Non Essential
Rv1842c	668	10.50	480	8.36	Non Essential
Rv1843c	746	11.14	665	11.01	Non Essential
Rv1844c	491	7.24	426	6.96	Non Essential
Rv1845c	179	4.05	200	5.01	Non Essential
Rv1846c	2229	115.10	3064	175.41	Non Essential
Rv1847	63	3.21	22	1.24	Non Essential
Rv1848	13	0.92	16	1.26	Non Essential
Rv1849	73	4.99	74	5.61	Non Essential

Rv1850	157	1.95	145	1.99	Essential
Rv1851	32	1.08	17	0.64	Non Essential
Rv1852	45	1.43	41	1.45	Non Essential
Rv1853	117	4.01	104	3.96	Non Essential
Rv1854c	232	3.58	222	3.80	Non Essential
Rv1855c	78	1.82	83	2.14	Non Essential
Rv1856c	93	2.95	88	3.10	Non Essential
Rv1857	146	4.00	112	3.40	Non Essential
Rv1858	26	0.70	16	0.48	Non Essential
Rv1859	143	2.77	132	2.83	Non Essential
Rv1860	102	2.24	101	2.46	Non Essential
Rv1861	107	7.54	89	6.95	Non Essential
Rv1862	59	1.22	54	1.24	Non Essential
Rv1863c	573	15.98	528	16.33	Non Essential
Rv1864c	104	2.96	107	3.38	Non Essential
Rv1865c	95	2.37	107	2.96	Non Essential
Rv1866	73	0.67	89	0.91	Non Essential
Rv1867	81	1.17	122	1.96	Non Essential
Rv1868	106	1.08	105	1.19	Non Essential
Rv1869c	1116	19.41	834	16.08	Non Essential
Rv1870c	678	22.94	460	17.25	Non Essential
Rv1871c	1570	86.69	1156	70.77	Non Essential
Rv1872c	4709	81.31	3275	62.70	Non Essential
Rv1873	8	0.39	7	0.38	Non Essential
Rv1874	58	1.82	69	2.40	Non Essential
Rv1875	70	3.39	147	7.90	Non Essential
Rv1876	136	6.10	234	11.63	Non Essential
Rv1877	181	1.88	255	2.94	Non Essential
Rv1878	77	1.22	84	1.48	Non Essential
Rv1879	18	0.34	18	0.38	Non Essential
Rv1880c	625	10.20	541	9.79	Non Essential
Rv1881c	217	11.05	222	12.53	Non Essential
Rv1882c	826	21.30	615	17.58	Non Essential
Rv1883c	2146	99.99	1846	95.37	Non Essential
Rv1884c	2746	111.29	2892	129.95	Non Essential
Rv1885c	96	3.44	118	4.69	Non Essential
Rv1886c	569	12.51	936	22.82	Non Essential
Rv1887	1048	19.71	1005	20.96	Non Essential
Rv1888c	8	0.31	11	0.47	Non Essential
Rv1888A	4	0.50	7	0.96	Non Essential
Rv1889c	43	2.59	40	2.68	Non Essential
Rv1890c	36	1.27	36	1.40	Non Essential
Rv1891	155	8.18	175	10.24	Non Essential
Rv1892	69	4.77	58	4.44	Non Essential
Rv1893	185	18.23	151	16.50	Non Essential
Rv1894c	180	3.42	128	2.70	Non Essential
Rv1895	19	0.35	31	0.64	Non Essential
Rv1896c	97	2.29	108	2.82	Non Essential
Rv1897c	33	1.64	32	1.77	Non Essential
Rv1898	54	3.77	47	3.63	Non Essential

Rv1899c	1023	21.31	973	22.48	Non Essential
Rv1900c	527	8.16	441	7.57	Non Essential
Rv1901	220	3.66	248	4.57	Non Essential
Rv1902c	28	0.47	30	0.56	Non Essential
Rv1903	22	1.17	22	1.30	Non Essential
Rv1904	176	8.77	125	6.91	Non Essential
Rv1905c	298	6.65	257	6.36	Non Essential
Rv1906c	401	18.33	401	20.32	Non Essential
Rv1907c	59	1.96	43	1.58	Non Essential
Rv1908c	1099	10.62	1340	14.36	Non Essential
Rv1909c	46	2.19	47	2.48	Non Essential
Rv1910c	32	1.16	34	1.37	Non Essential
Rv1911c	251	8.91	286	11.26	Non Essential
Rv1912c	197	4.21	180	4.27	Non Essential
Rv1913	9	0.26	17	0.54	Non Essential
Rv1914c	96	5.07	98	5.73	Non Essential
Rv1915	484	9.43	758	16.37	Non Essential
Rv1916	148	2.66	204	4.06	Non Essential
Rv1917c	58	0.28	38	0.21	Non Essential
Rv1918c	657	4.76	678	5.45	Non Essential
Rv1919c	311	14.40	332	17.04	Non Essential
Rv1920	28	0.70	33	0.91	Non Essential
Rv1921c	37	0.63	43	0.81	Non Essential
Rv1922	78	1.50	91	1.94	Non Essential
Rv1923	389	6.24	444	7.89	Non Essential
Rv1924c	39	2.20	47	2.95	Non Essential
Rv1925	2211	25.51	2369	30.30	Non Essential
Rv1926c	1823	81.75	3810	189.43	Non Essential
Rv1927	41	1.14	35	1.08	Non Essential
Rv1928c	49	1.37	63	1.96	Non Essential
Rv1929c	243	8.11	348	12.87	Non Essential
Rv1930c	3	0.12	6	0.27	Essential
Rv1931c	11	0.30	23	0.70	Essential
Rv1932	226	9.77	259	12.41	Non Essential
Rv1933c	7	0.14	6	0.13	Non Essential
Rv1934c	12	0.21	5	0.10	Non Essential
Rv1935c	9	0.20	5	0.12	Non Essential
Rv1936	101	1.96	107	2.30	Non Essential
Rv1937	76	0.65	65	0.61	Non Essential
Rv1938	26	0.52	38	0.85	Non Essential
Rv1939	12	0.50	12	0.55	Essential
Rv1940	41	0.83	48	1.08	Non Essential
Rv1941	35	0.98	30	0.93	Non Essential
Rv1942c	76	4.96	52	3.76	Non Essential
Rv1943c	177	10.08	126	7.96	Non Essential
Rv1944c	15	0.55	20	0.81	Non Essential
Rv1945	96	1.51	94	1.64	Non Essential
Rv1946c	3	0.14	3	0.16	Non Essential
Rv1947	127	6.80	166	9.86	Non Essential
Rv1948c	14	0.86	15	1.02	Non Essential

Rv1949c	18	0.40	13	0.32	Non Essential
Rv1950c	34	3.82	27	3.37	Non Essential
Rv1951c	20	1.45	16	1.29	Non Essential
Rv1952	21	2.10	21	2.33	Non Essential
Rv1953	1404	96.97	975	74.66	Non Essential
Rv1954c	24	0.99	7	0.32	Non Essential
Rv1954A	23731	1687.92	16611	1309.95	
Rv1955	241	13.73	226	14.28	Non Essential
Rv1956	0	0.00	0	0.00	Non Essential
Rv1957	326	12.85	198	8.65	Non Essential
Rv1958c	0	0.00	2	0.08	Non Essential
Rv1959c	59	4.28	34	2.74	Non Essential
Rv1960c	16	1.37	11	1.04	Non Essential
Rv1961	23	1.00	18	0.87	Non Essential
Rv1962c	25	1.32	21	1.23	Non Essential
Rv1962A	29	2.29	31	2.71	
Rv1963c	83	1.46	78	1.52	Essential
Rv1964	13	0.35	4	0.12	Non Essential
Rv1965	22	0.58	8	0.23	Non Essential
Rv1966	4	0.07	7	0.13	Non Essential
Rv1967	3	0.06	5	0.12	Non Essential
Rv1968	6	0.10	4	0.08	Non Essential
Rv1969	4	0.07	2	0.04	Non Essential
Rv1970	2	0.04	2	0.04	Non Essential
Rv1971	17	0.28	10	0.18	Non Essential
Rv1972	8	0.30	11	0.46	Non Essential
Rv1973	6	0.27	2	0.10	Non Essential
Rv1974	3	0.17	3	0.19	Essential
Rv1975	1	0.03	4	0.14	Non Essential
Rv1976c	256	13.51	326	19.08	Non Essential
Rv1977	443	9.10	345	7.86	Non Essential
Rv1978	54	1.37	43	1.21	Non Essential
Rv1979c	247	3.67	205	3.38	Non Essential
Rv1980c	483	15.12	611	21.21	Non Essential
Rv1981c	168	3.73	94	2.31	Non Essential
Rv1982c	152	7.79	181	10.29	Non Essential
Rv1982A	41	3.39	30	2.75	
Rv1983	460	5.90	382	5.43	Non Essential
Rv1984c	220	7.24	285	10.39	Non Essential
Rv1985c	23	0.54	43	1.12	Non Essential
Rv1986	96	3.44	103	4.10	Non Essential
Rv1987	319	16.01	379	21.09	Non Essential
Rv1988	847	33.75	827	36.54	Non Essential
Rv1989c	82	3.15	46	1.96	Non Essential
Rv1990c	145	9.13	108	7.54	Non Essential
Rv1990A	35	2.24	12	0.85	Non Essential
Rv1991c	17	1.06	10	0.69	Non Essential
Rv1991A	91	7.88	83	7.97	
Rv1992c	151	1.40	125	1.29	Non Essential
Rv1993c	154	12.16	148	12.96	Non Essential

Rv1994c	355	21.42	266	17.79	Non Essential
Rv1995	7	0.20	2	0.06	Non Essential
Rv1996	96	2.16	148	3.70	Non Essential
Rv1997	42	0.33	52	0.46	Non Essential
Rv1998c	19	0.53	23	0.71	Non Essential
Rv1999c	11	0.18	16	0.29	Non Essential
Rv2000	14	0.19	17	0.25	Non Essential
Rv2001	9	0.26	10	0.32	Non Essential
Rv2002	42	1.15	57	1.74	Non Essential
Rv2003c	24	0.60	20	0.56	Non Essential
Rv2004c	208	2.99	147	2.34	Essential
Rv2005c	488	11.82	479	12.86	Non Essential
Rv2006	165	0.89	157	0.94	Non Essential
Rv2007c	9	0.56	47	3.25	Non Essential
Rv2008c	14	0.23	13	0.23	Non Essential
Rv2009	442	39.23	527	51.86	Non Essential
Rv2010	136	7.34	142	8.50	Non Essential
Rv2011c	12	0.60	23	1.27	Non Essential
Rv2012	11	0.48	8	0.39	Non Essential
Rv2013	1	0.04	1	0.05	Essential
Rv2014	0	0.00	0	0.00	Non Essential
Rv2015c	176	3.01	200	3.79	Non Essential
Rv2016	91	3.40	60	2.49	Non Essential
Rv2017	77	1.59	71	1.63	Non Essential
Rv2018	105	3.14	106	3.51	Non Essential
Rv2019	14	0.72	13	0.74	Non Essential
Rv2020c	18	1.29	28	2.23	Non Essential
Rv2021c	78	5.49	62	4.84	Non Essential
Rv2022c	135	4.79	123	4.84	Non Essential
Rv2023c	29	1.74	35	2.32	Non Essential
Rv2023A	2664	124.94	2081	108.21	
Rv2024c	253	3.51	228	3.51	Non Essential
Rv2025c	8	0.17	8	0.19	Non Essential
Rv2026c	79	1.92	80	2.16	Essential
Rv2027c	73	0.91	74	1.02	Non Essential
Rv2028c	13	0.33	12	0.34	Non Essential
Rv2029c	10	0.21	9	0.21	Non Essential
Rv2030c	34	0.36	103	1.20	Non Essential
Rv2031c	145	7.18	317	17.40	Non Essential
Rv2032	15	0.32	35	0.84	Non Essential
Rv2033c	185	4.72	266	7.52	Non Essential
Rv2034	11	0.73	17	1.25	Non Essential
Rv2035	40	1.76	34	1.66	Non Essential
Rv2036	37	1.24	32	1.19	Non Essential
Rv2037c	67	1.48	68	1.66	Non Essential
Rv2038c	124	2.48	105	2.33	Essential
Rv2039c	39	0.99	31	0.88	Non Essential
Rv2040c	51	1.21	47	1.24	Essential
Rv2041c	37	0.60	32	0.58	Essential
Rv2042c	68	1.83	55	1.64	Non Essential

Rv2043c	209	8.02	242	10.29	Non Essential
Rv2044c	8	0.54	4	0.30	Non Essential
Rv2045c	111	1.55	75	1.16	Non Essential
Rv2046	96	3.14	134	4.86	Non Essential
Rv2047c	398	3.33	290	2.69	Non Essential
Rv2048c	5231	9.02	4470	8.55	Essential
Rv2049c	26	2.49	22	2.34	Non Essential
Rv2050	47	3.01	69	4.91	Essential
Rv2051c	1032	8.45	890	8.08	Non Essential
Rv2052c	813	10.89	644	9.56	Non Essential
Rv2053c	283	11.54	284	12.83	Non Essential
Rv2054	62	1.87	61	2.04	Non Essential
Rv2055c	51	4.12	44	3.94	Non Essential
Rv2056c	66	4.65	25	1.95	Non Essential
Rv2057c	57	7.47	18	2.61	Non Essential
Rv2058c	25	2.28	9	0.91	Non Essential
Rv2059	104	1.46	60	0.93	Non Essential
Rv2060	27	1.45	10	0.59	Non Essential
Rv2061c	222	11.80	249	14.68	Non Essential
Rv2062c	807	4.84	943	6.27	Non Essential
Rv2063	68	6.27	97	9.91	Non Essential
Rv2063A	42	2.20	36	2.09	
Rv2064	134	2.64	128	2.79	Non Essential
Rv2065	94	3.23	63	2.40	Non Essential
Rv2066	138	1.94	145	2.26	Non Essential
Rv2067c	148	2.60	172	3.35	Non Essential
Rv2068c	382	8.89	344	8.88	Non Essential
Rv2069	17	0.66	36	1.54	Non Essential
Rv2070c	61	1.79	61	1.98	Non Essential
Rv2071c	13	0.37	18	0.57	Non Essential
Rv2072c	41	0.75	59	1.20	Essential
Rv2073c	18	0.52	19	0.60	Non Essential
Rv2074	135	7.02	211	12.17	Non Essential
Rv2075c	199	2.92	181	2.95	Non Essential
Rv2076c	64	5.48	51	4.84	Non Essential
Rv2077c	231	5.11	236	5.79	Non Essential
Rv2077A	8	0.57	4	0.32	Non Essential
Rv2078	35	2.39	53	4.02	Non Essential
Rv2079	67	0.73	72	0.87	Non Essential
Rv2080	54	2.06	64	2.71	Non Essential
Rv2081c	26	1.27	26	1.41	Non Essential
Rv2082	121	1.20	96	1.06	Non Essential
Rv2083	227	5.17	155	3.91	Non Essential
Rv2084	609	11.52	517	10.84	Non Essential
Rv2085	89	6.27	63	4.92	Non Essential
Rv2086	3	0.11	3	0.12	Non Essential
Rv2087	0	0.00	0	0.00	Non Essential
Rv2088	53	0.64	69	0.93	Non Essential
Rv2089c	17	0.32	21	0.44	Non Essential
Rv2090	23	0.42	38	0.77	Non Essential

Rv2091c	498	14.57	504	16.35	Non Essential
Rv2092c	1013	8.00	668	5.85	Non Essential
Rv2093c	215	4.99	148	3.81	Essential
Rv2094c	7620	652.12	6423	609.44	Non Essential
Rv2095c	181	4.09	128	3.21	Non Essential
Rv2096c	372	8.01	301	7.18	Non Essential
Rv2097c	673	10.65	684	12.00	Non Essential
Rv2098c	103	1.70	123	2.25	
Rv2099c	3	0.37	5	0.69	
Rv2100	28	0.36	37	0.53	Non Essential
Rv2101	1026	7.25	1085	8.50	Non Essential
Rv2102	161	4.83	169	5.62	Non Essential
Rv2103c	45	2.23	43	2.36	Non Essential
Rv2104c	85	7.19	89	8.34	Non Essential
Rv2105	13	0.86	10	0.73	Non Essential
Rv2106	9	0.20	12	0.29	Non Essential
Rv2107	3	0.22	2	0.16	Non Essential
Rv2108	54	1.59	71	2.31	Non Essential
Rv2109c	1388	39.97	894	28.54	Essential
Rv2110c	1285	31.55	1019	27.74	Essential
Rv2111c	278	30.78	293	35.97	Essential
Rv2112c	752	9.71	751	10.75	Non Essential
Rv2113	73	1.31	77	1.54	Non Essential
Rv2114	42	1.45	57	2.18	Non Essential
Rv2115c	1151	13.52	831	10.82	Non Essential
Rv2116	11	0.42	14	0.59	Non Essential
Rv2117	5	0.37	7	0.57	Non Essential
Rv2118c	272	6.94	316	8.94	Non Essential
Rv2119	49	1.26	56	1.60	Non Essential
Rv2120c	76	3.39	72	3.56	Non Essential
Rv2121c	137	3.45	32	0.89	Essential
Rv2122c	105	8.03	19	1.61	Essential
Rv2123	338	5.11	9	0.15	Non Essential
Rv2124c	2101	12.61	1659	11.04	Non Essential
Rv2125	285	6.97	332	9.01	Non Essential
Rv2126c	15	0.42	19	0.59	Non Essential
Rv2127	2386	34.89	2367	38.37	Non Essential
Rv2128	100	10.58	115	13.49	Non Essential
Rv2129c	69	1.68	64	1.73	Non Essential
Rv2130c	292	5.04	303	5.80	Essential
Rv2131c	223	5.97	222	6.58	Non Essential
Rv2132	101	9.43	92	9.53	Non Essential
Rv2133c	148	4.03	107	3.23	Non Essential
Rv2134c	553	20.24	405	16.43	Non Essential
Rv2135c	666	20.15	504	16.91	Non Essential
Rv2136c	439	11.36	295	8.46	Non Essential
Rv2137c	854	44.42	559	32.23	Non Essential
Rv2138	107	2.14	100	2.21	Essential
Rv2139	178	3.56	168	3.73	Non Essential
Rv2140c	75	3.04	90	4.04	Non Essential

Rv2141c	256	4.09	319	5.64	Non Essential
Rv2142c	40	2.71	35	2.63	Non Essential
Rv2142A	42	4.20	48	5.32	
Rv2143	204	4.14	198	4.46	Non Essential
Rv2144c	1258	75.91	1324	88.57	Non Essential
Rv2145c	2896	79.55	2585	78.73	Essential
Rv2146c	1630	120.73	1302	106.92	Non Essential
Rv2147c	1106	32.77	998	32.78	Non Essential
Rv2148c	385	10.66	295	9.05	Non Essential
Rv2149c	326	9.31	261	8.27	Non Essential
Rv2150c	2090	39.42	1632	34.12	Essential
Rv2151c	447	10.17	410	10.34	Essential
Rv2152c	679	9.83	576	9.24	Essential
Rv2153c	353	6.15	333	6.44	Essential
Rv2154c	1393	19.01	1266	19.16	Essential
Rv2155c	576	8.47	530	8.65	Essential
Rv2156c	614	12.22	467	10.31	Essential
Rv2157c	334	4.68	258	4.01	Essential
Rv2158c	1022	13.66	953	14.12	Non Essential
Rv2159c	498	10.35	368	8.48	Non Essential
Rv2160A	508	17.18	352	13.20	Non Essential
Rv2160c	317	19.97	215	15.02	Non Essential
Rv2161c	5907	146.52	4180	114.95	Non Essential
Rv2162c	4746	63.80	4213	62.79	Non Essential
Rv2163c	698	7.35	564	6.59	Essential
Rv2164c	237	4.41	221	4.56	Essential
Rv2165c	490	8.84	433	8.67	Essential
Rv2166c	181	9.02	162	8.95	Essential
Rv2167c	9	0.20	12	0.29	Non Essential
Rv2168c	18	1.19	10	0.73	Non Essential
Rv2169c	200	10.63	230	13.56	Non Essential
Rv2170	7	0.24	5	0.19	Non Essential
Rv2171	108	3.40	166	5.79	Non Essential
Rv2172c	272	6.46	212	5.58	Non Essential
Rv2173	122	2.48	109	2.45	Non Essential
Rv2174	260	3.60	189	2.90	Essential
Rv2175c	95	4.64	96	5.20	Non Essential
Rv2176	54	0.97	68	1.35	Non Essential
Rv2177c	19	0.61	15	0.54	Non Essential
Rv2178c	621	9.61	631	10.83	Essential
Rv2179c	30	1.27	28	1.32	Essential
Rv2180c	34	0.82	45	1.21	Non Essential
Rv2181	205	3.43	217	4.03	Non Essential
Rv2182c	230	6.65	204	6.54	Essential
Rv2183c	101	5.49	101	6.09	Non Essential
Rv2184c	352	6.64	384	8.03	Non Essential
Rv2185c	1171	57.96	1275	69.97	Non Essential
Rv2186c	194	10.71	220	13.47	Non Essential
Rv2187	434	5.17	437	5.78	Non Essential
Rv2188c	62	1.15	79	1.63	Essential

Rv2189c	87	2.42	80	2.46	Non Essential
Rv2190c	1303	24.19	1372	28.24	Non Essential
Rv2191	23	0.26	37	0.45	Non Essential
Rv2192c	138	2.67	185	3.96	Essential
Rv2193	335	11.78	576	22.45	Essential
Rv2194	567	14.46	829	23.45	Essential
Rv2195	1115	18.58	1553	28.69	Essential
Rv2196	1020	13.29	1344	19.41	Essential
Rv2197c	300	10.01	322	11.91	Non Essential
Rv2198c	438	10.47	519	13.75	Non Essential
Rv2199c	243	12.46	424	24.10	Non Essential
Rv2200c	942	18.55	1323	28.88	Essential
Rv2201	72	0.79	94	1.14	Essential
Rv2202c	4398	96.99	3430	83.87	Non Essential
Rv2203	20	0.62	34	1.17	Non Essential
Rv2204c	1772	106.92	1718	114.93	Non Essential
Rv2205c	110	2.20	95	2.10	Non Essential
Rv2206	97	2.93	100	3.35	Non Essential
Rv2207	183	3.62	195	4.28	Non Essential
Rv2208	111	3.18	123	3.91	Non Essential
Rv2209	48	0.67	50	0.77	Non Essential
Rv2210c	518	10.06	563	12.12	Essential
Rv2211c	460	8.68	592	12.38	Essential
Rv2212	44	0.83	56	1.17	Non Essential
Rv2213	339	4.71	522	8.04	Non Essential
Rv2214c	325	3.93	326	4.37	Non Essential
Rv2215	1134	14.67	1174	16.83	Essential
Rv2216	424	10.06	461	12.13	Non Essential
Rv2217	207	6.43	252	8.67	Essential
Rv2218	221	5.08	226	5.76	Essential
Rv2219	346	9.88	314	9.94	Essential
Rv2219A	21	1.07	38	2.14	Non Essential
Rv2220	3221	48.18	4155	68.91	Essential
Rv2221c	2274	16.37	2268	18.10	Essential
Rv2222c	826	13.24	908	16.14	Non Essential
Rv2223c	327	4.50	314	4.79	Non Essential
Rv2224c	526	7.23	573	8.74	Essential
Rv2225	189	4.80	118	3.33	Essential
Rv2226	143	1.99	155	2.40	Non Essential
Rv2227	12	0.37	12	0.41	Non Essential
Rv2228c	153	3.00	166	3.61	Non Essential
Rv2229c	231	6.73	257	8.30	Essential
Rv2230c	241	4.55	228	4.77	Non Essential
Rv2231c	164	3.22	186	4.05	Essential
Rv2231A	0	0.00	0	0.00	
Rv2231B	10	1.22	16	2.17	
Rv2232	105	2.58	118	3.21	Non Essential
Rv2234	38	1.66	33	1.60	Non Essential
Rv2235	384	10.12	296	8.65	Essential
Rv2236c	103	2.35	125	3.16	Non Essential

Rv2237	91	2.55	128	3.97	Non Essential
Rv2238c	429	19.99	411	21.23	Non Essential
Rv2239c	1129	50.95	1149	57.49	Non Essential
Rv2240c	131	4.77	149	6.01	Non Essential
Rv2241	1155	9.17	1328	11.69	Essential
Rv2242	142	2.45	129	2.47	Essential
Rv2243	458	10.83	433	11.36	Non Essential
Rv2244	2286	141.51	1859	127.59	Essential
Rv2245	7688	132.11	5294	100.86	Essential
Rv2246	5265	85.94	3010	54.47	Essential
Rv2247	11717	177.12	6206	104.01	Essential
Rv2248	80	2.11	59	1.72	Non Essential
Rv2249c	139	1.93	46	0.71	Non Essential
Rv2250c	13	0.49	7	0.29	Non Essential
Rv2250A	43	2.20	13	0.74	Non Essential
Rv2251	221	3.33	69	1.15	Essential
Rv2252	143	3.31	70	1.79	Non Essential
Rv2253	20	0.85	14	0.66	Non Essential
Rv2254c	11	0.52	8	0.42	Non Essential
Rv2255c	145	16.05	107	13.14	Non Essential
Rv2256c	10624	428.16	8879	396.74	Non Essential
Rv2257c	255	6.70	294	8.56	Non Essential
Rv2258c	1074	21.74	1293	29.02	Non Essential
Rv2259	487	9.64	510	11.19	Non Essential
Rv2260	207	7.00	261	9.79	Essential
Rv2261c	24	1.22	12	0.68	Non Essential
Rv2262c	101	2.01	78	1.72	Non Essential
Rv2263	51	1.15	58	1.45	Non Essential
Rv2264c	74	0.89	77	1.03	Non Essential
Rv2265	25	0.44	23	0.45	Non Essential
Rv2266	70	1.17	73	1.35	Non Essential
Rv2267c	26	0.48	19	0.39	Non Essential
Rv2268c	6	0.09	17	0.28	Essential
Rv2269c	0	0.00	2	0.14	Non Essential
Rv2270	2	0.08	7	0.32	Non Essential
Rv2271	84	6.03	138	10.99	Non Essential
Rv2272	57	3.33	89	5.76	Non Essential
Rv2273	75	4.90	87	6.30	Non Essential
Rv2274c	4	0.27	7	0.53	Non Essential
Rv2274A	8	0.69	16	1.54	
Rv2275	96	2.37	161	4.41	Essential
Rv2276	118	2.13	191	3.82	Non Essential
Rv2277c	8	0.19	7	0.18	Essential
Rv2278	23	1.52	18	1.31	Non Essential
Rv2279	20	0.44	14	0.34	Non Essential
Rv2280	382	5.95	507	8.76	Non Essential
Rv2281	71	0.92	86	1.24	Non Essential
Rv2282c	34	0.78	43	1.09	Non Essential
Rv2283	0	0.00	0	0.00	Essential
Rv2284	31	0.51	57	1.05	Non Essential

Rv2285	49	0.79	68	1.21	Non Essential
Rv2286c	20	0.62	35	1.20	Non Essential
Rv2287	50	0.66	74	1.08	Non Essential
Rv2288	164	9.34	216	13.65	Non Essential
Rv2289	293	8.05	355	10.81	Non Essential
Rv2290	165	6.88	210	9.71	Non Essential
Rv2291	678	17.05	809	22.56	Non Essential
Rv2292c	44	4.22	55	5.85	Non Essential
Rv2293c	192	5.57	178	5.73	Non Essential
Rv2294	190	3.34	259	5.04	Non Essential
Rv2295	196	6.60	173	6.46	Non Essential
Rv2296	980	23.34	1002	26.46	Non Essential
Rv2297	896	42.58	766	40.36	Non Essential
Rv2298	136	3.01	168	4.12	Non Essential
Rv2299c	573	6.33	810	9.93	Non Essential
Rv2300c	16	0.37	12	0.31	Non Essential
Rv2301	146	4.53	147	5.06	Non Essential
Rv2302	279	24.76	315	31.00	Non Essential
Rv2303c	17	0.40	8	0.21	Non Essential
Rv2304c	9	0.92	7	0.80	Non Essential
Rv2305	40	0.67	31	0.57	Non Essential
Rv2306A	35	1.27	27	1.08	Non Essential
Rv2306B	17	0.84	17	0.93	Non Essential
Rv2307c	144	3.66	150	4.23	Non Essential
Rv2307A	12	1.35	11	1.37	Non Essential
Rv2307B	9	0.45	16	0.88	Non Essential
Rv2307D	23	2.71	18	2.36	Non Essential
Rv2308	52	1.56	52	1.73	Non Essential
Rv2309c	34	1.61	48	2.51	Non Essential
Rv2309A	19	1.42	3	0.25	Non Essential
Rv2310	2	0.12	6	0.42	Non Essential
Rv2311	18	0.74	14	0.64	Non Essential
Rv2312	12	0.96	22	1.95	Non Essential
Rv2313c	514	12.93	425	11.85	Non Essential
Rv2314c	241	3.77	196	3.40	Non Essential
Rv2315c	281	3.98	311	4.88	Non Essential
Rv2316	9	0.22	17	0.46	Non Essential
Rv2317	3	0.08	0	0.00	Non Essential
Rv2318	12	0.19	12	0.22	Non Essential
Rv2319c	104	2.54	70	1.90	Essential
Rv2320c	58	0.87	48	0.80	Non Essential
Rv2321c	17	0.67	11	0.48	Non Essential
Rv2322c	80	2.58	79	2.83	Non Essential
Rv2323c	17	0.40	13	0.34	Non Essential
Rv2324	19	0.92	19	1.01	Non Essential
Rv2325c	368	9.32	308	8.65	Essential
Rv2326c	1039	10.66	919	10.46	Non Essential
Rv2327	170	7.44	196	9.51	Non Essential
Rv2328	23	0.43	27	0.56	Non Essential
Rv2329c	263	3.65	347	5.34	Non Essential

Rv2330c	12	0.49	12	0.54	Non Essential
Rv2331	51	2.84	41	2.53	Non Essential
Rv2331A	37	2.35	23	1.62	Non Essential
Rv2332	120	1.57	96	1.39	Non Essential
Rv2333c	39	0.52	52	0.77	Non Essential
Rv2334	767	17.68	746	19.06	Non Essential
Rv2335	250	7.79	287	9.92	Essential
Rv2336	94	2.09	93	2.29	Non Essential
Rv2337c	152	2.92	155	3.30	Non Essential
Rv2338c	237	5.33	189	4.71	Essential
Rv2339	173	1.29	139	1.15	Non Essential
Rv2340c	292	5.05	249	4.78	Non Essential
Rv2341	8	0.41	3	0.17	Non Essential
Rv2342	173	14.46	167	15.48	Non Essential
Rv2343c	498	5.57	418	5.19	Essential
Rv2344c	289	4.79	277	5.09	Non Essential
Rv2345	210	2.28	246	2.96	Non Essential
Rv2346c	601	45.46	433	36.31	Non Essential
Rv2347c	936	67.92	729	58.65	Non Essential
Rv2348c	1712	112.81	1150	84.01	Non Essential
Rv2349c	175	2.46	144	2.25	Non Essential
Rv2350c	239	3.34	256	3.96	Non Essential
Rv2351c	60	0.84	67	1.04	Non Essential
Rv2352c	566	10.35	520	10.54	Non Essential
Rv2353c	5	0.10	8	0.18	Non Essential
Rv2354	15	0.99	15	1.10	Non Essential
Rv2355	12	0.26	17	0.41	Non Essential
Rv2356c	1133	13.18	1240	15.99	Non Essential
Rv2357c	204	3.15	263	4.50	Essential
Rv2358	39	2.06	54	3.16	Non Essential
Rv2359	76	4.16	46	2.79	Essential
Rv2360c	57	2.86	46	2.56	Non Essential
Rv2361c	97	2.34	97	2.60	Essential
Rv2362c	92	2.48	110	3.29	Non Essential
Rv2363	84	1.24	147	2.41	Non Essential
Rv2364c	353	8.41	188	4.96	Essential
Rv2365c	41	2.58	32	2.23	Essential
Rv2366c	918	15.09	613	11.17	Non Essential
Rv2367c	143	5.61	125	5.43	Non Essential
Rv2368c	844	17.14	509	11.46	Non Essential
Rv2369c	156	11.10	164	12.93	Non Essential
Rv2370c	27	0.44	25	0.45	Non Essential
Rv2371	6	0.70	9	1.16	Non Essential
Rv2372c	455	12.40	325	9.82	Non Essential
Rv2373c	1012	18.94	806	16.72	Essential
Rv2374c	826	17.21	776	17.93	Essential
Rv2375	54	3.66	58	4.36	Non Essential
Rv2376c	1938	82.27	1962	92.35	Non Essential
Rv2377c	350	34.97	67	7.42	Non Essential
Rv2378c	1222	20.27	175	3.22	Essential

Rv2379c	4819	23.61	707	3.84	Non Essential
Rv2380c	10113	43.03	1796	8.47	Non Essential
Rv2381c	4385	31.25	688	5.44	Non Essential
Rv2382c	1299	20.92	227	4.05	Essential
Rv2383c	2810	14.22	433	2.43	Non Essential
Rv2384	259	3.28	71	1.00	Non Essential
Rv2385	41	0.96	15	0.39	Non Essential
Rv2386c	1300	20.65	180	3.17	Essential
Rv2387	245	4.20	200	3.80	Essential
Rv2388c	173	3.30	137	2.90	Essential
Rv2389c	136	6.30	117	6.01	Non Essential
Rv2390c	68	2.62	74	3.16	Non Essential
Rv2391	2902	36.86	2835	39.93	Essential
Rv2392	383	10.77	372	11.60	Essential
Rv2393	470	11.95	481	13.56	Non Essential
Rv2394	371	4.13	356	4.39	Non Essential
Rv2395	371	3.98	320	3.80	Non Essential
Rv2396	1335	26.43	991	21.75	Non Essential
Rv2397c	95	1.93	100	2.26	Essential
Rv2398c	193	5.07	215	6.26	Essential
Rv2399c	51	1.29	52	1.46	Essential
Rv2400c	64	1.28	74	1.65	Essential
Rv2401	2	0.13	7	0.51	Non Essential
Rv2401A	7	0.74	10	1.17	Non Essential
Rv2402	177	1.97	207	2.56	Non Essential
Rv2403c	82	2.33	111	3.50	Non Essential
Rv2404c	175	1.92	223	2.71	Non Essential
Rv2405	188	7.10	174	7.28	Non Essential
Rv2406c	1297	65.09	992	55.20	Non Essential
Rv2407	12	0.31	5	0.15	Non Essential
Rv2408	6	0.18	9	0.30	Non Essential
Rv2409c	119	3.05	79	2.24	Non Essential
Rv2410c	130	2.86	104	2.54	Non Essential
Rv2411c	344	4.46	343	4.94	Non Essential
Rv2412	152	12.56	225	20.61	Essential
Rv2413c	144	3.26	142	3.56	Non Essential
Rv2414c	514	7.15	620	9.56	Non Essential
Rv2415c	185	4.45	275	7.33	Non Essential
Rv2416c	73	1.30	57	1.12	Non Essential
Rv2417c	386	9.85	351	9.93	Non Essential
Rv2418c	124	3.58	145	4.65	Essential
Rv2419c	239	7.65	277	9.83	Non Essential
Rv2420c	85	4.80	88	5.52	Non Essential
Rv2421c	94	3.18	108	4.05	Essential
Rv2422	1	0.08	0	0.00	Non Essential
Rv2423	20	0.41	24	0.55	Non Essential
Rv2424c	15	0.32	11	0.26	Non Essential
Rv2425c	254	3.78	238	3.93	Non Essential
Rv2426c	223	5.47	243	6.61	Non Essential
Rv2427c	462	7.96	482	9.21	Non Essential

Rv2427A	8	0.33	14	0.65	Non Essential
Rv2428	9	0.33	13	0.53	
Rv2429	11	0.44	8	0.36	Non Essential
Rv2430c	3274	120.42	2512	102.44	Non Essential
Rv2431c	1374	98.71	1099	87.54	Non Essential
Rv2432c	693	36.31	699	40.60	Non Essential
Rv2433c	16	1.19	23	1.89	Non Essential
Rv2434c	110	1.64	83	1.37	Non Essential
Rv2435c	128	1.25	103	1.12	Non Essential
Rv2436	84	1.97	112	2.92	Non Essential
Rv2437	30	1.54	54	3.07	Essential
Rv2438c	1023	10.78	1166	13.62	Essential
Rv2438A	0	0.00	0	0.00	Non Essential
Rv2439c	268	5.09	308	6.49	Essential
Rv2440c	336	5.02	371	6.14	Essential
Rv2441c	286	23.63	415	38.01	Essential
Rv2442c	753	51.51	1024	77.67	Essential
Rv2443	89	1.30	94	1.52	Non Essential
Rv2444c	7778	58.40	5391	44.88	Essential
Rv2445c	369	19.33	417	24.22	Non Essential
Rv2446c	91	5.27	100	6.42	Non Essential
Rv2447c	455	6.68	498	8.11	Essential
Rv2448c	1089	8.89	1212	10.98	Essential
Rv2449c	212	3.62	227	4.29	Non Essential
Rv2450c	14	0.58	10	0.46	Non Essential
Rv2451	2	0.11	5	0.30	Non Essential
Rv2452c	34	5.00	35	5.71	Non Essential
Rv2453c	216	7.67	176	6.93	Non Essential
Rv2454c	756	14.49	663	14.09	Non Essential
Rv2455c	1159	12.70	1109	13.47	Non Essential
Rv2456c	383	6.55	295	5.59	Non Essential
Rv2457c	4908	82.36	4147	77.16	Essential
Rv2458	29	0.69	45	1.18	Non Essential
Rv2459	158	2.22	107	1.67	Non Essential
Rv2460c	1751	58.40	1655	61.20	Essential
Rv2461c	636	22.69	677	26.78	Non Essential
Rv2462c	528	8.10	633	10.77	Non Essential
Rv2463	53	0.96	23	0.46	Non Essential
Rv2464c	167	4.45	186	5.50	Non Essential
Rv2465c	127	5.59	148	7.22	Non Essential
Rv2466c	5	0.17	18	0.69	Non Essential
Rv2467	386	3.21	463	4.27	Non Essential
Rv2468c	105	4.48	138	6.53	Non Essential
Rv2469c	67	2.15	75	2.67	Non Essential
Rv2470	152	8.46	148	9.13	Non Essential
Rv2471	270	3.54	227	3.30	Non Essential
Rv2472	11	0.81	9	0.73	Essential
Rv2473	21	0.63	26	0.86	Non Essential
Rv2474c	130	4.28	102	3.72	Non Essential
Rv2475c	319	16.47	289	16.55	Non Essential

Rv2476c	2362	10.41	2327	11.37	Non Essential
Rv2477c	1666	21.35	1822	25.89	Essential
Rv2478c	183	8.10	142	6.97	Non Essential
Rv2479c	13	0.28	12	0.29	Non Essential
Rv2480c	15	0.99	12	0.88	Non Essential
Rv2481c	9	0.60	7	0.52	Non Essential
Rv2482c	598	5.42	455	4.57	Non Essential
Rv2483c	1034	12.75	676	9.24	Essential
Rv2484c	1483	21.60	1114	17.99	Non Essential
Rv2485c	320	5.43	289	5.44	Non Essential
Rv2486	36	1.00	18	0.56	Non Essential
Rv2487c	110	1.13	111	1.27	Non Essential
Rv2488c	202	1.27	198	1.38	Non Essential
Rv2489c	23	1.65	18	1.43	Non Essential
Rv2490c	196	0.85	215	1.03	Non Essential
Rv2491	6	0.21	4	0.15	Non Essential
Rv2492	6	0.17	5	0.16	Non Essential
Rv2493	32	3.11	39	4.20	Non Essential
Rv2494	18	0.91	10	0.56	Non Essential
Rv2495c	2475	45.02	2212	44.61	Non Essential
Rv2496c	1035	21.25	971	22.11	Non Essential
Rv2497c	1313	25.57	1026	22.15	Non Essential
Rv2498c	386	10.10	326	9.46	Non Essential
Rv2499c	131	5.05	103	4.40	Non Essential
Rv2500c	716	12.99	585	11.77	Essential
Rv2501c	338	3.70	267	3.24	Non Essential
Rv2502c	971	13.13	811	12.16	Essential
Rv2503c	203	6.65	154	5.59	Non Essential
Rv2504c	206	5.93	142	4.53	Non Essential
Rv2505c	74	0.97	57	0.83	Non Essential
Rv2506	45	1.49	48	1.77	Non Essential
Rv2507	131	3.43	101	2.93	Essential
Rv2508c	36	0.58	68	1.21	Non Essential
Rv2509	113	3.01	95	2.81	Non Essential
Rv2510c	97	1.30	89	1.32	Essential
Rv2511	188	6.24	255	9.39	Essential
Rv2512c	3	0.05	3	0.06	Non Essential
Rv2513	365	18.58	387	21.84	Non Essential
Rv2514c	15	0.70	18	0.93	Non Essential
Rv2515c	23	0.40	14	0.27	Essential
Rv2516c	120	3.21	74	2.19	Essential
Rv2517c	57	4.88	32	3.04	Non Essential
Rv2518c	62	1.09	96	1.86	Non Essential
Rv2519	869	12.63	1044	16.82	Non Essential
Rv2520c	44	4.16	64	6.71	Non Essential
Rv2521	139	6.31	148	7.45	Non Essential
Rv2522c	241	3.67	231	3.90	Non Essential
Rv2523c	341	18.69	320	19.44	Non Essential
Rv2524c	5434	12.68	8285	21.43	Essential
Rv2525c	479	14.25	455	15.01	Non Essential

Rv2526	115	10.88	128	13.43	Non Essential
Rv2527	27	1.45	37	2.20	Non Essential
Rv2528c	2	0.05	13	0.34	Non Essential
Rv2529	10	0.15	21	0.36	Non Essential
Rv2530c	61	3.13	35	1.99	Non Essential
Rv2530A	123	11.80	99	10.53	Non Essential
Rv2531c	459	3.47	333	2.79	Non Essential
Rv2532c	55	2.95	50	2.97	Non Essential
Rv2533c	131	5.99	103	5.22	Essential
Rv2534c	374	14.27	329	13.92	Essential
Rv2535c	676	12.99	685	14.59	Non Essential
Rv2536	94	2.92	72	2.48	Non Essential
Rv2537c	55	2.67	45	2.42	Essential
Rv2538c	203	4.01	176	3.85	Essential
Rv2539c	126	5.11	121	5.44	Essential
Rv2540c	516	9.20	449	8.87	Essential
Rv2541	17	0.90	21	1.23	Non Essential
Rv2542	44	0.78	59	1.16	Non Essential
Rv2543	14	0.46	4	0.14	Non Essential
Rv2544	21	0.68	21	0.76	Non Essential
Rv2545	61	4.71	49	4.20	Non Essential
Rv2546	4	0.21	3	0.17	Non Essential
Rv2547	278	23.24	264	24.46	Non Essential
Rv2548	26	1.48	21	1.33	Non Essential
Rv2549c	31	1.69	43	2.59	Non Essential
Rv2550c	634	55.59	744	72.32	Non Essential
Rv2551c	71	3.64	59	3.35	Non Essential
Rv2552c	459	12.19	350	10.30	Essential
Rv2553c	587	10.06	424	8.06	Essential
Rv2554c	339	14.22	306	14.23	Essential
Rv2555c	2032	16.08	1800	15.80	Essential
Rv2556c	343	18.94	352	21.55	Non Essential
Rv2557	432	13.77	361	12.76	Non Essential
Rv2558	173	5.23	110	3.69	Non Essential
Rv2559c	54	0.85	52	0.91	Non Essential
Rv2560	43	0.95	77	1.88	Non Essential
Rv2561	42	3.08	54	4.39	Non Essential
Rv2562	21	1.16	37	2.27	Non Essential
Rv2563	108	2.21	146	3.31	Non Essential
Rv2564	79	1.71	121	2.90	Non Essential
Rv2565	992	12.17	906	12.32	Non Essential
Rv2566	139	0.87	163	1.13	Non Essential
Rv2567	784	6.35	794	7.12	Non Essential
Rv2568c	47	0.98	48	1.12	Non Essential
Rv2569c	62	1.41	64	1.61	Non Essential
Rv2570	3	0.17	3	0.18	Non Essential
Rv2571c	63	1.27	70	1.56	Non Essential
Rv2572c	329	3.95	429	5.71	Non Essential
Rv2573	9	0.26	7	0.23	Non Essential
Rv2574	65	2.78	68	3.22	Non Essential

Rv2575	56	1.37	66	1.78	Non Essential
Rv2576c	463	21.43	515	26.43	Non Essential
Rv2577	9	0.12	13	0.19	Non Essential
Rv2578c	24	0.50	20	0.47	Non Essential
Rv2579	33	0.79	35	0.92	Non Essential
Rv2580c	439	7.42	438	8.21	Essential
Rv2581c	307	9.78	437	15.44	Non Essential
Rv2582	203	4.71	363	9.34	Essential
Rv2583c	1510	13.67	1371	13.77	Non Essential
Rv2584c	711	22.76	561	19.91	Non Essential
Rv2585c	3616	46.43	3889	55.36	Non Essential
Rv2586c	890	14.40	860	15.42	Non Essential
Rv2587c	1425	17.79	1440	19.93	Non Essential
Rv2588c	389	24.08	419	28.76	Non Essential
Rv2589	272	4.33	264	4.66	Non Essential
Rv2590	3470	21.26	1851	12.57	Non Essential
Rv2591	923	12.16	673	9.83	Non Essential
Rv2592c	163	3.39	163	3.75	Non Essential
Rv2593c	164	5.97	150	6.05	Non Essential
Rv2594c	366	13.89	379	15.95	Non Essential
Rv2595	65	5.70	39	3.79	Non Essential
Rv2596	55	2.92	30	1.77	Non Essential
Rv2597	97	3.36	91	3.50	Non Essential
Rv2598	24	1.04	26	1.25	Non Essential
Rv2599	43	2.14	56	3.09	Non Essential
Rv2600	176	9.43	160	9.50	Non Essential
Rv2601	129	1.76	128	1.94	Non Essential
Rv2601A	98	7.33	103	8.55	Non Essential
Rv2602	37	1.81	37	2.00	Non Essential
Rv2603c	829	23.59	902	28.45	Non Essential
Rv2604c	257	9.26	245	9.79	Non Essential
Rv2605c	276	7.02	313	8.82	Non Essential
Rv2606c	446	10.66	506	13.40	Non Essential
Rv2607	33	1.05	46	1.63	Non Essential
Rv2608	410	5.06	398	5.44	Non Essential
Rv2609c	402	8.18	429	9.68	Non Essential
Rv2610c	1231	23.28	1150	24.11	Non Essential
Rv2611c	143	3.23	147	3.69	Essential
Rv2612c	206	6.78	212	7.73	Essential
Rv2613c	128	4.68	120	4.87	Essential
Rv2614c	261	2.70	296	3.39	Essential
Rv2614A	2	0.19	2	0.21	Non Essential
Rv2615c	1408	21.84	1544	26.55	Non Essential
Rv2616	12	0.52	13	0.62	Non Essential
Rv2617c	164	8.01	149	8.06	Non Essential
Rv2618	29	0.92	33	1.16	Non Essential
Rv2619c	128	7.79	92	6.21	Non Essential
Rv2620c	184	9.30	100	5.60	Non Essential
Rv2621c	172	5.48	102	3.60	Non Essential
Rv2622	54	1.41	36	1.04	Non Essential

Rv2623	38	0.91	73	1.95	Essential
Rv2624c	4	0.11	6	0.17	Non Essential
Rv2625c	9	0.16	22	0.44	Non Essential
Rv2626c	1	0.05	6	0.33	Non Essential
Rv2627c	23	0.40	48	0.92	Non Essential
Rv2628	2	0.12	11	0.72	Non Essential
Rv2629	175	3.34	139	2.95	Non Essential
Rv2630	29	1.16	32	1.41	Non Essential
Rv2631	151	2.50	98	1.80	Non Essential
Rv2632c	679	51.90	470	39.83	Non Essential
Rv2633c	310	13.73	229	11.24	Non Essential
Rv2634c	264	2.43	235	2.40	Non Essential
Rv2635	1	0.09	0	0.00	Non Essential
Rv2636	24	0.76	11	0.39	Non Essential
Rv2637	48	1.57	45	1.63	Non Essential
Rv2638	34	1.64	12	0.64	Non Essential
Rv2639c	20	1.29	8	0.57	Non Essential
Rv2640c	14	0.84	16	1.06	Non Essential
Rv2641	157	7.36	136	7.07	Non Essential
Rv2642	12	0.68	19	1.19	Non Essential
Rv2643	16	0.23	22	0.35	Non Essential
Rv2644c	3	0.20	6	0.45	Non Essential
Rv2645	9	0.45	17	0.94	Non Essential
Rv2646	20	0.43	26	0.62	Non Essential
Rv2647	5	0.29	7	0.45	Non Essential
Rv2648	11	0.72	21	1.53	Non Essential
Rv2649	9	0.20	9	0.22	Non Essential
Rv2650c	61	0.91	49	0.81	Non Essential
Rv2651c	64	2.58	57	2.55	Non Essential
Rv2652c	2	0.07	3	0.11	Non Essential
Rv2653c	0	0.00	0	0.00	Non Essential
Rv2654c	2	0.18	0	0.00	Non Essential
Rv2655c	20	0.30	16	0.27	Non Essential
Rv2656c	3	0.16	0	0.00	Non Essential
Rv2657c	43	3.55	26	2.38	Non Essential
Rv2658c	28	1.66	19	1.25	Non Essential
Rv2659c	18	0.34	17	0.36	Non Essential
Rv2660c	2	0.19	1	0.10	Non Essential
Rv2661c	3	0.17	3	0.18	Non Essential
Rv2662	0	0.00	0	0.00	Non Essential
Rv2663	24	2.21	25	2.56	Non Essential
Rv2664	24	2.03	21	1.97	Non Essential
Rv2665	97	7.41	133	11.27	Non Essential
Rv2666	6	0.16	3	0.09	Non Essential
Rv2667	22	0.62	14	0.44	Non Essential
Rv2668	44	1.81	41	1.87	Non Essential
Rv2669	18	0.82	15	0.76	Non Essential
Rv2670c	30	0.58	24	0.52	Non Essential
Rv2671	29	0.80	66	2.03	Non Essential
Rv2672	132	1.79	141	2.12	Non Essential

Rv2673	131	2.16	167	3.06	Essential
Rv2674	45	2.36	65	3.78	Non Essential
Rv2675c	113	3.23	114	3.61	Non Essential
Rv2676c	69	2.13	79	2.71	Non Essential
Rv2677c	404	6.39	360	6.31	Non Essential
Rv2678c	151	3.02	153	3.40	Non Essential
Rv2679	51	1.32	48	1.38	Non Essential
Rv2680	708	24.06	586	22.08	Non Essential
Rv2681	346	5.65	294	5.32	Non Essential
Rv2682c	1601	17.95	1173	14.58	Essential
Rv2683	63	2.72	92	4.41	Non Essential
Rv2684	368	6.13	329	6.08	Non Essential
Rv2685	316	5.28	280	5.19	Non Essential
Rv2686c	120	3.40	96	3.02	Non Essential
Rv2687c	77	2.32	53	1.77	Non Essential
Rv2688c	167	3.96	119	3.13	Non Essential
Rv2689c	202	3.57	180	3.52	Non Essential
Rv2690c	121	1.32	158	1.91	Non Essential
Rv2691	63	1.98	54	1.88	Non Essential
Rv2692	88	2.86	125	4.50	Essential
Rv2693c	88	2.82	72	2.56	Non Essential
Rv2694c	156	9.11	123	7.96	Non Essential
Rv2695	80	2.43	73	2.46	Non Essential
Rv2696c	1614	44.51	1648	50.38	Essential
Rv2697c	347	16.06	363	18.63	Essential
Rv2698	32	1.42	22	1.08	Essential
Rv2699c	522	37.13	670	52.84	Non Essential
Rv2700	19	0.63	38	1.39	Essential
Rv2701c	96	2.36	74	2.02	Non Essential
Rv2702	417	11.24	396	11.83	Essential
Rv2703	858	11.62	833	12.51	Essential
Rv2704	101	5.07	127	7.07	Non Essential
Rv2705c	3	0.17	6	0.37	Non Essential
Rv2706c	9	0.75	8	0.74	Non Essential
Rv2707	206	4.54	208	5.09	Essential
Rv2708c	797	69.03	926	88.93	Non Essential
Rv2709	86	4.14	79	4.22	Non Essential
Rv2710	5506	121.80	4620	113.32	Essential
Rv2711	1371	42.56	1107	38.10	Non Essential
Rv2712c	71	1.44	68	1.53	Non Essential
Rv2713	201	3.07	241	4.08	Non Essential
Rv2714	165	3.64	160	3.91	Non Essential
Rv2715	226	4.74	215	5.00	Non Essential
Rv2716	168	5.26	177	6.14	Non Essential
Rv2717c	30	1.30	31	1.49	Non Essential
Rv2718c	203	9.40	211	10.83	Non Essential
Rv2719c	101	4.37	117	5.61	Non Essential
Rv2720	280	8.47	236	7.92	Non Essential
Rv2721c	1676	17.15	1827	20.73	Non Essential
Rv2722	18	1.56	10	0.96	Non Essential

Rv2723	80	1.44	64	1.28	Non Essential
Rv2724c	907	16.80	577	11.85	Non Essential
Rv2725c	518	7.48	452	7.24	Non Essential
Rv2726c	654	16.17	643	17.62	Essential
Rv2727c	100	2.28	86	2.17	Essential
Rv2728c	26	0.80	20	0.69	Non Essential
Rv2729c	124	2.94	99	2.61	Non Essential
Rv2730	80	3.61	96	4.80	Non Essential
Rv2731	441	7.01	382	6.73	Non Essential
Rv2732c	190	6.65	205	7.95	Non Essential
Rv2733c	320	4.47	396	6.13	Non Essential
Rv2734	27	0.68	41	1.14	Essential
Rv2735c	142	3.07	142	3.41	Non Essential
Rv2736c	66	2.71	85	3.86	Non Essential
Rv2737c	1877	17.00	1749	17.56	Non Essential
Rv2737A	1	0.12	1	0.14	Non Essential
Rv2738c	78	8.13	69	7.98	Non Essential
Rv2739c	211	3.89	221	4.51	Essential
Rv2740	117	5.60	87	4.61	Non Essential
Rv2741	768	10.46	765	11.55	Non Essential
Rv2742c	43	1.11	30	0.86	Non Essential
Rv2743c	641	16.96	482	14.14	Non Essential
Rv2744c	842	22.27	625	18.33	Non Essential
Rv2745c	3867	245.75	3358	236.61	Non Essential
Rv2746c	101	3.45	80	3.03	Essential
Rv2747	119	4.88	213	9.68	Essential
Rv2748c	1142	9.25	1089	9.78	Essential
Rv2749	13	0.89	17	1.29	Non Essential
Rv2750	220	5.78	212	6.17	Non Essential
Rv2751	98	2.37	119	3.18	Non Essential
Rv2752c	581	7.45	483	6.86	Non Essential
Rv2753c	269	6.41	251	6.63	Essential
Rv2754c	17	0.49	29	0.92	Essential
Rv2755c	27	2.11	26	2.25	Non Essential
Rv2756c	355	4.70	327	4.80	Non Essential
Rv2757c	46	2.38	28	1.60	Non Essential
Rv2758c	96	7.75	68	6.09	Non Essential
Rv2759c	81	4.40	91	5.49	Non Essential
Rv2760c	36	2.87	34	3.01	Non Essential
Rv2761c	171	3.36	172	3.74	Non Essential
Rv2762c	142	7.28	130	7.39	Non Essential
Rv2763c	269	12.06	260	12.93	Non Essential
Rv2764c	409	11.11	367	11.05	Non Essential
Rv2765	14	0.41	15	0.48	Non Essential
Rv2766c	114	3.13	130	3.96	Non Essential
Rv2767c	16	0.97	11	0.74	Non Essential
Rv2768c	40	0.73	38	0.76	Non Essential
Rv2769c	15	0.39	20	0.58	Non Essential
Rv2770c	192	3.59	174	3.61	Non Essential
Rv2771c	640	30.41	512	26.98	Non Essential

Rv2772c	690	31.34	601	30.26	Non Essential
Rv2773c	394	11.48	340	10.99	Non Essential
Rv2774c	22	1.17	19	1.12	Non Essential
Rv2775	49	2.28	50	2.58	Non Essential
Rv2776c	215	4.97	186	4.77	Non Essential
Rv2777c	262	5.26	257	5.72	Non Essential
Rv2778c	342	15.63	293	14.85	Non Essential
Rv2779c	44	1.75	31	1.37	Non Essential
Rv2780	273	5.26	203	4.34	Non Essential
Rv2781c	274	5.69	274	6.31	Non Essential
Rv2782c	898	14.66	874	15.82	Non Essential
Rv2783c	2393	22.76	2721	28.70	Non Essential
Rv2784c	450	18.77	454	20.99	Non Essential
Rv2785c	219	17.49	215	19.03	Non Essential
Rv2786c	150	3.24	130	3.11	Essential
Rv2787	22	0.27	30	0.41	Non Essential
Rv2788	194	6.07	169	5.87	Non Essential
Rv2789c	683	11.91	641	12.39	Non Essential
Rv2790c	1037	18.49	941	18.60	Non Essential
Rv2791c	6658	103.71	5653	97.63	Non Essential
Rv2792c	1173	43.37	836	34.27	Non Essential
Rv2793c	848	20.33	801	21.29	Non Essential
Rv2794c	158	4.97	198	6.90	Non Essential
Rv2795c	662	14.60	1499	36.65	Non Essential
Rv2796c	108	4.12	99	4.19	Non Essential
Rv2797c	78	0.99	98	1.38	Non Essential
Rv2798c	316	20.82	325	23.74	Non Essential
Rv2799	234	7.99	259	9.81	Non Essential
Rv2800	119	1.55	129	1.86	Non Essential
Rv2801c	9	0.54	15	1.00	Non Essential
Rv2801A	53	4.95	58	6.01	
Rv2802c	209	4.30	209	4.77	Non Essential
Rv2803	24	1.10	20	1.02	Non Essential
Rv2804c	1	0.03	0	0.00	Non Essential
Rv2805	73	3.88	82	4.83	Non Essential
Rv2806	1	0.11	1	0.12	Non Essential
Rv2807	3	0.06	5	0.10	Non Essential
Rv2808	528	44.13	640	59.31	Essential
Rv2809	593	40.96	644	49.32	Non Essential
Rv2810c	555	29.73	524	31.12	Non Essential
Rv2811	19	0.67	20	0.78	Non Essential
Rv2812	4	0.06	2	0.03	Essential
Rv2813	1	0.03	2	0.06	Essential
Rv2814c	9	0.20	8	0.19	Non Essential
Rv2815c	16	1.05	12	0.88	Non Essential
Rv2816c	33	2.08	38	2.65	Non Essential
Rv2817c	40	0.85	55	1.29	Essential
Rv2818c	113	2.11	191	3.96	Non Essential
Rv2819c	112	2.13	165	3.49	Non Essential
Rv2820c	76	1.80	135	3.54	Non Essential

Rv2821c	32	0.97	104	3.49	Non Essential
Rv2822c	42	2.41	91	5.79	Non Essential
Rv2823c	63	0.56	128	1.26	Non Essential
Rv2824c	100	2.28	191	4.82	Non Essential
Rv2825c	433	14.38	461	16.97	Non Essential
Rv2826c	132	3.21	98	2.64	Non Essential
Rv2827c	198	4.79	156	4.19	Essential
Rv2828c	156	6.15	125	5.46	Essential
Rv2828A	45	3.59	48	4.25	
Rv2829c	17	0.93	34	2.07	Non Essential
Rv2830c	125	12.49	117	12.96	Essential
Rv2831	58	1.66	64	2.03	Non Essential
Rv2832c	44	0.87	48	1.06	Essential
Rv2833c	30	0.49	32	0.58	Essential
Rv2834c	23	0.60	18	0.52	Non Essential
Rv2835c	34	0.80	32	0.84	Non Essential
Rv2836c	690	11.24	702	12.68	Non Essential
Rv2837c	1115	23.71	1014	23.91	Essential
Rv2838c	2203	85.88	2255	97.47	Non Essential
Rv2839c	4397	34.96	4459	39.30	Essential
Rv2840c	3080	221.27	2432	193.71	Non Essential
Rv2841c	314	6.47	242	5.53	Essential
Rv2842c	582	22.69	481	20.79	Non Essential
Rv2843	14	0.55	30	1.31	Non Essential
Rv2844	483	21.26	749	36.55	Non Essential
Rv2845c	250	3.07	214	2.92	Essential
Rv2846c	2237	30.18	1473	22.04	Essential
Rv2847c	207	3.65	212	4.15	Non Essential
Rv2848c	228	3.57	185	3.21	Non Essential
Rv2849c	721	24.86	636	24.31	Non Essential
Rv2850c	790	8.98	671	8.46	Non Essential
Rv2851c	354	16.18	300	15.20	Non Essential
Rv2852c	776	11.26	647	10.40	Non Essential
Rv2853	156	1.81	137	1.77	Non Essential
Rv2854	46	0.95	43	0.98	Non Essential
Rv2855	183	2.85	180	3.11	Essential
Rv2856	685	13.16	848	18.06	Essential
Rv2857c	172	4.76	140	4.30	Essential
Rv2858c	179	2.81	194	3.38	Non Essential
Rv2859c	112	2.60	100	2.57	Non Essential
Rv2860c	590	9.23	642	11.14	Non Essential
Rv2861c	462	11.58	557	15.48	Non Essential
Rv2862c	65	2.39	51	2.08	Non Essential
Rv2862A	0	0.00	0	0.00	
Rv2863	1	0.06	2	0.13	Non Essential
Rv2864c	329	3.90	452	5.94	Non Essential
Rv2865	62	4.74	63	5.34	Non Essential
Rv2866	4	0.33	4	0.36	Non Essential
Rv2867c	491	12.35	417	11.63	Non Essential
Rv2868c	1913	35.33	1645	33.69	Non Essential

Rv2869c	416	7.36	382	7.49	Essential
Rv2870c	219	3.79	219	4.20	Non Essential
Rv2871	11	0.92	15	1.39	Non Essential
Rv2872	17	0.82	29	1.56	Non Essential
Rv2873	28	0.91	18	0.65	Non Essential
Rv2874	12	0.12	15	0.17	Non Essential
Rv2875	8	0.30	11	0.45	Non Essential
Rv2876	33	2.26	47	3.56	Non Essential
Rv2877c	90	2.24	50	1.38	Non Essential
Rv2878c	103	4.25	70	3.20	Non Essential
Rv2879c	430	16.23	379	15.86	Non Essential
Rv2880c	287	7.45	226	6.51	Non Essential
Rv2881c	814	19.01	641	16.59	Essential
Rv2882c	564	21.75	483	20.65	Essential
Rv2883c	830	22.71	738	22.39	Essential
Rv2884	642	18.19	504	15.84	Non Essential
Rv2885c	201	3.12	212	3.65	Essential
Rv2886c	194	4.70	176	4.73	Non Essential
Rv2887	200	10.25	285	16.20	Non Essential
Rv2888c	1003	15.16	979	16.41	Non Essential
Rv2889c	1077	28.39	1148	33.55	Essential
Rv2890c	565	14.06	773	21.33	Essential
Rv2891	2	0.06	2	0.06	Non Essential
Rv2892c	69	1.21	78	1.52	Non Essential
Rv2893	11	0.24	10	0.24	Non Essential
Rv2894c	207	4.96	208	5.53	Non Essential
Rv2895c	307	7.75	290	8.12	Non Essential
Rv2896c	127	2.33	134	2.73	Non Essential
Rv2897c	48	0.68	38	0.60	Essential
Rv2898c	101	5.62	81	5.00	Non Essential
Rv2899c	107	2.77	114	3.27	Non Essential
Rv2900c	2005	18.41	1716	17.47	Non Essential
Rv2901c	529	37.26	462	36.08	Non Essential
Rv2902c	1118	30.25	1062	31.85	Non Essential
Rv2903c	742	18.03	887	23.90	Essential
Rv2904c	226	14.24	270	18.86	Essential
Rv2905	688	15.66	593	14.96	Non Essential
Rv2906c	51	1.58	51	1.76	Essential
Rv2907c	148	6.00	162	7.28	Essential
Rv2908c	89	7.90	117	11.51	Essential
Rv2909c	166	7.31	221	10.79	Non Essential
Rv2910c	19	0.92	16	0.86	Non Essential
Rv2911	49	1.20	62	1.69	Non Essential
Rv2912c	28	1.02	34	1.38	Essential
Rv2913c	129	1.51	153	1.99	Non Essential
Rv2914c	225	2.75	194	2.63	Non Essential
Rv2915c	563	10.88	583	12.49	Non Essential
Rv2916c	531	7.23	561	8.47	Essential
Rv2917	16	0.18	35	0.44	Non Essential
Rv2918c	399	3.53	342	3.36	Non Essential

Rv2919c	739	46.96	562	39.60	Non Essential
Rv2920c	382	5.73	293	4.87	Non Essential
Rv2921c	828	14.03	787	14.78	Essential
Rv2922c	1421	8.44	1561	10.28	Non Essential
Rv2922A	18	1.38	26	2.20	Non Essential
Rv2923c	66	3.43	36	2.08	Non Essential
Rv2924c	105	2.60	104	2.85	Non Essential
Rv2925c	337	10.03	267	8.81	Essential
Rv2926c	158	5.45	201	7.68	Essential
Rv2927c	705	20.55	821	26.53	Essential
Rv2928	194	5.31	372	11.29	Non Essential
Rv2929	80	5.53	100	7.66	Non Essential
Rv2930	344	4.22	320	4.35	Non Essential
Rv2931	902	3.44	708	2.99	Non Essential
Rv2932	3105	14.45	1705	8.80	Non Essential
Rv2933	3044	9.96	1734	6.29	Non Essential
Rv2934	3755	14.71	2820	12.25	Non Essential
Rv2935	2359	11.35	1926	10.27	Non Essential
Rv2936	1066	23.01	792	18.96	Essential
Rv2937	106	2.62	109	2.99	Essential
Rv2938	105	2.72	79	2.27	Essential
Rv2939	429	7.27	304	5.71	Non Essential
Rv2940c	12190	41.33	9989	37.55	Non Essential
Rv2941	3610	44.51	3604	49.27	Non Essential
Rv2942	1610	12.52	1143	9.86	Essential
Rv2943	153	2.65	175	3.36	Non Essential
Rv2943A	48	1.95	47	2.11	Non Essential
Rv2944	51	1.53	50	1.66	Non Essential
Rv2945c	171	5.24	150	5.10	Essential
Rv2946c	2415	10.70	1817	8.92	Non Essential
Rv2947c	1524	21.97	1159	18.53	Non Essential
Rv2948c	2170	22.02	1583	17.81	Non Essential
Rv2949c	849	30.45	575	22.86	Non Essential
Rv2950c	2651	30.63	2115	27.10	Non Essential
Rv2951c	799	14.99	795	16.54	Non Essential
Rv2952	224	5.93	291	8.53	Non Essential
Rv2953	293	5.01	293	5.56	Non Essential
Rv2954c	262	7.76	243	7.98	Non Essential
Rv2955c	97	2.16	56	1.38	Non Essential
Rv2956	177	5.20	220	7.17	Non Essential
Rv2957	49	1.27	68	1.96	Non Essential
Rv2958c	66	1.10	64	1.19	Non Essential
Rv2959c	122	3.56	153	4.94	Non Essential
Rv2960c	10	0.87	19	1.82	Non Essential
Rv2961	1	0.06	3	0.18	Non Essential
Rv2962c	61	0.97	108	1.91	Non Essential
Rv2963	164	2.89	205	4.00	Non Essential
Rv2964	12	0.28	12	0.31	Non Essential
Rv2965c	629	27.86	429	21.07	Non Essential
Rv2966c	105	3.98	69	2.90	Non Essential

Rv2967c	932	5.92	762	5.36	Essential
Rv2968c	59	2.01	70	2.64	Essential
Rv2969c	264	7.39	318	9.87	Essential
Rv2970c	236	4.49	247	5.21	Non Essential
Rv2970A	557	70.38	625	87.56	Non Essential
Rv2971	442	11.20	407	11.43	Essential
Rv2972c	62	1.87	83	2.77	Non Essential
Rv2973c	167	1.62	180	1.94	Non Essential
Rv2974c	159	2.42	145	2.45	Non Essential
Rv2975c	235	19.87	249	23.35	Non Essential
Rv2976c	267	8.40	233	8.12	Essential
Rv2977c	1069	22.94	979	23.29	Essential
Rv2978c	289	4.50	251	4.33	Non Essential
Rv2979c	243	8.94	255	10.40	Non Essential
Rv2980	39	1.54	69	3.02	Non Essential
Rv2981c	259	4.96	259	5.50	Essential
Rv2982c	165	3.53	165	3.91	Non Essential
Rv2983	32	1.07	30	1.11	Non Essential
Rv2984	332	3.20	329	3.52	Non Essential
Rv2985	34	0.77	45	1.12	Non Essential
Rv2986c	5836	194.66	5695	210.61	Essential
Rv2987c	1153	41.56	536	21.42	Essential
Rv2988c	3515	53.13	1721	28.84	Non Essential
Rv2989	647	19.83	385	13.08	Non Essential
Rv2990c	6143	153.44	3358	92.99	Non Essential
Rv2991	335	14.66	279	13.53	Non Essential
Rv2992c	560	8.17	601	9.72	Essential
Rv2993c	100	2.99	99	3.28	Non Essential
Rv2994	153	2.46	159	2.83	Non Essential
Rv2995c	190	4.04	207	4.88	Essential
Rv2996c	922	12.49	1163	17.46	Essential
Rv2997	41	0.61	53	0.88	Non Essential
Rv2998	15	0.70	16	0.83	Essential
Rv2998A	0	0.00	5	0.59	Non Essential
Rv2999	41	0.91	45	1.11	Essential
Rv3000	13	0.42	19	0.69	Non Essential
Rv3001c	1654	35.49	1880	44.73	Essential
Rv3002c	287	12.18	362	17.04	Essential
Rv3003c	414	4.79	442	5.67	Essential
Rv3004	17	1.08	21	1.48	Non Essential
Rv3005c	681	17.44	797	22.62	Non Essential
Rv3006	322	6.17	312	6.63	Non Essential
Rv3007c	14	0.49	17	0.66	Non Essential
Rv3008	63	2.17	106	4.05	Non Essential
Rv3009c	781	10.97	672	10.47	Essential
Rv3010c	729	15.19	947	21.88	Non Essential
Rv3011c	113	1.64	179	2.87	Essential
Rv3012c	7	0.50	9	0.72	Non Essential
Rv3013	9	0.29	10	0.36	Non Essential
Rv3014c	132	1.37	189	2.17	Essential

Rv3015c	63	1.34	88	2.07	Non Essential
Rv3016	24	0.82	27	1.02	Non Essential
Rv3017c	24	1.42	44	2.89	Non Essential
Rv3018c	12	0.20	12	0.22	Essential
Rv3018A	4	1.00	5	1.38	Non Essential
Rv3019c	9	0.67	4	0.33	Non Essential
Rv3020c	12	0.88	19	1.54	Non Essential
Rv3021c	11	0.22	14	0.31	Non Essential
Rv3022c	0	0.00	1	0.10	Non Essential
Rv3022A	2	0.14	1	0.08	Non Essential
Rv3023c	3	0.05	3	0.06	Non Essential
Rv3024c	360	7.01	307	6.63	Non Essential
Rv3025c	632	11.50	532	10.73	Essential
Rv3026c	32	0.75	38	0.99	Non Essential
Rv3027c	61	1.55	52	1.47	Non Essential
Rv3028c	1074	24.13	1226	30.54	Non Essential
Rv3029c	1253	33.64	1293	38.49	Essential
Rv3030	99	2.58	83	2.40	Essential
Rv3031	195	2.65	174	2.62	Essential
Rv3032	162	2.80	126	2.41	Essential
Rv3033	10	0.39	11	0.48	Non Essential
Rv3034c	242	5.76	241	6.36	Non Essential
Rv3035	317	6.29	330	7.26	Non Essential
Rv3036c	77	2.42	80	2.79	Non Essential
Rv3037c	74	1.48	60	1.33	Non Essential
Rv3038c	418	9.13	330	8.00	Non Essential
Rv3039c	75	2.11	65	2.03	Non Essential
Rv3040c	47	1.17	58	1.60	Non Essential
Rv3041c	134	3.34	156	4.31	Non Essential
Rv3042c	628	10.98	782	15.15	Essential
Rv3043c	289	3.61	437	6.05	Essential
Rv3044	132	2.63	180	3.97	Non Essential
Rv3045	1553	32.08	1569	35.93	Non Essential
Rv3046c	25	1.44	17	1.08	Non Essential
Rv3047c	116	8.77	70	5.87	Non Essential
Rv3048c	341	7.52	256	6.26	Essential
Rv3049c	671	9.16	529	8.00	Non Essential
Rv3050c	462	13.41	401	12.91	Essential
Rv3051c	11689	120.66	9047	103.54	Essential
Rv3052c	1368	65.01	1152	60.70	Non Essential
Rv3053c	332	29.84	239	23.82	Non Essential
Rv3054c	16	0.62	17	0.73	Non Essential
Rv3055	23	0.80	25	0.97	Non Essential
Rv3056	59	1.22	60	1.37	Non Essential
Rv3057c	168	4.18	132	3.64	Non Essential
Rv3058c	204	6.74	200	7.33	Non Essential
Rv3059	154	2.24	167	2.69	Non Essential
Rv3060c	382	5.57	313	5.06	Non Essential
Rv3061c	548	5.44	371	4.08	Essential
Rv3062	67	0.94	71	1.11	Non Essential

Rv3063	65	0.61	88	0.92	Non Essential
Rv3064c	26	1.31	17	0.95	Non Essential
Rv3065	43	2.86	16	1.18	Non Essential
Rv3066	18	0.64	9	0.35	Non Essential
Rv3067	9	0.47	10	0.58	Non Essential
Rv3068c	242	3.16	258	3.74	Non Essential
Rv3069	22	1.19	24	1.44	Non Essential
Rv3070	16	0.90	17	1.07	Non Essential
Rv3071	20	0.39	23	0.49	Non Essential
Rv3072c	61	2.50	63	2.86	Non Essential
Rv3073c	11	0.66	17	1.14	Non Essential
Rv3074	169	2.85	207	3.87	Non Essential
Rv3075c	866	20.15	1128	29.11	Non Essential
Rv3076	35	1.58	49	2.45	Non Essential
Rv3077	61	0.72	98	1.29	Non Essential
Rv3078	15	0.80	27	1.60	Non Essential
Rv3079c	48	1.25	70	2.02	Non Essential
Rv3080c	131	0.84	130	0.93	Non Essential
Rv3081	71	1.23	40	0.77	Non Essential
Rv3082c	14	0.29	10	0.23	Non Essential
Rv3083	321	4.64	189	3.03	Non Essential
Rv3084	51	1.18	31	0.80	Non Essential
Rv3085	81	2.10	46	1.32	Non Essential
Rv3086	663	12.88	463	9.97	Non Essential
Rv3087	217	3.29	156	2.62	Essential
Rv3088	215	3.24	142	2.37	Non Essential
Rv3089	448	6.37	413	6.51	Non Essential
Rv3090	13	0.31	17	0.46	Non Essential
Rv3091	161	2.05	148	2.08	Non Essential
Rv3092c	218	5.09	277	7.17	Non Essential
Rv3093c	241	5.16	558	13.24	Non Essential
Rv3094c	467	8.88	845	17.81	Non Essential
Rv3095	35	1.58	69	3.45	Non Essential
Rv3096	139	2.62	157	3.28	Non Essential
Rv3097c	15	0.25	8	0.15	Non Essential
Rv3098c	7	0.33	11	0.58	Non Essential
Rv3099c	670	16.91	705	19.73	Non Essential
Rv3100c	50	2.23	45	2.22	Non Essential
Rv3101c	290	6.98	368	9.81	Essential
Rv3102c	91	2.84	109	3.77	Non Essential
Rv3103c	67	3.29	68	3.71	Essential
Rv3104c	368	8.54	329	8.46	Non Essential
Rv3105c	506	9.57	492	10.31	Essential
Rv3106	104	1.63	153	2.66	Non Essential
Rv3107c	50	0.68	50	0.75	Non Essential
Rv3108	0	0.00	1	0.05	Non Essential
Rv3109	20	0.40	11	0.24	Essential
Rv3110	10	0.54	8	0.48	Non Essential
Rv3111	3	0.13	0	0.00	Essential
Rv3112	3	0.26	2	0.19	Essential

Rv3113	4	0.13	5	0.18	Essential
Rv3114	1	0.04	2	0.09	Essential
Rv3115	4	0.07	4	0.08	Non Essential
Rv3116	696	12.79	666	13.57	Non Essential
Rv3117	1654	42.65	2057	58.81	Non Essential
Rv3118	816	58.04	927	73.10	Non Essential
Rv3119	158	7.66	183	9.84	Non Essential
Rv3120	131	4.67	168	6.65	Non Essential
Rv3121	140	2.50	118	2.34	Non Essential
Rv3122	155	7.08	169	8.56	Non Essential
Rv3123	23	1.00	27	1.30	Non Essential
Rv3124	12	0.30	11	0.30	Non Essential
Rv3125c	16	0.29	14	0.28	Non Essential
Rv3126c	0	0.00	0	0.00	Non Essential
Rv3127	142	2.95	228	5.25	Non Essential
Rv3128c	0	0.00	3	0.07	
Rv3129	0	0.00	0	0.00	Non Essential
Rv3130c	225	3.47	537	9.19	Non Essential
Rv3131	46	0.99	108	2.58	Essential
Rv3132c	387	4.79	315	4.32	Essential
Rv3133c	152	5.00	200	7.29	Non Essential
Rv3134c	137	3.65	244	7.21	Non Essential
Rv3135	112	6.04	182	10.89	Essential
Rv3136	1232	23.17	1829	38.14	Non Essential
Rv3137	215	5.91	226	6.88	Essential
Rv3138	37	0.73	29	0.63	Non Essential
Rv3139	536	8.19	538	9.11	Essential
Rv3140	763	13.60	795	15.71	Essential
Rv3141	377	8.34	387	9.49	Non Essential
Rv3142c	224	11.24	217	12.07	Non Essential
Rv3143	2	0.11	2	0.12	Non Essential
Rv3144c	502	8.77	469	9.09	Non Essential
Rv3145	294	16.36	496	30.60	Non Essential
Rv3146	964	37.38	1365	58.68	Non Essential
Rv3147	480	14.52	735	24.65	Non Essential
Rv3148	464	7.54	697	12.56	Non Essential
Rv3149	370	10.49	519	16.31	Non Essential
Rv3150	382	6.14	518	9.23	Non Essential
Rv3151	1554	13.79	1777	17.49	Non Essential
Rv3152	1377	24.01	1575	30.45	Non Essential
Rv3153	1090	36.87	1199	44.97	Non Essential
Rv3154	514	14.01	604	18.25	Non Essential
Rv3155	60	4.31	45	3.58	Non Essential
Rv3156	2173	24.55	2218	27.79	Non Essential
Rv3157	1214	15.70	1164	16.69	Non Essential
Rv3158	488	6.57	419	6.26	Non Essential
Rv3159c	864	10.47	524	7.04	Non Essential
Rv3160c	211	7.07	134	4.98	Non Essential
Rv3161c	1301	24.34	804	16.68	Non Essential
Rv3162c	21	1.03	14	0.76	Non Essential

Rv3163c	56	0.95	41	0.77	Non Essential
Rv3164c	96	2.14	82	2.03	Non Essential
Rv3165c	40	1.78	40	1.98	Non Essential
Rv3166c	23	0.52	29	0.72	Non Essential
Rv3167c	2	0.07	9	0.34	Non Essential
Rv3168	64	1.21	57	1.19	Essential
Rv3169	92	1.76	87	1.84	Non Essential
Rv3170	94	1.50	112	1.98	Non Essential
Rv3171c	482	11.52	509	13.48	Non Essential
Rv3172c	41	1.83	31	1.53	Non Essential
Rv3173c	738	26.33	654	25.87	Non Essential
Rv3174	25	0.76	11	0.37	Non Essential
Rv3175	6	0.09	5	0.08	Non Essential
Rv3176c	24	0.54	13	0.32	Non Essential
Rv3177	6	0.15	2	0.06	Essential
Rv3178	0	0.00	0	0.00	Essential
Rv3179	38	0.63	38	0.70	Non Essential
Rv3180c	5	0.25	3	0.16	Non Essential
Rv3181c	14	0.67	13	0.68	Non Essential
Rv3182	14	0.87	11	0.76	Non Essential
Rv3183	3	0.20	3	0.22	Non Essential
Rv3184	9	0.59	17	1.24	Non Essential
Rv3185	6	0.13	10	0.24	Non Essential
Rv3186	10	0.66	9	0.66	Non Essential
Rv3187	10	0.22	16	0.39	Non Essential
Rv3188	94	5.82	76	5.22	Non Essential
Rv3189	20	0.69	12	0.46	Non Essential
Rv3190c	110	1.87	123	2.32	Non Essential
Rv3191c	21	0.44	18	0.41	Non Essential
Rv3192	0	0.00	0	0.00	Non Essential
Rv3193c	893	6.44	918	7.34	Non Essential
Rv3194c	180	3.78	264	6.15	Non Essential
Rv3195	51	0.77	44	0.74	Non Essential
Rv3196	8	0.19	9	0.24	Non Essential
Rv3196A	33	3.54	31	3.69	Non Essential
Rv3197	862	13.79	753	13.35	Non Essential
Rv3197A	3	0.23	7	0.60	Non Essential
Rv3198c	579	5.92	387	4.38	Essential
Rv3198A	59	4.99	82	7.69	Non Essential
Rv3199c	289	6.60	236	5.97	Non Essential
Rv3200c	444	8.94	454	10.13	Non Essential
Rv3201c	124	0.81	125	0.90	Essential
Rv3202c	174	1.18	141	1.06	Non Essential
Rv3203	50	1.59	43	1.52	Non Essential
Rv3204	42	2.96	32	2.50	Non Essential
Rv3205c	412	10.08	481	13.05	Non Essential
Rv3206c	705	12.86	670	13.55	Non Essential
Rv3207c	280	7.02	281	7.81	Non Essential
Rv3208	317	9.93	323	11.21	Non Essential
Rv3208A	147	11.61	227	19.88	Non Essential

Rv3209	116	4.45	111	4.72	Non Essential
Rv3210c	53	1.64	38	1.30	Essential
Rv3211	1970	26.73	1205	18.13	Non Essential
Rv3212	2101	36.90	1306	25.43	Non Essential
Rv3213c	1841	49.43	2468	73.47	Non Essential
Rv3214	164	5.77	217	8.46	Non Essential
Rv3215	67	1.29	55	1.17	Non Essential
Rv3216	158	7.03	174	8.58	Non Essential
Rv3217c	95	4.73	102	5.64	Non Essential
Rv3218	135	3.01	189	4.66	Non Essential
Rv3219	6595	557.73	6408	600.83	Non Essential
Rv3220c	2235	31.90	2184	34.56	Non Essential
Rv3221c	504	50.35	462	51.18	Non Essential
Rv3221A	137	9.65	158	12.34	Non Essential
Rv3222c	365	14.23	393	16.99	Non Essential
Rv3223c	644	21.28	579	21.21	Non Essential
Rv3224	1426	36.12	1497	42.04	Non Essential
Rv3224A	75	8.57	84	10.64	Non Essential
Rv3224B	47	4.63	46	5.03	Non Essential
Rv3225c	272	4.10	228	3.81	Non Essential
Rv3226c	151	4.28	154	4.84	Non Essential
Rv3227	149	2.37	158	2.78	Non Essential
Rv3228	37	0.80	41	0.98	Non Essential
Rv3229c	9461	158.40	4978	92.40	Essential
Rv3230c	1183	22.25	634	13.22	Non Essential
Rv3231c	71	3.00	57	2.67	Non Essential
Rv3232c	418	10.12	370	9.93	Non Essential
Rv3233c	177	6.44	169	6.82	Non Essential
Rv3234c	189	4.98	161	4.70	Non Essential
Rv3235	9	0.30	10	0.37	Non Essential
Rv3236c	462	8.58	450	9.26	Essential
Rv3237c	339	15.11	259	12.80	Non Essential
Rv3238c	50	1.46	47	1.52	Non Essential
Rv3239c	227	1.55	192	1.45	Non Essential
Rv3240c	1102	8.31	965	8.07	Essential
Rv3241c	363	12.11	277	10.24	Non Essential
Rv3242c	27	0.90	14	0.52	Non Essential
Rv3243c	71	1.81	73	2.06	Essential
Rv3244c	586	7.19	451	6.13	Essential
Rv3245c	1624	20.48	1316	18.40	Essential
Rv3246c	1553	48.63	1515	52.60	Essential
Rv3247c	177	5.90	185	6.84	Non Essential
Rv3248c	5103	73.72	5112	81.87	Essential
Rv3249c	128	4.33	147	5.51	Non Essential
Rv3250c	402	47.45	545	71.32	Non Essential
Rv3251c	59	7.59	77	10.98	Non Essential
Rv3252c	143	2.46	133	2.53	Non Essential
Rv3253c	65	0.94	59	0.94	Non Essential
Rv3254	153	2.37	135	2.32	Non Essential
Rv3255c	137	2.40	142	2.76	Essential

Rv3256c	384	7.93	335	7.67	Non Essential
Rv3257c	1464	22.51	1361	23.20	Essential
Rv3258c	598	26.16	828	40.16	Essential
Rv3259	16	0.82	13	0.74	Non Essential
Rv3260c	507	40.49	494	43.74	Non Essential
Rv3261	232	5.01	289	6.92	Non Essential
Rv3262	105	1.68	121	2.14	Non Essential
Rv3263	54	0.70	45	0.65	Non Essential
Rv3264c	1250	24.88	1414	31.21	Essential
Rv3265c	1282	30.43	1305	34.34	Essential
Rv3266c	178	4.18	189	4.92	Non Essential
Rv3267	1365	19.60	1290	20.54	Essential
Rv3268	182	5.67	175	6.05	Non Essential
Rv3269	317	24.23	253	21.44	Non Essential
Rv3270	1064	10.60	813	8.98	Essential
Rv3271c	388	12.48	475	16.93	Essential
Rv3272	154	2.79	225	4.53	Non Essential
Rv3273	958	8.97	920	9.55	Non Essential
Rv3274c	452	8.31	550	11.21	Non Essential
Rv3275c	266	10.90	327	14.86	Essential
Rv3276c	406	6.77	508	9.39	Non Essential
Rv3277	92	2.42	115	3.35	Essential
Rv3278c	17	0.70	14	0.64	Non Essential
Rv3279c	35	0.94	31	0.92	Non Essential
Rv3280	514	6.71	632	9.14	Non Essential
Rv3281	2172	87.53	2101	93.88	Essential
Rv3282	206	6.62	210	7.49	Non Essential
Rv3283	1241	29.85	1252	33.39	Non Essential
Rv3284	288	14.35	215	11.88	Non Essential
Rv3285	1845	21.99	2070	27.36	Essential
Rv3286c	162	4.43	100	3.03	Non Essential
Rv3287c	150	7.37	120	6.54	Non Essential
Rv3288c	520	27.05	378	21.80	Non Essential
Rv3289c	104	5.93	46	2.91	Non Essential
Rv3290c	659	10.49	357	6.30	Non Essential
Rv3291c	73	3.47	90	4.74	Non Essential
Rv3292	80	1.38	89	1.70	Non Essential
Rv3293	200	2.89	223	3.58	Non Essential
Rv3294c	7	0.19	6	0.18	Non Essential
Rv3295	1886	60.92	1648	59.02	Non Essential
Rv3296	456	2.16	337	1.77	Non Essential
Rv3297	69	1.93	49	1.52	Non Essential
Rv3298c	90	2.12	89	2.32	Non Essential
Rv3299c	980	7.23	970	7.93	Non Essential
Rv3300c	83	1.94	94	2.44	Non Essential
Rv3301c	744	24.03	723	25.89	Non Essential
Rv3302c	284	3.47	285	3.86	Non Essential
Rv3303c	291	4.22	354	5.69	Non Essential
Rv3304	68	3.05	74	3.68	Non Essential
Rv3305c	43	0.79	50	1.02	Non Essential

Rv3306c	794	14.40	708	14.24	Non Essential
Rv3307	91	2.43	116	3.43	Non Essential
Rv3308	354	4.74	370	5.49	Non Essential
Rv3309c	6	0.21	9	0.34	Non Essential
Rv3310	99	2.37	109	2.89	Non Essential
Rv3311	473	8.05	462	8.72	Non Essential
Rv3312c	161	3.73	97	2.49	Non Essential
Rv3312A	66	4.56	63	4.82	Non Essential
Rv3313c	84	1.64	70	1.52	Non Essential
Rv3314c	124	2.08	125	2.32	Non Essential
Rv3315c	81	4.34	54	3.21	Non Essential
Rv3316	16	1.02	14	0.99	Non Essential
Rv3317	38	1.88	29	1.59	Non Essential
Rv3318	251	3.04	251	3.37	Non Essential
Rv3319	109	2.96	98	2.95	Non Essential
Rv3320c	87	4.37	113	6.29	Non Essential
Rv3321c	151	13.40	176	17.32	Non Essential
Rv3322c	40	1.40	28	1.09	Non Essential
Rv3323c	47	1.52	42	1.50	Non Essential
Rv3324c	15	0.60	18	0.80	Non Essential
Rv3324A	4	0.64	1	0.18	
Rv3325	19	1.25	17	1.24	Non Essential
Rv3326	12	0.26	6	0.14	Non Essential
Rv3327	15	0.19	14	0.19	Non Essential
Rv3328c	108	2.47	104	2.64	Non Essential
Rv3329	83	1.35	105	1.90	Non Essential
Rv3330	144	2.54	205	4.01	Non Essential
Rv3331	230	3.28	217	3.43	Non Essential
Rv3332	101	1.88	98	2.03	Essential
Rv3333c	8	0.20	4	0.11	Non Essential
Rv3334	24	1.17	22	1.19	Non Essential
Rv3335c	127	3.14	139	3.81	Essential
Rv3336c	278	5.91	292	6.89	Essential
Rv3337	41	2.28	35	2.16	Non Essential
Rv3338	86	2.87	78	2.88	Non Essential
Rv3339c	1812	31.67	1769	34.28	Non Essential
Rv3340	127	2.02	153	2.70	Non Essential
Rv3341	170	3.21	231	4.83	Essential
Rv3342	64	1.88	81	2.64	Non Essential
Rv3343c	1003	2.85	940	2.96	Essential
Rv3344c	31	0.46	35	0.57	Non Essential
Rv3345c	158	0.74	127	0.66	Non Essential
Rv3346c	15	1.25	12	1.11	Non Essential
Rv3347c	679	1.54	629	1.58	Non Essential
Rv3348	28	1.22	36	1.75	Non Essential
Rv3349c	5	0.15	3	0.10	Non Essential
Rv3350c	292	0.56	309	0.66	Non Essential
Rv3351c	3	0.08	4	0.12	Non Essential
Rv3352c	2	0.12	1	0.06	Non Essential
Rv3353c	14	1.16	17	1.56	Non Essential

Rv3354	21	1.16	16	0.98	Non Essential
Rv3355c	17	1.25	24	1.95	Non Essential
Rv3356c	319	8.11	383	10.79	Essential
Rv3357	112	8.75	124	10.74	Non Essential
Rv3358	11	0.92	14	1.30	Non Essential
Rv3359	12	0.22	15	0.30	Non Essential
Rv3360	16	0.93	9	0.58	Non Essential
Rv3361c	46	1.79	43	1.86	Non Essential
Rv3362c	134	4.95	141	5.78	Non Essential
Rv3363c	38	2.22	35	2.27	Non Essential
Rv3364c	196	10.74	167	10.15	Non Essential
Rv3365c	633	5.17	487	4.41	Non Essential
Rv3366	10	0.46	26	1.33	Non Essential
Rv3367	330	4.01	327	4.41	Non Essential
Rv3368c	35	1.17	28	1.04	Non Essential
Rv3369	98	4.85	112	6.15	Non Essential
Rv3370c	60	0.40	61	0.45	Non Essential
Rv3371	104	1.67	89	1.58	Essential
Rv3372	97	1.77	110	2.23	Essential
Rv3373	262	8.78	337	12.52	Non Essential
Rv3374	38	3.29	63	6.05	Non Essential
Rv3375	99	1.49	107	1.79	Essential
Rv3376	347	11.41	319	11.63	Non Essential
Rv3377c	327	4.67	308	4.87	Non Essential
Rv3378c	109	2.63	106	2.84	Non Essential
Rv3379c	14	0.19	7	0.10	Non Essential
Rv3380c	12	0.26	7	0.17	Non Essential
Rv3381c	15	0.99	18	1.31	Non Essential
Rv3382c	240	5.21	236	5.68	Non Essential
Rv3383c	311	6.35	317	7.18	Non Essential
Rv3384c	30	1.64	39	2.37	Non Essential
Rv3385c	79	5.51	101	7.81	Non Essential
Rv3386	47	1.43	61	2.06	Non Essential
Rv3387	8	0.25	12	0.42	Non Essential
Rv3388	134	1.31	140	1.52	Non Essential
Rv3389c	378	9.31	398	10.87	Non Essential
Rv3390	126	3.81	80	2.68	Non Essential
Rv3391	116	1.28	65	0.79	Non Essential
Rv3392c	20	0.50	15	0.41	Non Essential
Rv3393	54	1.25	73	1.88	Non Essential
Rv3394c	25	0.34	40	0.60	Non Essential
Rv3395c	164	5.74	176	6.83	Non Essential
Rv3395A	5	0.17	3	0.11	Non Essential
Rv3396c	264	3.60	318	4.80	Essential
Rv3397c	93	2.20	100	2.62	Non Essential
Rv3398c	51	1.02	65	1.43	Essential
Rv3399	97	1.99	119	2.71	Non Essential
Rv3400	238	6.49	250	7.56	Essential
Rv3401	606	5.52	687	6.93	Non Essential
Rv3402c	1328	23.04	177	3.41	Non Essential

Rv3403c	1163	15.60	257	3.82	Non Essential
Rv3404c	135	4.12	134	4.53	Non Essential
Rv3405c	43	1.63	35	1.47	Non Essential
Rv3406	15	0.36	8	0.21	Non Essential
Rv3407	5054	363.08	4894	389.81	Non Essential
Rv3408	962	50.40	954	55.42	Non Essential
Rv3409c	303	3.75	271	3.72	Non Essential
Rv3410c	523	9.97	349	7.38	Non Essential
Rv3411c	1549	20.94	1208	18.11	Essential
Rv3412	484	25.36	361	20.97	Non Essential
Rv3413c	618	14.77	591	15.66	Non Essential
Rv3414c	1242	41.82	1261	47.07	Non Essential
Rv3415c	31	0.81	30	0.86	Non Essential
Rv3416	15	1.05	12	0.93	Non Essential
Rv3417c	5093	67.57	5941	87.39	Essential
Rv3418c	6143	436.94	6334	499.50	Essential
Rv3419c	256	5.32	220	5.07	Essential
Rv3420c	232	10.47	221	11.06	Non Essential
Rv3421c	77	2.60	66	2.48	Non Essential
Rv3422c	127	5.39	87	4.09	Essential
Rv3423c	287	5.03	262	5.09	Essential
Rv3424c	4	0.24	1	0.07	Non Essential
Rv3425	29	1.18	14	0.63	Non Essential
Rv3426	41	1.26	59	2.01	Non Essential
Rv3427c	26	0.74	34	1.07	Essential
Rv3428c	1	0.02	2	0.04	Non Essential
Rv3429	1064	42.64	1105	49.10	Non Essential
Rv3430c	71	1.31	45	0.92	Non Essential
Rv3431c	78	1.98	43	1.21	Non Essential
Rv3432c	196	3.05	141	2.43	Non Essential
Rv3433c	122	1.84	114	1.91	Non Essential
Rv3434c	90	2.71	82	2.74	Non Essential
Rv3435c	437	10.99	466	13.00	Non Essential
Rv3436c	525	6.02	431	5.48	Essential
Rv3437	41	1.85	37	1.85	Non Essential
Rv3438	48	1.22	71	2.01	Non Essential
Rv3439c	167	2.56	162	2.75	Non Essential
Rv3440c	70	4.83	91	6.97	Non Essential
Rv3441c	760	12.13	831	14.70	Essential
Rv3442c	149	7.03	267	13.98	Non Essential
Rv3443c	281	13.63	447	24.03	Essential
Rv3444c	10	0.71	14	1.10	Non Essential
Rv3445c	7	0.47	5	0.38	Non Essential
Rv3446c	8	0.14	7	0.14	Non Essential
Rv3447c	17	0.10	10	0.06	Non Essential
Rv3448	3	0.05	8	0.14	Non Essential
Rv3449	10	0.16	9	0.16	Non Essential
Rv3450c	80	1.22	91	1.53	Non Essential
Rv3451	53	1.44	52	1.57	Non Essential
Rv3452	61	1.93	52	1.82	Non Essential

Rv3453	1	0.06	0	0.00	Non Essential
Rv3454	8	0.14	9	0.17	Non Essential
Rv3455c	732	20.42	772	23.88	Essential
Rv3456c	1641	65.04	1831	80.46	Non Essential
Rv3457c	2686	55.32	2799	63.91	Essential
Rv3458c	1384	49.14	1683	66.25	Essential
Rv3459c	859	44.04	1014	57.64	Essential
Rv3460c	1706	97.98	1945	123.85	Non Essential
Rv3461c	885	168.23	1438	303.07	Non Essential
Rv3462c	2100	204.11	2123	228.78	Essential
Rv3463	24	0.60	49	1.36	Non Essential
Rv3464	111	2.40	111	2.66	Essential
Rv3465	123	4.35	129	5.05	Essential
Rv3466	129	4.15	112	3.99	Non Essential
Rv3467	16	0.36	16	0.40	Non Essential
Rv3468c	32	0.63	39	0.85	Non Essential
Rv3469c	52	1.11	52	1.23	Non Essential
Rv3470c	103	1.33	98	1.41	Non Essential
Rv3471c	2	0.08	4	0.18	Non Essential
Rv3472	300	12.74	289	13.60	Essential
Rv3473c	4	0.11	1	0.03	Non Essential
Rv3474	15	0.99	16	1.17	Non Essential
Rv3475	7	0.15	5	0.12	Non Essential
Rv3476c	138	2.20	200	3.53	Non Essential
Rv3477	2109	153.05	1928	155.12	Non Essential
Rv3478	6913	125.74	5903	119.04	Non Essential
Rv3479	873	6.12	543	4.22	Non Essential
Rv3480c	368	5.29	469	7.48	Non Essential
Rv3481c	124	3.87	159	5.50	Non Essential
Rv3482c	124	3.41	144	4.39	Non Essential
Rv3483c	158	5.13	171	6.15	Non Essential
Rv3484	162	2.26	137	2.12	Essential
Rv3485c	548	12.47	317	8.00	Non Essential
Rv3486	6	0.29	6	0.32	Non Essential
Rv3487c	830	21.40	542	15.50	Non Essential
Rv3488	20	1.33	10	0.74	Non Essential
Rv3489	465	60.90	370	53.73	Essential
Rv3490	1083	15.49	782	12.40	Essential
Rv3491	2010	74.70	1704	70.21	Non Essential
Rv3492c	198	8.82	154	7.61	Non Essential
Rv3493c	508	14.99	474	15.51	Non Essential
Rv3494c	400	5.07	438	6.16	Non Essential
Rv3495c	288	5.36	231	4.77	Essential
Rv3496c	322	5.10	296	5.20	Non Essential
Rv3497c	119	2.38	87	1.93	Essential
Rv3498c	146	2.98	164	3.71	Non Essential
Rv3499c	184	3.29	187	3.71	Essential
Rv3500c	109	2.78	150	4.24	Non Essential
Rv3501c	79	2.22	101	3.15	Essential
Rv3502c	98	2.21	97	2.42	Essential

Rv3503c	168	18.89	147	18.33	Non Essential
Rv3504	33	0.59	29	0.57	Non Essential
Rv3505	147	2.82	159	3.38	Non Essential
Rv3506	44	0.63	48	0.76	Non Essential
Rv3507	406	2.10	317	1.82	Non Essential
Rv3508	1794	6.75	1300	5.43	Non Essential
Rv3509c	196	2.72	201	3.09	Non Essential
Rv3510c	102	2.62	112	3.19	Non Essential
Rv3511	110	1.10	91	1.01	Non Essential
Rv3512	118	0.78	107	0.79	Non Essential
Rv3513c	47	1.54	36	1.31	Non Essential
Rv3514	326	1.57	271	1.44	Non Essential
Rv3515c	143	1.87	79	1.14	Non Essential
Rv3516	21	0.57	35	1.05	Non Essential
Rv3517	24	0.61	31	0.88	Non Essential
Rv3518c	409	7.35	363	7.23	Non Essential
Rv3519	312	9.44	392	13.15	Essential
Rv3520c	611	12.58	505	11.53	Non Essential
Rv3521	32	0.75	28	0.73	Non Essential
Rv3522	124	2.50	89	1.99	Non Essential
Rv3523	140	2.54	234	4.71	Essential
Rv3524	162	3.38	294	6.79	Non Essential
Rv3525c	27	1.11	29	1.32	Non Essential
Rv3526	393	7.28	265	5.44	Non Essential
Rv3527	22	1.05	8	0.42	Non Essential
Rv3528c	65	1.96	80	2.67	Non Essential
Rv3529c	19	0.35	20	0.41	Non Essential
Rv3530c	22	0.60	13	0.40	Non Essential
Rv3531c	24	0.46	17	0.36	Non Essential
Rv3532	28	0.49	24	0.47	Non Essential
Rv3533c	249	3.06	195	2.66	Non Essential
Rv3534c	208	4.30	172	3.94	Essential
Rv3535c	181	4.27	170	4.44	Non Essential
Rv3536c	104	2.85	79	2.40	Non Essential
Rv3537	138	1.75	139	1.96	Non Essential
Rv3538	54	1.35	40	1.11	Essential
Rv3539	21	0.31	28	0.46	Non Essential
Rv3540c	268	4.96	249	5.11	Essential
Rv3541c	59	3.26	55	3.37	Essential
Rv3542c	140	3.22	102	2.60	Essential
Rv3543c	91	1.68	69	1.41	Non Essential
Rv3544c	145	3.06	137	3.20	Essential
Rv3545c	104	1.72	94	1.72	Essential
Rv3546	38	0.69	34	0.69	Non Essential
Rv3547	24	1.13	26	1.36	Non Essential
Rv3548c	130	3.06	146	3.80	Non Essential
Rv3549c	50	1.38	57	1.74	Non Essential
Rv3550	34	0.98	34	1.09	Non Essential
Rv3551	32	0.78	23	0.62	Essential
Rv3552	18	0.51	27	0.86	Non Essential

Rv3553	77	1.55	53	1.18	Non Essential
Rv3554	64	0.67	84	0.97	Non Essential
Rv3555c	121	2.99	92	2.52	Non Essential
Rv3556c	789	14.61	745	15.30	Essential
Rv3557c	126	4.50	130	5.14	Non Essential
Rv3558	43	0.56	60	0.86	Non Essential
Rv3559c	146	3.98	130	3.93	Essential
Rv3560c	66	1.23	78	1.61	Essential
Rv3561	57	0.80	41	0.64	Non Essential
Rv3562	136	2.58	157	3.30	Non Essential
Rv3563	63	1.41	51	1.27	Essential
Rv3564	47	1.06	41	1.02	Essential
Rv3565	22	0.41	16	0.33	Non Essential
Rv3566c	168	4.24	161	4.51	Non Essential
Rv3566A	15	1.21	17	1.52	Non Essential
Rv3567c	132	5.04	110	4.65	Non Essential
Rv3568c	321	7.64	193	5.10	Non Essential
Rv3569c	326	8.00	253	6.89	Non Essential
Rv3570c	482	8.74	324	6.52	Non Essential
Rv3571	182	3.63	135	2.99	Non Essential
Rv3572	144	5.84	112	5.03	Non Essential
Rv3573c	87	0.88	62	0.69	Non Essential
Rv3574	70	2.51	63	2.50	Essential
Rv3575c	176	3.50	257	5.67	Non Essential
Rv3576	192	5.78	216	7.21	Non Essential
Rv3577	89	2.21	97	2.67	Non Essential
Rv3578	99	1.71	94	1.80	Non Essential
Rv3579c	1208	26.81	1372	33.76	Essential
Rv3580c	695	10.60	684	11.56	Essential
Rv3581c	509	22.83	331	16.46	Essential
Rv3582c	1284	39.68	759	26.01	Essential
Rv3583c	3790	166.83	2515	122.74	Non Essential
Rv3584	59	2.31	104	4.52	Non Essential
Rv3585	154	2.29	155	2.56	Non Essential
Rv3586	74	1.48	83	1.84	Non Essential
Rv3587c	363	9.82	364	10.92	Non Essential
Rv3588c	324	11.17	253	9.67	Essential
Rv3589	77	1.81	107	2.79	Non Essential
Rv3590c	207	2.54	196	2.66	Non Essential
Rv3591c	18	0.50	25	0.77	Non Essential
Rv3592	605	41.00	615	46.20	Non Essential
Rv3593	556	8.79	542	9.51	Essential
Rv3594	71	1.84	71	2.04	Non Essential
Rv3595c	257	4.19	198	3.58	Non Essential
Rv3596c	6857	57.85	5391	50.43	Essential
Rv3597c	564	35.84	594	41.85	Essential
Rv3598c	257	3.64	399	6.26	Essential
Rv3599c	67	17.34	56	16.07	Non Essential
Rv3600c	767	20.14	619	18.02	Non Essential
Rv3601c	232	11.89	249	14.15	Non Essential

Rv3602c	211	4.88	192	4.92	Essential
Rv3603c	536	12.64	372	9.73	Non Essential
Rv3604c	245	4.41	262	5.23	Essential
Rv3605c	175	7.90	115	5.75	Non Essential
Rv3606c	125	4.74	110	4.63	Non Essential
Rv3607c	399	21.37	262	15.56	Essential
Rv3608c	599	15.28	414	11.71	Essential
Rv3609c	343	12.12	242	9.48	Essential
Rv3610c	1361	12.81	1174	12.25	Essential
Rv3611	2	0.07	3	0.11	Essential
Rv3612c	109	7.12	46	3.33	Non Essential
Rv3613c	32	4.27	19	2.81	Non Essential
Rv3614c	7641	296.27	4324	185.88	Essential
Rv3615c	333	23.00	256	19.60	Essential
Rv3616c	7093	129.34	4329	87.52	Essential
Rv3617	79	1.75	61	1.50	Non Essential
Rv3618	144	2.61	75	1.50	Non Essential
Rv3619c	107	8.09	120	10.06	Non Essential
Rv3620c	283	20.54	275	22.13	Non Essential
Rv3621c	23	0.40	15	0.29	Non Essential
Rv3622c	0	0.00	2	0.16	Non Essential
Rv3623	56	1.67	47	1.55	Non Essential
Rv3624c	143	4.73	200	7.33	Non Essential
Rv3625c	108	2.39	106	2.60	Essential
Rv3626c	240	4.90	228	5.16	Non Essential
Rv3627c	1686	26.15	1909	32.83	Essential
Rv3628	48	2.11	100	4.88	Non Essential
Rv3629c	58	1.14	63	1.37	Non Essential
Rv3630	167	2.77	180	3.31	Non Essential
Rv3631	160	4.74	146	4.80	Essential
Rv3632	54	3.37	45	3.12	Non Essential
Rv3633	309	7.59	252	6.86	Non Essential
Rv3634c	62	1.41	68	1.72	Essential
Rv3635	73	0.88	63	0.85	Essential
Rv3636	93	5.76	93	6.38	Non Essential
Rv3637	7	0.30	1	0.05	Non Essential
Rv3638	0	0.00	2	0.06	Non Essential
Rv3639c	2	0.08	0	0.00	Non Essential
Rv3640c	2	0.03	5	0.10	Non Essential
Rv3641c	298	10.08	242	9.08	Non Essential
Rv3642c	178	19.71	204	25.04	Non Essential
Rv3643	0	0.00	0	0.00	Non Essential
Rv3644c	534	9.52	528	10.44	Non Essential
Rv3645	216	2.81	247	3.57	Non Essential
Rv3646c	829	6.35	1049	8.91	Essential
Rv3647c	278	10.33	270	11.13	Non Essential
Rv3648c	2860	302.63	2268	266.08	Essential
Rv3649	85	0.79	87	0.90	Essential
Rv3650	4	0.30	12	1.01	Non Essential
Rv3651	160	3.31	176	4.04	Essential

Rv3652	41	2.80	33	2.50	Non Essential
Rv3653	31	1.13	32	1.30	Non Essential
Rv3654c	37	3.13	28	2.63	Non Essential
Rv3655c	15	0.85	7	0.44	Non Essential
Rv3656c	62	6.46	59	6.82	Non Essential
Rv3657c	63	2.35	62	2.57	Non Essential
Rv3658c	48	1.29	50	1.49	Essential
Rv3659c	79	1.60	84	1.89	Non Essential
Rv3660c	109	2.23	111	2.51	Essential
Rv3661	747	18.59	775	21.39	Non Essential
Rv3662c	6	0.17	16	0.49	Non Essential
Rv3663c	70	0.91	87	1.26	Essential
Rv3664c	14	0.38	14	0.42	Non Essential
Rv3665c	19	0.44	32	0.82	Non Essential
Rv3666c	28	0.37	41	0.60	Essential
Rv3667	82	0.90	108	1.32	Non Essential
Rv3668c	28	0.86	35	1.19	Non Essential
Rv3669	301	12.48	336	15.45	Essential
Rv3670	179	3.91	207	5.02	Non Essential
Rv3671c	377	6.79	384	7.67	Non Essential
Rv3672c	491	12.85	462	13.40	Non Essential
Rv3673c	492	15.47	504	17.57	Non Essential
Rv3674c	338	9.85	409	13.22	Non Essential
Rv3675	116	6.61	115	7.26	Non Essential
Rv3676	1088	34.67	1144	40.42	Non Essential
Rv3677c	424	11.47	410	12.30	Non Essential
Rv3678c	329	15.53	383	20.05	Non Essential
Rv3678A	35	4.67	35	5.18	Non Essential
Rv3679	852	17.91	842	19.62	Essential
Rv3680	921	17.05	837	17.18	Non Essential
Rv3681c	108	6.52	68	4.55	Non Essential
Rv3682	1207	10.66	1174	11.50	Non Essential
Rv3683	106	2.37	106	2.63	Essential
Rv3684	222	4.59	237	5.43	Non Essential
Rv3685c	246	3.70	166	2.76	Non Essential
Rv3686c	207	13.39	166	11.91	Non Essential
Rv3687c	125	7.30	80	5.18	Non Essential
Rv3688c	762	35.28	759	38.96	Non Essential
Rv3689	438	6.94	412	7.24	Non Essential
Rv3690	52	1.71	47	1.71	Non Essential
Rv3691	71	1.52	68	1.62	Non Essential
Rv3692	206	4.11	222	4.91	Non Essential
Rv3693	340	5.52	312	5.62	Non Essential
Rv3694c	333	7.21	356	8.55	Non Essential
Rv3695	194	4.47	174	4.45	Non Essential
Rv3696c	320	4.43	286	4.39	Non Essential
Rv3697c	45	2.21	41	2.23	Non Essential
Rv3697A	36	3.45	45	4.78	
Rv3698	49	0.69	56	0.87	Non Essential
Rv3699	284	8.70	341	11.59	Non Essential

Rv3700c	298	5.46	216	4.39	Non Essential
Rv3701c	987	21.97	727	17.94	Essential
Rv3702c	186	5.70	150	5.10	Non Essential
Rv3703c	201	3.38	173	3.23	Non Essential
Rv3704c	384	6.35	272	4.99	Non Essential
Rv3705c	120	4.00	55	2.03	Non Essential
Rv3705A	24	1.33	30	1.84	Non Essential
Rv3706c	46	3.09	43	3.20	Non Essential
Rv3707c	112	2.38	118	2.78	Non Essential
Rv3708c	434	8.99	455	10.45	Essential
Rv3709c	1482	25.17	1508	28.39	Essential
Rv3710	677	7.52	611	7.52	Essential
Rv3711c	223	4.84	273	6.57	Non Essential
Rv3712	154	2.67	198	3.80	Essential
Rv3713	63	1.95	101	3.46	Essential
Rv3714c	64	1.54	68	1.82	Non Essential
Rv3715c	785	27.60	761	29.66	Non Essential
Rv3716c	562	30.10	631	37.48	Non Essential
Rv3717	167	4.95	189	6.21	Essential
Rv3718c	26	1.26	35	1.88	Non Essential
Rv3719	847	12.89	777	13.11	Non Essential
Rv3720	341	5.80	297	5.60	Non Essential
Rv3721c	348	4.31	378	5.19	Essential
Rv3722c	585	9.61	533	9.71	Essential
Rv3723	893	25.11	954	29.74	Essential
Rv3724A	120	10.65	127	12.50	Non Essential
Rv3724B	93	3.55	84	3.55	Non Essential
Rv3725	309	7.14	372	9.54	Non Essential
Rv3726	371	6.68	388	7.75	Non Essential
Rv3727	116	1.38	96	1.26	Non Essential
Rv3728	54	0.36	69	0.51	Non Essential
Rv3729	91	0.84	88	0.90	Non Essential
Rv3730c	83	1.71	95	2.18	Non Essential
Rv3731	129	2.58	116	2.57	Non Essential
Rv3732	110	2.23	150	3.38	Non Essential
Rv3733c	212	9.11	119	5.67	Non Essential
Rv3734c	547	8.61	376	6.57	Non Essential
Rv3735	82	3.61	90	4.39	Non Essential
Rv3736	80	1.62	66	1.48	Non Essential
Rv3737	163	2.20	146	2.19	Non Essential
Rv3738c	31	0.70	35	0.88	Non Essential
Rv3739c	2	0.18	12	1.23	Non Essential
Rv3740c	35	0.56	44	0.78	Non Essential
Rv3741c	4	0.13	1	0.04	Non Essential
Rv3742c	1	0.05	1	0.06	Non Essential
Rv3743c	3	0.03	2	0.02	Non Essential
Rv3744	15	0.89	14	0.92	Non Essential
Rv3745c	0	0.00	0	0.00	Non Essential
Rv3746c	12	0.77	14	1.00	Non Essential
Rv3747	187	10.49	219	13.62	Non Essential

Rv3748	120	7.18	113	7.50	Non Essential
Rv3749c	410	17.30	338	15.81	Non Essential
Rv3750c	243	13.32	196	11.91	Non Essential
Rv3751	0	0.00	0	0.00	Non Essential
Rv3752c	143	6.71	113	5.88	Essential
Rv3753c	269	11.56	183	8.72	Non Essential
Rv3754	26	0.62	39	1.03	Non Essential
Rv3755c	312	11.19	253	10.06	Non Essential
Rv3756c	139	4.15	132	4.37	Non Essential
Rv3757c	152	4.74	131	4.53	Non Essential
Rv3758c	273	5.19	221	4.66	Essential
Rv3759c	288	6.53	334	8.40	Non Essential
Rv3760	12	0.85	15	1.18	Non Essential
Rv3761c	84	1.71	114	2.57	Non Essential
Rv3762c	93	1.06	90	1.14	Non Essential
Rv3763	1667	74.76	1677	83.38	Non Essential
Rv3764c	57	0.86	82	1.37	Non Essential
Rv3765c	438	13.36	478	16.17	Non Essential
Rv3766	66	2.06	64	2.21	Non Essential
Rv3767c	732	16.66	518	13.07	Non Essential
Rv3768	23	1.38	22	1.46	Non Essential
Rv3769	56	4.42	65	5.69	Non Essential
Rv3770c	43	1.61	45	1.86	Non Essential
Rv3770A	13	1.53	22	2.88	Non Essential
Rv3770B	58	6.52	110	13.72	Non Essential
Rv3771c	54	3.56	29	2.12	Non Essential
Rv3772	54	1.09	66	1.48	Non Essential
Rv3773c	109	4.01	91	3.71	Non Essential
Rv3774	1196	31.18	1497	43.27	Non Essential
Rv3775	168	2.89	238	4.55	Non Essential
Rv3776	50	0.69	49	0.75	Non Essential
Rv3777	196	4.27	203	4.90	Non Essential
Rv3778c	1744	31.32	1634	32.54	Essential
Rv3779	707	7.59	652	7.76	Non Essential
Rv3780	890	35.67	930	41.32	Non Essential
Rv3781	527	13.79	450	13.05	Essential
Rv3782	459	10.79	378	9.85	Essential
Rv3783	294	7.50	270	7.64	Non Essential
Rv3784	82	1.80	66	1.60	Non Essential
Rv3785	356	7.13	277	6.15	Non Essential
Rv3786c	242	4.25	299	5.82	Non Essential
Rv3787c	79	1.83	102	2.62	Non Essential
Rv3788	99	4.38	169	8.30	Non Essential
Rv3789	11	0.65	18	1.17	Non Essential
Rv3790	51	0.79	89	1.53	Essential
Rv3791	221	6.21	228	7.11	Essential
Rv3792	135	1.50	154	1.90	Essential
Rv3793	373	2.44	335	2.43	Essential
Rv3794	590	3.86	825	5.98	Essential
Rv3795	840	5.47	969	7.00	Essential

Rv3796	47	0.90	53	1.12	Non Essential
Rv3797	84	1.01	57	0.76	Non Essential
Rv3798	195	3.14	185	3.30	Non Essential
Rv3799c	4032	55.24	2966	45.05	Essential
Rv3800c	12644	52.22	10065	46.09	Essential
Rv3801c	1424	15.99	1323	16.47	Essential
Rv3802c	644	13.70	515	12.14	Essential
Rv3803c	943	22.53	996	26.39	Non Essential
Rv3804c	2499	52.83	2817	66.03	Non Essential
Rv3805c	1014	11.57	916	11.59	Essential
Rv3806c	1176	27.82	1037	27.20	Essential
Rv3807c	237	10.24	226	10.83	Non Essential
Rv3808c	565	6.34	672	8.37	Essential
Rv3809c	86	1.54	125	2.48	Non Essential
Rv3810	452	11.37	546	15.23	Essential
Rv3811	347	4.60	352	5.18	Non Essential
Rv3812	39	0.55	45	0.71	Non Essential
Rv3813c	126	3.30	106	3.07	Non Essential
Rv3814c	569	15.57	492	14.93	Non Essential
Rv3815c	255	7.25	250	7.89	Non Essential
Rv3816c	774	21.34	721	22.04	Non Essential
Rv3817	67	1.91	62	1.96	Non Essential
Rv3818	169	2.34	185	2.84	Non Essential
Rv3819	12	0.77	14	1.00	Non Essential
Rv3820c	107	1.63	204	3.46	Non Essential
Rv3821	75	2.26	55	1.84	Non Essential
Rv3822	481	8.51	285	5.59	Non Essential
Rv3823c	2592	17.03	1176	8.57	Non Essential
Rv3824c	776	10.86	409	6.35	Non Essential
Rv3825c	6905	23.25	4017	14.99	Non Essential
Rv3826	247	3.02	182	2.47	Non Essential
Rv3827c	283	4.96	245	4.76	Non Essential
Rv3828c	169	5.94	156	6.08	Non Essential
Rv3829c	64	0.85	56	0.83	Non Essential
Rv3830c	8	0.27	4	0.15	Non Essential
Rv3831	7	0.31	13	0.64	Non Essential
Rv3832c	15	0.56	16	0.66	Non Essential
Rv3833	10	0.27	16	0.48	Non Essential
Rv3834c	57	0.97	63	1.19	Essential
Rv3835	83	1.32	91	1.61	Non Essential
Rv3836	25	1.30	22	1.27	Non Essential
Rv3837c	210	6.46	159	5.43	Non Essential
Rv3838c	167	3.72	142	3.50	Non Essential
Rv3839	234	6.48	15	0.46	Non Essential
Rv3840	32	1.66	2	0.12	Non Essential
Rv3841	34	1.34	567	24.78	Non Essential
Rv3842c	1213	31.62	776	22.43	Non Essential
Rv3843c	1095	22.88	943	21.85	Non Essential
Rv3844	35	1.53	32	1.55	Non Essential
Rv3845	9	0.54	13	0.86	Non Essential

Rv3846	43	1.48	84	3.21	Non Essential
Rv3847	393	15.84	404	18.05	Non Essential
Rv3848	100	2.37	59	1.55	Non Essential
Rv3849	980	52.89	952	56.97	Non Essential
Rv3850	331	10.84	354	12.85	Non Essential
Rv3851	6	0.45	15	1.26	Non Essential
Rv3852	611	32.49	628	37.02	Non Essential
Rv3853	84	3.81	70	3.52	Non Essential
Rv3854c	858	12.55	782	12.68	Non Essential
Rv3855	379	12.52	411	15.06	Essential
Rv3856c	251	5.35	232	5.49	Non Essential
Rv3857c	36	3.93	27	3.26	Non Essential
Rv3858c	816	11.96	766	12.44	Essential
Rv3859c	1786	8.37	1784	9.27	Essential
Rv3860	32	0.59	50	1.02	Non Essential
Rv3861	5	0.33	2	0.15	Non Essential
Rv3862c	31	1.90	55	3.74	Non Essential
Rv3863	310	5.65	357	7.22	Non Essential
Rv3864	711	12.64	625	12.32	Essential
Rv3865	558	38.54	544	41.66	Non Essential
Rv3866	869	21.93	802	22.44	Non Essential
Rv3867	1606	62.61	1199	51.82	Non Essential
Rv3868	1111	13.87	813	11.25	Essential
Rv3869	510	7.60	401	6.62	Essential
Rv3870	1456	13.94	1026	10.89	Essential
Rv3871	1978	23.94	1254	16.83	Essential
Rv3872	353	25.36	259	20.63	Essential
Rv3873	1272	24.70	1221	26.29	Essential
Rv3874	13998	995.64	8713	687.11	Non Essential
Rv3875	4931	369.06	2993	248.36	Non Essential
Rv3876	456	4.90	420	5.00	Essential
Rv3877	546	7.64	484	7.51	Essential
Rv3878	172	4.39	170	4.81	Non Essential
Rv3879c	796	7.81	565	6.15	Non Essential
Rv3879A	4	0.12	0	0.00	
Rv3880c	790	48.90	633	43.44	Non Essential
Rv3881c	3424	53.22	2946	50.77	Non Essential
Rv3882c	265	4.10	242	4.15	Essential
Rv3883c	150	2.40	161	2.86	Non Essential
Rv3884c	329	3.80	215	2.75	Non Essential
Rv3885c	659	8.78	484	7.15	Non Essential
Rv3886c	140	1.82	131	1.89	Non Essential
Rv3887c	320	4.50	243	3.78	Non Essential
Rv3888c	83	1.74	85	1.97	Non Essential
Rv3889c	109	2.82	112	3.21	Non Essential
Rv3890c	128	9.58	119	9.87	Non Essential
Rv3891c	44	2.93	37	2.73	Non Essential
Rv3892c	31	0.56	26	0.52	Non Essential
Rv3893c	335	30.88	316	32.30	Non Essential
Rv3894c	609	3.12	604	3.43	Non Essential

Rv3895c	102	1.47	98	1.57	Non Essential
Rv3896c	101	2.39	83	2.18	Non Essential
Rv3897c	204	6.93	224	8.44	Non Essential
Rv3898c	53	3.43	49	3.51	Non Essential
Rv3899c	140	2.44	149	2.88	Non Essential
Rv3900c	203	4.66	213	5.43	Non Essential
Rv3901c	89	4.26	109	5.78	Non Essential
Rv3902c	48	1.95	61	2.74	Non Essential
Rv3903c	196	1.66	224	2.10	Non Essential
Rv3904c	19	1.50	17	1.49	Non Essential
Rv3905c	40	2.76	37	2.83	Non Essential
Rv3906c	196	8.27	194	9.08	Non Essential
Rv3907c	290	4.32	337	5.57	Essential
Rv3908	502	14.45	590	18.84	Non Essential
Rv3909	202	1.80	195	1.93	Non Essential
Rv3910	662	4.00	566	3.79	Essential
Rv3911	28	0.90	26	0.93	Non Essential
Rv3912	7	0.20	6	0.19	Non Essential
Rv3913	98	2.09	114	2.70	Essential
Rv3914	35	2.15	45	3.06	Non Essential
Rv3915	697	12.27	657	12.83	Essential
Rv3916c	270	7.90	155	5.03	Non Essential
Rv3917c	617	12.82	536	12.35	Essential
Rv3918c	325	6.69	249	5.69	Essential
Rv3919c	979	31.20	738	26.08	Non Essential
Rv3920c	3796	144.83	2970	125.64	Non Essential
Rv3921c	2156	42.10	1974	42.74	Essential
Rv3922c	299	17.74	296	19.47	Non Essential
Rv3923c	1003	57.15	1110	70.12	Essential
Rv3924c	104	15.62	120	19.99	Non Essential

Table S2. Oligonucleotides used in this study.

Name	Sequence (5' to 3')	Purpose
1955T F	gtattaattaagccccgtccaagccggaca	Amplification of Rv1955
1955T R	ggggaattctcagatcggtggggtgtcgcc	Amplification of Rv1955
Taq16S F	aagaagcaaccggccaactac	qRT-PCR for 16S
Taq16S R	tcgctcctgagcgtgagtta	qRT-PCR for 16S
TaqSsrAb F	gccggtgcaggcaaga	qRT-PCR for 5' *ssrA*
TaqSsrAb R	ctgatgtgaatcggcgctta	qRT-PCR for 5' *ssrA*
TaqSsrAe F	ggactcctcgggacaacca	qRT-PCR for 3' *ssrA*
TaqSsrAe R	ggcattccctcaaggcttct	qRT-PCR for 3' *ssrA*
TaqsigA F	tcggttcgcgcctacct	qRT-PCR for *sigA*
TaqsigA R	ggctagctcgacctcttcct	qRT-PCR for *sigA*
NB5S	gtcccattccgaacccggaagctaagcctgccagcgcctgtctc	template for 5S probe
NBssrA 5'	gaccaccgtaagcgtcgttgcgaccaaataagcctgtctc	template for 5'*ssrA* probe
NBssrA 3'	cagcgactgggatcgtcatctcggctagttcgcctgtctc	template for 3' *ssrA* probe
GSP 1	cagcgactgggatcgtcatctcggctagttcgcctgtctc	template for 3' *ssrA* probe

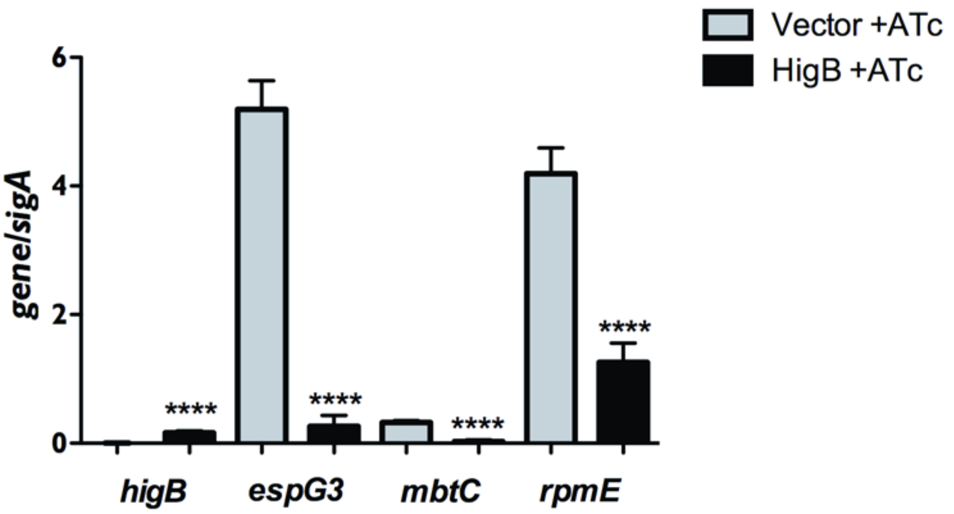

Fig. S1. Quantitative RT-PCR analysis of *higB* expression in *M. tuberculosis* ΔTAC strains.

M. tuberculosis ΔTAC strains carrying vector control (“Vector”) or the HigB expression plasmid (“HigB”) were grown to mid-exponential phase and treated with 300 ng mL-1 ATc. RNA was isolated 24 h after addition of ATc. Quantitative RT-PCR analysis was carried out relative to sigA mRNA. All results are the mean values and standard deviation of three independent biological replicates. A significant difference (as determined by Student’s t-test) between strains is marked by **** for p<0.0001.

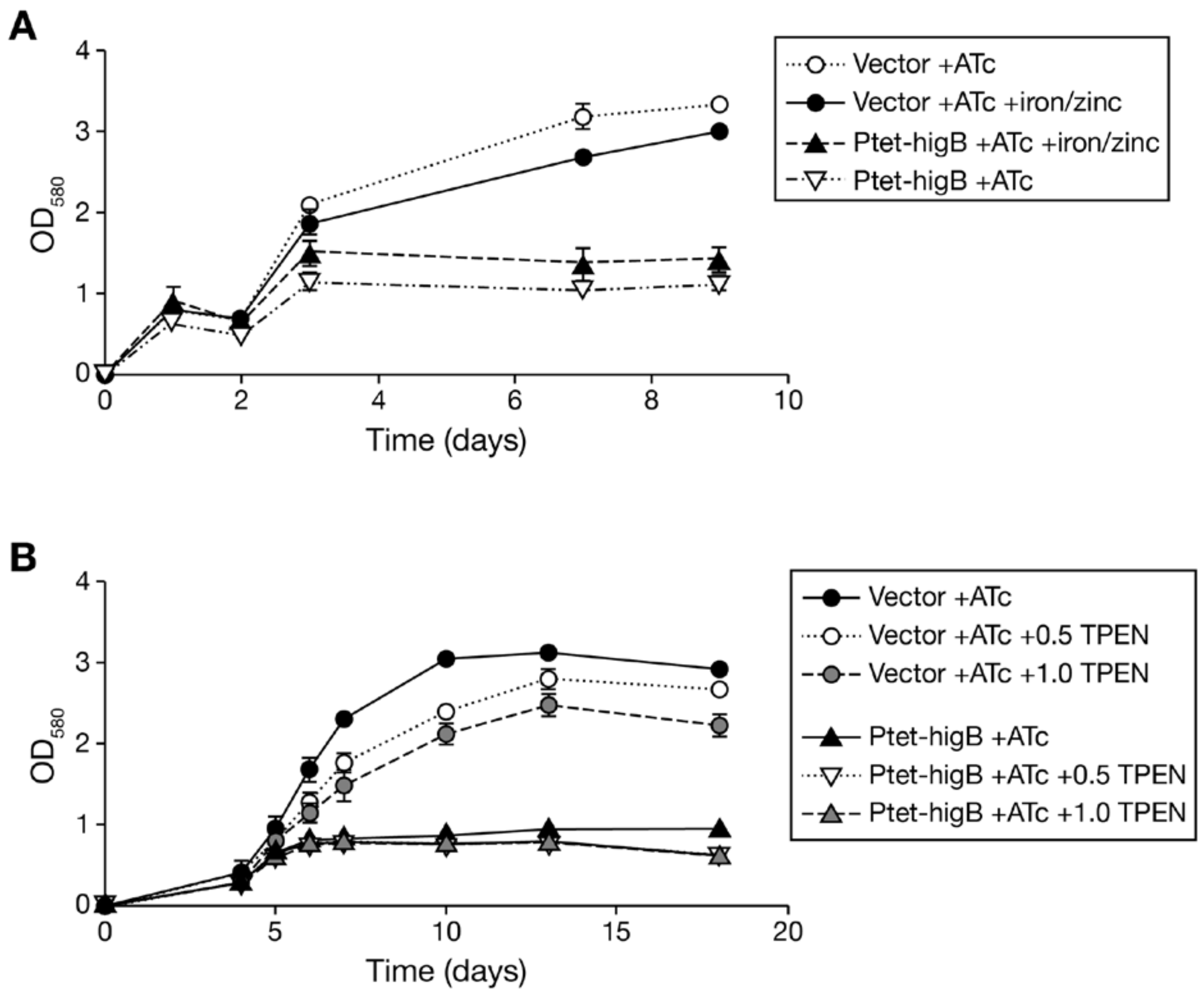

Figure S2. HigB expression in strains grown in media with varying metal ion concentrations. Strains carrying vector control (“Vector”) or the HigB expression plasmid (“HigB”) were grown to OD ~ 0.7 and inducer was added. A. Growth in Dubos medium. Upon addition of ATc, medium was also supplemented with 300 µM ferric ammonium citrate and 30 µM zinc sulphate heptahydrate (“+ zinc/iron”) to increase the concentration of iron and zinc in the medium. B. Growth in Sauton’s medium. Upon addition of ATc, liquid cultures were supplemented with 0.5 µM (“ + 0.5 TPEN”) or 1 µM (“ + 1.0 TPEN”) of the zinc chelator TPEN (Cayman Chemical) to deplete the amount of zinc in the medium.
